# Integrated Chemical, In Silico, and Functional Neurobehavioral Evaluation of Three Essential Oils in Acute Anxiety- and Depression-Related Mouse Models

**DOI:** 10.3390/molecules31132378

**Published:** 2026-07-06

**Authors:** Marilú Roxana Soto-Vásquez, Paul Alan Arkin Alvarado-García, Demetrio Rafael Jara-Aguilar, José Gilberto Gavidia-Valencia, Segundo Guillermo Ruiz-Reyes, Roger Antonio Rengifo-Penadillos

**Affiliations:** 1 Grupo de Investigación en Productos Naturales y Sustancias Bioactivas, Facultad de Farmacia y Bioquímica, Universidad Nacional de Trujillo, Av. Juan Pablo II s/n, Trujillo 13011, Peru; 2 Facultad de Ciencias de la Salud, Universidad Autónoma del Perú, Panamericana Sur Km. 16.3, Villa EI Salvador, Lima 15801, Peru; 3 Facultad de Ciencias de la Salud, Universidad Católica de Trujillo, Panamericana Norte Km. 555, Moche, Trujillo 13600, Peru

**Keywords:** essential oils, *Satureja brevicalyx*, *Peperomia dolabriformis*, *Rosmarinus officinalis*, anxiolytic-like activity, antidepressant-like activity, molecular docking, flumazenil, WAY-100635, corticosterone

## Abstract

Essential oils are multicomponent natural products with potential neurobehavioral activity, but integrated comparative studies remain limited. This study compared the essential oils of *Satureja brevicalyx*, *Peperomia dolabriformis*, and *Rosmarinus officinalis* in relation to their chemical profiles, predicted target interactions, preliminary acute oral safety, anxiolytic-like and antidepressant-like effects, antagonist-sensitive behavioral patterns, and exploratory serum biomarkers. Oils were characterized by GC-MS, and their constituents were screened by molecular docking against anxiety-, depression-, sleep-, and stress-related targets. Independent cohorts of male BALB/c mice received oral essential oils (25–100 mg/kg) and were assessed in anxiety-related, depression-related, and locomotor behavioral paradigms, including the elevated plus maze, light–dark box, marble burying, tail suspension, forced swim, and open field tests. Flumazenil and WAY-100635 were used to examine whether the behavioral responses were sensitive to γ-aminobutyric acid type A (GABA-A)/benzodiazepine- and serotonin 1A (5-HT1A)-related pharmacological modulation, respectively. In a preliminary 24-h acute oral toxicity screen, no mortality was observed up to 5000 mg/kg. The three oils produced anxiolytic-like and antidepressant-like effects without reducing spontaneous locomotor activity. Within its experimental block, *S. brevicalyx* showed the most consistent flumazenil-sensitive anxiolytic-like pattern and FDR-significant reductions in corticosterone and TNF-α, together with increased IL-4. *P. dolabriformis* showed a broader predicted multitarget docking profile and antagonist-sensitive behavioral attenuation compatible with mixed pathway participation. *R. officinalis* produced significant but more moderate behavioral effects. WAY-100635 partially attenuated the antidepressant-like effects of all three oils. These findings support differentiated but convergent functional neurobehavioral profiles among the oils. The docking, antagonist, and biomarker results should be interpreted as hypothesis-generating evidence of possible pathway involvement, supporting further validation in chronic stress models, receptor-specific assays, pharmacokinetic studies, and expanded safety evaluations.

## 1. Introduction

Mental disorders are a major and growing public health challenge worldwide, with anxiety and depression representing the most prevalent and disabling conditions [1]. The Global Burden of Disease Study 2021 revealed 444 million mental disorder incidents and 155 million disability-adjusted life years (DALYs), with major depressive disorder and anxiety disorders having the highest age-standardized DALY rates [2]. In addition, these conditions significantly hinder social functioning, academic and occupational performance, and overall quality of life, resulting in considerable economic losses due to diminished productivity and substantial treatment gaps, as access to effective, high-quality mental health care remains insufficient in many areas [3,4].

In this context, individuals with stress-related affective conditions are particularly vulnerable to large-scale psychosocial disruption [5]. Moreover, conventional pharmacotherapy remains limited by delayed onset of action, incomplete remission in a substantial proportion of patients, and tolerability concerns that continue to motivate the search for complementary or novel therapeutic strategies [6].

Within this context, essential oils have emerged not only as empirically used aromatherapeutic products but also as chemically complex natural mixtures capable of modulating behavioral and molecular endpoints relevant to affective dysfunction [7]. The evidence indicates that numerous essential oils and their major volatile constituents exhibit anxiolytic-like and antidepressant-like effects in rodents,4 and that this body of work increasingly links such effects to neuropharmacological mechanisms involving GABAergic modulation, monoaminergic pathways, and other central targets [8,9,10]. However, the available evidence remains markedly heterogeneous across plant identity, chemotype, route of administration, dosing strategy, behavioral paradigms, and depth of mechanistic validation, limiting direct cross-study comparability and making it difficult to determine which oils are the most promising candidates for deeper translational development.

A stronger mechanistic framework is particularly important because anxiety- and depression-related states are biologically complex and cannot be explained by a single pathway [11]. Contemporary models integrate monoaminergic dysregulation, hypothalamic–pituitary–adrenal (HPA) axis dysfunction, neuroinflammatory signaling, and deficits in GABAergic neurotransmission as interacting components of affective pathophysiology [12]. From this perspective, essential oils are especially attractive for investigation because their multicomponent nature raises the possibility of multitarget actions across several of these systems rather than a narrow single-target effect [13].

Aromatic plant materials, whether obtained from wild species or cultivated medicinal plants, represent important sources of volatile natural products with potential neuropharmacological relevance. In Peru, diverse ecological and agronomic conditions may contribute to chemical variability in essential oils, yet locally collected aromatic plant materials remain unevenly characterized in terms of chemical composition, behavioral effects, and mechanism-related neuropharmacological profiles [14].

The three species were selected as a comparative panel because they belong to aromatic taxa for which neurobehavioral activity has already been suggested at the genus or species level, although with varying levels of evidence. Previous studies suggest that *Satureja brevicalyx* has anxiolytic effects [15]. Within the *Satureja* genus, anxiolytic- and antidepressant-related effects have been reported for *S. montana* dry extract [16]. In addition, *S. bachtiarica* essential oil effectively ameliorated reserpine-induced depression in rats [17]. *Rosmarinus officinalis* served as a more extensively studied comparator, given the broader evidence supporting the anti-stress, anxiolytic-like, and antidepressant-like effects of its essential oil or rosemary-derived preparations in experimental settings [18,19,20]. In contrast, the use of *Peperomia dolabriformis* remains less developed as a neurobehavioral source, although early data indicate that its hydroalcoholic extract has antidepressant-like activity [21] and that its sedative effects have been reported for *P. galioides* [22]. This combination, therefore, provided a rational comparative framework spanning better-characterized and still emerging neuroactive botanicals.

Despite growing interest in plant volatiles for mood-related research, a clear knowledge gap remains: few studies compare multiple essential oils within a single design that simultaneously integrates GC-MS chemical profiling, in silico target profiling, in vivo neurobehavioral phenotyping, and mechanism-relevant biological readouts. Recent work in the field has shown the value of combining essential-oil screening with cell-based neuroprotection or neuroinflammation assays, behavioral testing, and target-oriented computational analysis, as well as the ability to link chemical composition to stress-related behavioral impairment in animal models [23,24]. Nevertheless, to our knowledge, no study has yet applied this integrative comparative logic to the essential oils of *S. brevicalyx*, *P. dolabriformis*, and *R. officinalis* in experimental models relevant to anxiety- and depression-related behaviors.

Accordingly, the aim of the present study was to perform an integrated chemical, in silico, and functional neurobehavioral evaluation of the essential oils of *Satureja brevicalyx*, *Peperomia dolabriformis*, and *Rosmarinus officinalis* in acute mouse models related to anxiety- and depression-like behaviors. Specifically, we sought to compare their GC-MS-based chemical fingerprints, examine putative ligand–target interactions through molecular docking, assess their acute oral neurobehavioral effects in vivo, and explore antagonist-sensitive behavioral patterns together with peripheral endocrine–immune biomarkers. This comparative approach was designed to identify differentiated activity profiles among the three oils and to generate pathway-level hypotheses for subsequent pharmacological, toxicological, and translational validation.

## 2. Results

### 2.1. Chemical Composition of the Essential Oils

Gas chromatography-mass spectrometry (GC-MS) profiling provided distinct semi-quantitative chemical fingerprints for the essential oils of *Satureja brevicalyx* (SBEO), *Rosmarinus officinalis* (ROEO), and *Peperomia dolabriformis* (PDEO). The comparative profile of major GC-MS-annotated constituents, defined as compounds with a relative TIC-area abundance ≥1.0% in at least one essential oil, is summarized in Table 1. Representative TIC chromatograms supporting the GC-MS profiles are shown in Supplementary Figures S1–S9. Overall, the three oils differed markedly not only in their dominant annotated constituents, but also in the relative contributions of monoterpenes, sesquiterpenes, and aromatic/phenylpropanoid-type compounds. Because experimentally calculated retention indices and authentic standards were not available, the chemical data should be interpreted as putative GC-MS annotations and relative TIC-area profiles.

SBEO was characterized by a linalool-rich profile, with linalool as the most abundant constituent (21.37%). Other major compounds included pulegone (11.24%), menthone (10.41%), limonene (10.01%), bicyclogermacrene (8.11%), β-caryophyllene (7.99%), γ-terpinene (6.10%), and isomenthone (5.95%). Minor but relevant constituents included α-terpineol (3.40%), trans-dihydrocarvone (2.82%), and *o*-cymene (1.52%). This composition indicates that SBEO contains a mixed monoterpenoid–sesquiterpenoid profile, with oxygenated monoterpenes and monoterpene ketones representing a prominent fraction of the oil.

ROEO was characterized predominantly by 1,8-cineole/eucalyptol (27.40%) and α-terpineol (16.38%), followed by limonene (10.81%), α-pinene (7.32%), linalool (6.75%), camphor (5.05%), β-pinene (4.93%), bornyl acetate (4.58%), and camphene (4.02%). β-Myrcene was also detected at 1.39%. Compared with the other oils, *R. officinalis* displayed a typical oxygenated monoterpene-rich profile, with 1,8-cineole and α-terpineol accounting for a large proportion of the annotated TIC-area fraction.

In contrast, *PDEO* exhibited a more distinctive aromatic and sesquiterpenoid profile. The largest TIC-area peak was annotated as 5-tert-butyl-1,3-benzodioxole (23.19%); however, because this assignment was not confirmed using an authentic standard or experimentally calculated retention index, it should be considered tentative. Other major annotated constituents included limonene (13.60%), myristicin (10.83%), ishwarane (8.83%), ishwarol B (6.11%), and elemicin (5.49%). Additional constituents present above 1% included n-butylbenzene (4.25%), orcinol dimethyl ether (4.05%), τ-cadinol (3.84%), β-pinene (1.64%), α-pinene (1.35%), eremophilene (1.24%), nonane (1.02%), and γ-amorphene (1.00%). These findings suggest that *P. dolabriformis* differs substantially from the two Lamiaceae oils in terms of the marked contribution of aromatic/phenylpropanoid-type and sesquiterpenoid constituents, although the identity of selected PDEO constituents requires confirmation in future analyses using authentic standards and experimentally determined retention indices.

Several constituents were shared among the three oils, although at different relative abundances. Limonene was detected as a major compound in all three essential oils, with comparable but not identical proportions in *S. brevicalyx* (10.01%), *R. officinalis* (10.81%), and *P. dolabriformis* (13.60%). α-Pinene and β-pinene were also present across the three oils, but they were more abundant in *R. officinalis* and *P. dolabriformis* than in *S. brevicalyx*. Linalool was abundant in *S. brevicalyx* and *R. officinalis* but was not detected among the reported major constituents of *P. dolabriformis*. Conversely, myristicin, elemicin, ishwarane, and ishwarol B were characteristic of *P. dolabriformis*, whereas pulegone, menthone, isomenthone, and bicyclogermacrene were mainly associated with *S. brevicalyx*.

### 2.2. Chemical Class Distribution

**Figure 1:**
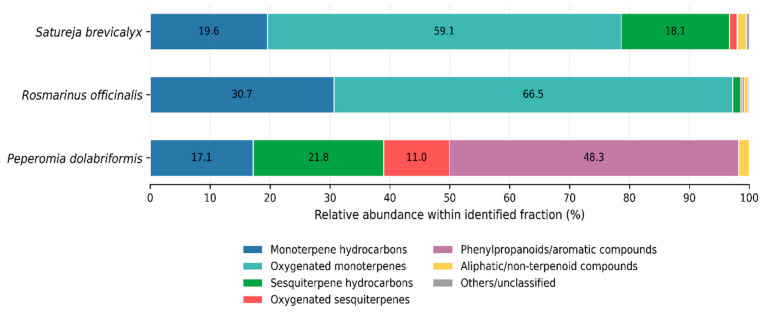
Chemical class distribution of the essential oils of *Satureja brevicalyx*, *Rosmarinus officinalis*, and *Peperomia dolabriformis*. The stacked bars show the relative abundance of the main annotated chemical classes, normalized to the sum of the GC-MS-annotated TIC-area percentages in each essential oil. The chemical classes included monoterpene hydrocarbons, oxygenated monoterpenes, sesquiterpene hydrocarbons, oxygenated sesquiterpenes, phenylpropanoids/aromatic compounds, aliphatic/nonterpenoid compounds, and others/unclassified compounds. Percentages were calculated from the GC-MS annotation tables and expressed relative to the annotated TIC-area fraction of each oil; therefore, each bar sums to 100%.

The chemical class distribution, based on putative GC-MS annotations and relative TIC-area abundances, further highlighted compositional differences among the three essential oils (Figure 1). SBEO and ROEO were dominated by oxygenated monoterpenes, which represented 59.06% and 66.55% of the annotated TIC-area fraction, respectively. However, the secondary chemical classes differed between the oils. In SBEO, oxygenated monoterpenes were accompanied by relevant proportions of monoterpene hydrocarbons (19.56%) and sesquiterpene hydrocarbons (18.08%). In ROEO, monoterpene hydrocarbons represented 30.66%, whereas sesquiterpene hydrocarbons and oxygenated sesquiterpenes were present only at low relative TIC-area levels.

In contrast, PDEO displayed a markedly different annotated chemical-class distribution, with phenylpropanoids/aromatic compounds representing the largest relative TIC-area fraction (48.27%). This class was followed by sesquiterpene hydrocarbons (21.75%), monoterpene hydrocarbons (17.14%), and oxygenated sesquiterpenes (10.96%). Oxygenated monoterpenes accounted for only 0.08% of the annotated TIC-area fraction in this oil. Therefore, although the compound-level assignments remain putative or tentative for selected constituents, the class-level distribution clearly differentiated the two monoterpene-rich Lamiaceae oils from the chemically distinct, phenylpropanoid/aromatic compound-rich PDEO (Piperaceae) profile.

### 2.3. Molecular Docking Profile of Major GC-MS-Annotated Constituents

**Figure 2:**
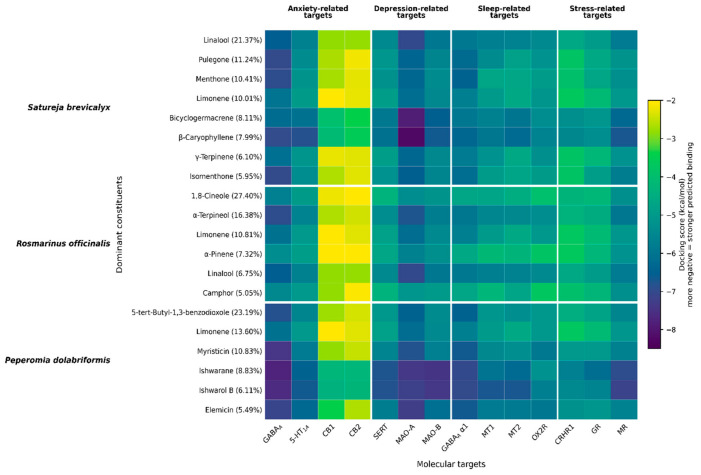
Heatmap of molecular docking scores for the major GC-MS-annotated constituents of the essential oils of *Satureja brevicalyx*, *Rosmarinus officinalis*, and *Peperomia dolabriformis* across neurobehavioral-related molecular targets. Major annotated constituents were defined as compounds whose relative TIC-area abundance was ≥5% in the corresponding GC-MS profile. The targets were grouped into anxiety-related (GABA-A, 5-HT1A, CB1, and CB2), depression-related (SERT, MAO-A, and MAO-B), sleep-related (GABA-A α1, MT1, MT2, and OX2R), and stress-related (CRHR1, GR, and MR) pathways. Docking scores are expressed as predicted binding energies in kcal/mol; more negative values indicate more favorable predicted scores within the docking model. The heatmap is intended as exploratory in silico evidence of potential ligand–target compatibility and does not establish confirmed binding, receptor activation or inhibition, enzyme inhibition, or pharmacological efficacy. Because selected constituent annotations, particularly in PDEO, remain putative or tentative, the docking results should be interpreted as hypothesis-generating target-compatibility profiles.

The molecular docking heatmap shown in Figure 2 was generated using the major GC-MS-annotated constituents of each essential oil, defined as compounds with relative TIC-area abundances ≥5% in the corresponding GC-MS profile. This selection included eight constituents from SBEO, six from ROEO, and six from PDEO. Predicted docking scores were evaluated across molecular targets associated with anxiety-related, depression-related, sleep-related, and stress-related pathways. Because docking scores are target- and model-dependent and do not directly establish binding, receptor activation, antagonism, enzyme inhibition, or pharmacological efficacy, these results were interpreted as exploratory evidence of potential ligand–target compatibility.

Overall, the docking analysis suggests that the major constituents of the three essential oils differed in their predicted multitarget interaction profiles. The most favorable individual docking score in the dataset was observed for β-caryophyllene from SBEO against MAO-A (−8.22 kcal/mol), followed by bicyclogermacrene from the same oil against MAO-A (−7.89 kcal/mol). These two sesquiterpene hydrocarbons also showed relatively favorable predicted scores for other targets, particularly 5-HT1A, MAO-B, GABA-A-related targets, and mineralocorticoid receptor-related targets. In contrast, the monoterpenoid constituents of SBEO, including linalool, pulegone, menthone, limonene, γ-terpinene, and isomenthone, showed a more moderate but consistent predicted docking profile, especially toward GABA-A, GABA-A α1, MAO-A, and MAO-B.

Among the major annotated constituents of ROEO, α-terpineol and linalool displayed the most favorable overall predicted profiles. α-Terpineol showed predicted scores of −6.95 kcal/mol for GABA-A, −6.76 kcal/mol for MAO-A, −5.98 kcal/mol for GABA-A α1, and −5.94 kcal/mol for MR. Linalool showed a similar pattern, with favorable predicted docking scores for MAO-A (−7.03 kcal/mol), GABA-A (−6.55 kcal/mol), MAO-B (−5.94 kcal/mol), GABA-A α1 (−5.91 kcal/mol), and 5-HT1A (−5.68 kcal/mol). By comparison, 1,8-cineole, despite being the largest TIC-area constituent in R. officinalis, showed a more moderate predicted docking profile across the target panel.

The major annotated constituents of PDEO showed the broadest predicted multitarget docking pattern in the heatmap. Ishwarane and ishwarol B contributed prominently to this predicted profile, with relatively favorable scores across several GABA-A-, monoamine-, melatonin-, and stress-related targets. Ishwarane showed predicted scores for GABA-A (−7.78 kcal/mol), MAO-B (−7.45 kcal/mol), MAO-A (−7.37 kcal/mol), GABA-A α1 (−7.05 kcal/mol), and MR (−6.94 kcal/mol). Ishwarol B also displayed favorable predicted scores across several targets, including GABA-A (−7.63 kcal/mol), MAO-B (−7.37 kcal/mol), MAO-A (−7.17 kcal/mol), MR (−7.14 kcal/mol), GABA-A α1 (−7.01 kcal/mol), MT1 (−6.71 kcal/mol), MT2 (−6.72 kcal/mol), SERT (−6.80 kcal/mol), and 5-HT1A (−6.65 kcal/mol). Myristicin and elemicin also contributed to this predicted pattern, particularly through docking scores involving GABA-A, MAO-A, GABA-A α1, and OX2R. However, because several PDEO annotations remain putative or tentative and docking was not experimentally validated, these results should be interpreted as hypothesis-generating pathway-level patterns rather than as evidence of confirmed target interactions or functional activity.

Across target categories, the most favorable predicted docking scores were concentrated mainly in the monoaminergic and GABA-A-related targets. MAO-A showed favorable predicted values for several major annotated constituents, including β-caryophyllene, bicyclogermacrene, ishwarane, ishwarol B, elemicin, linalool, and α-terpineol. GABA-A and GABA-A α1 also showed favorable predicted scores for several compounds, including ishwarane, ishwarol B, myristicin, elemicin, β-caryophyllene, pulegone, menthone, isomenthone, α-terpineol, and linalool. In contrast, CB1 and CB2 generally showed weaker predicted scores across the dataset, suggesting that cannabinoid receptor-related docking was less prominent in this exploratory screen.

For sleep-related targets, the predicted scores were moderate overall but showed compound-specific patterns. Ishwarol B displayed the most favorable predicted scores for MT1 and MT2, whereas ishwarane and ishwarol B showed the most favorable scores for GABA-A α1. OX2R-related docking scores were weaker than those observed for GABA-A- and monoaminergic targets, although elemicin and myristicin showed the most favorable predicted values within this target. For stress-related targets, ishwarane and ishwarol B had the most favorable predicted scores for GR and MR, whereas CRHR1 displayed more moderate predicted values across the evaluated compounds.

Taken together, the docking results suggest that the major GC-MS-annotated constituents of the three essential oils may differ in their predicted neurobehavioral target-compatibility patterns. SBEO was characterized mainly by linalool- and monoterpene ketone-associated predicted scores involving GABA-A- and monoamine-related targets, together with favorable MAO-A docking by β-caryophyllene and bicyclogermacrene. ROEO showed a more moderate predicted profile, with α-terpineol and linalool contributing the most favorable predicted scores. PDEO displayed the broadest predicted multitarget pattern, largely driven by the putatively annotated constituents ishwarane, ishwarol B, myristicin, and elemicin. These findings do not establish a definitive mechanism of action but provide a rationale for prioritizing GABA-A-, 5-HT1A-, and monoamine-related pathways in subsequent experimental pharmacological validation.

### 2.4. Preliminary Acute Oral Toxicity Profile

A preliminary acute oral toxicity assessment revealed no mortality for any of the three essential oils at doses ranging from 10 to 5000 mg/kg within the 24-h observation window (Table 2; Supplementary Table S8). No abnormal clinical signs were observed during phase 1 at 10, 100, or 1000 mg/kg. In Phase 2, mild and transient clinical signs were observed only at higher dose levels. SBEO produced mild piloerection, reduced grooming, hypoactivity, and mild ataxia at 1600–5000 mg/kg, with recovery by 24 h. PDEO produced mild-to-moderate transient hypoactivity, mild ataxia, and reduced grooming at 1600–5000 mg/kg, with recovery by 24 h. ROEO produced mild-to-moderate transient hypoactivity, reduced grooming, and piloerection at 1600–5000 mg/kg, with recovery by 24 h. Because no mortality was observed at the highest tested dose, the oral LD50 was estimated to be greater than 5000 mg/kg under the conditions of this preliminary 24-h acute toxicity assessment.

### 2.5. Anxiolytic-like Behavioral Effects

**Figure 3:**
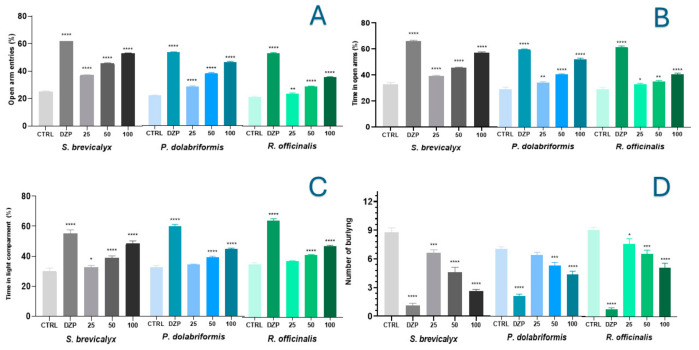
Anxiolytic-like behavioral effects of the three essential oils in mice. Effects of *Satureja brevicalyx*, *Peperomia dolabriformis*, and *Rosmarinus officinalis* essential oils on anxiety-related behavioral outcomes. (**A**) Percentage of open arm entries in the elevated plus maze. (**B**) Percentage of time spent in the open arms in the elevated plus maze. (**C**) Percentage of time spent in the light compartment in the light–dark box test. (**D**) Number of marbles buried in the marble burying test. Each essential oil was evaluated in an independent experimental block with its corresponding vehicle control and diazepam control. CTRL = vehicle-treated control; DZP = 1 mg/kg diazepam; 25, 50, and 100 indicate essential oil doses in mg/kg. The bars represent the mean ± SEM; *n* = 8 animals per group. * *p* < 0.05, ** *p* < 0.01, *** *p* < 0.001, and **** *p* < 0.0001 versus the corresponding CTRL group; ordinary one-way ANOVA followed by Dunnett’s multiple comparisons test.

All three essential oils produced anxiolytic-like effects on the behavioral battery, although with different magnitudes across tests and doses (Figure 3). In the behavioral results, CTRL denotes the vehicle-treated control group. In the elevated plus-maze test, SBEO robustly increased open-arm exploration in a dose-dependent manner. With respect to the percentage of open arm entries, one-way ANOVA revealed a significant treatment effect [F(4,35) = 1381, *p* < 0.0001]. Compared with the CTRL (25.27%), diazepam increased the number of open arm entries to 61.70% (*p* < 0.0001), whereas SBEO increased this parameter to 37.07%, 45.63%, and 53.07% at 25, 50, and 100 mg/kg, respectively (all *p* < 0.0001 vs. CTRL). A similar pattern was observed for the percentage of time spent in the open arms [F(4,35) = 214.4, *p* < 0.0001], with values increasing from 32.67% in the CTRL group to 66.09% with diazepam and to 39.25%, 45.38%, and 57.07% with SBEO at 25, 50, and 100 mg/kg, respectively (all *p* < 0.0001 vs. CTRL). In the light–dark box, SBEO also increased the percentage of time spent in the light compartment [F(4,35) = 285.1, *p* < 0.0001], from 29.93% in the CTRL to 32.41% at 25 mg/kg (*p* = 0.0307), 38.99% at 50 mg/kg (*p* < 0.0001), and 48.61% at 100 mg/kg (*p* < 0.0001). In the marble burying test, SBEO significantly reduced the number of buried marbles [F(4,35) = 68.50, *p* < 0.0001], from 8.75 in the CTRL to 6.63, 4.63, and 2.63 at 25, 50, and 100 mg/kg, respectively (*p* = 0.0009, *p* < 0.0001, and *p* < 0.0001 vs. CTRL, respectively).

PDEO also showed a clear anxiolytic-like profile, particularly in the elevated plus-maze test. For open arm entries, a significant treatment effect was detected [F(4,35) = 745.2, *p* < 0.0001]. PDEO increased the number of open arm entries from 24.24% in the CTRL group to 31.62%, 42.25%, and 51.02% at 25, 50, and 100 mg/kg, respectively (all *p* < 0.0001 vs. the CTRL group), whereas diazepam increased this value to 58.82% (*p* < 0.0001). For time spent in the open arms, ANOVA also revealed a significant treatment effect [F(4,35) = 185.5, *p* < 0.0001]. PDEO increased the open-arm time from 31.42% in the CTRL group to 37.08% in the 25 mg/kg group (*p* = 0.0012), 43.59% in the 50 mg/kg group (*p* < 0.0001), and 56.37% in the 100 mg/kg group (*p* < 0.0001) compared with 64.45% in the diazepam group (*p* < 0.0001 vs. CTRL). In the light–dark box, PDEO had a significant overall effect [F(4,35) = 167.5, *p* < 0.0001], but the effect of the 25 mg/kg dose did not differ from that of the CTRL (30.85% vs. 29.43%; *p* = 0.4878). In contrast, 50 and 100 mg/kg increased the percentage of time spent in the light compartment to 35.27% and 40.21%, respectively (both *p* < 0.0001 vs. CTRL). In the marble burying test, PDEO significantly reduced defensive digging [F(4,35) = 44.77, *p* < 0.0001]. The 25 mg/kg dose did not significantly reduce the number of buried marbles (8.75 vs. 9.63 in CTRL; *p* = 0.3460), whereas 50 and 100 mg/kg reduced this value to 7.25 and 6.00, respectively (*p* = 0.0006 and *p* < 0.0001 vs. CTRL).

ROEO produced significant but relatively moderate anxiolytic-like effects. In the elevated plus-maze test, ROEO significantly increased the percentage of open arm entries [F(4,35) = 550.4, *p* < 0.0001] from 22.81% in the CTRL group to 25.79%, 31.47%, and 38.78% at 25, 50, and 100 mg/kg, respectively (*p* = 0.0044, *p* < 0.0001, and *p* < 0.0001 vs. CTRL, respectively). Diazepam increased this parameter to 57.98% (*p* < 0.0001 vs. CTRL). ROEO also increased the percentage of time spent in the open arms [F(4,35) = 141.7, *p* < 0.0001], from 31.42% in the CTRL group to 35.85%, 37.96%, and 44.15% at 25, 50, and 100 mg/kg, respectively (*p* = 0.0368, *p* = 0.0013, and *p* < 0.0001 vs. CTRL, respectively), whereas diazepam increased this value to 66.58% (*p* < 0.0001). In the light–dark box, ROEO had a significant treatment effect [F(4,35) = 183.8, *p* < 0.0001]. The 25 mg/kg dose did not significantly increase time in the light compartment (31.08% vs. 29.43% in CTRL; *p* = 0.3194), whereas 50 and 100 mg/kg increased this parameter to 34.63% and 39.49%, respectively (both *p* < 0.0001 vs. CTRL). In the marble burying test, ROEO significantly reduced the number of buried marbles [F(4,35) = 68.27, *p* < 0.0001], from 8.63 in the CTRL to 7.25, 6.25, and 4.88 at 25, 50, and 100 mg/kg, respectively (*p* = 0.0390, *p* = 0.0002, and *p* < 0.0001 vs. CTRL, respectively).

Overall, SBEO showed the most consistent anxiolytic-like profile across the four behavioral endpoints, with marked effects on both elevated plus-maze measures, light–dark exploration, and marble burying. PDEO had strong effects on the elevated plus-maze test and significant effects at 50 and 100 mg/kg in the light–dark and marble burying tests, whereas ROEO had a significant but more moderate effect, which was evident mainly at 50 and 100 mg/kg.

### 2.6. Antidepressant-like Behavioral Effects

**Figure 4:**
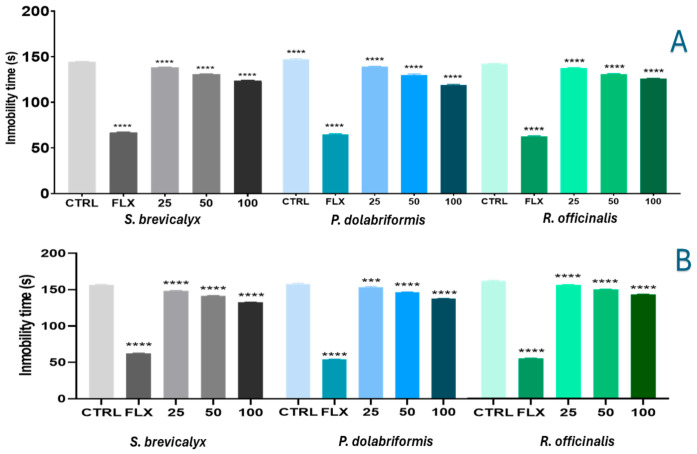
Antidepressant-like behavioral effects of the three essential oils in mice. Effects of *Satureja brevicalyx*, *Peperomia dolabriformis*, and *Rosmarinus officinalis* essential oils on depression-related behavioral outcomes. (**A**) Immobility time in the tail suspension test. (**B**) Immobility time in the forced swim test. Each essential oil was evaluated in an independent experimental block with its corresponding vehicle control and fluoxetine control groups. CTRL = vehicle-treated control; FLX = fluoxetine 10 mg/kg, i.p., used as the positive antidepressant control; 25, 50, and 100 indicate essential oil doses in mg/kg. The bars represent the mean ± SEM; *n* = 8 animals per group. A lower immobility time indicates an antidepressant-like effect. *** *p* < 0.001, and **** *p* < 0.0001 versus the corresponding CTRL group; ordinary one-way ANOVA followed by Dunnett’s multiple comparisons test.

All three essential oils reduced immobility time in the tail suspension test (TST) and forced swim test (FST), indicating antidepressant-like activity (Figure 4). FLX was used as the positive antidepressant control. In the TST, SBEO had a significant treatment effect [F(4,35) = 2826, *p* < 0.0001]. Compared with the CTRL (144.3 s), fluoxetine reduced the immobility time to 67.13 s (*p* < 0.0001), whereas SBEO reduced the immobility time to 138.1, 130.6, and 123.6 s at 25, 50, and 100 mg/kg, respectively (all *p* < 0.0001 vs. CTRL). In the forced swim test, SBEO also had a significant effect [F(4,35) = 3649, *p* < 0.0001], decreasing immobility from 156.5 s in the CTRL group to 148.5, 141.6, and 132.5 s at 25, 50, and 100 mg/kg, respectively (all *p* < 0.0001 vs. the CTRL group), whereas fluoxetine reduced immobility to 62.50 s (*p* < 0.0001). PDEO showed a marked antidepressant-like profile in both paradigms. In the tail suspension test, one-way ANOVA revealed a significant treatment effect [F(4,35) = 1733, *p* < 0.0001]. PDEO reduced immobility time from 145.8 s in the CTRL group to 137.9, 128.8, and 117.9 s at 25, 50, and 100 mg/kg, respectively (all *p* < 0.0001 vs. the CTRL group), whereas fluoxetine reduced immobility to 64.25 s (*p* < 0.0001). In the forced swim test, PDEO also significantly reduced immobility [F(4,35) = 3160, *p* < 0.0001], from 162.3 s in the CTRL group to 157.5 s at 25 mg/kg (*p* = 0.0006), 150.3 s at 50 mg/kg (*p* < 0.0001), and 141.1 s at 100 mg/kg (*p* < 0.0001). Fluoxetine reduced immobility to 55.38 s (*p* < 0.0001 vs. CTRL). ROEO also produced significant antidepressant-like effects, although the magnitude was more moderate than that observed with fluoxetine. In the tail suspension test, ROEO significantly reduced immobility time [F(4,35) = 3178, *p* < 0.0001], from 145.9 s in the CTRL group to 140.8, 134.3, and 129.0 s at 25, 50, and 100 mg/kg, respectively (all *p* < 0.0001 vs. CTRL). Fluoxetine reduced immobility to 64.38 s (*p* < 0.0001). In the forced swim test, ROEO produced a significant treatment effect [F(4,35) = 4074, *p* < 0.0001], reducing immobility from 161.9 s in the CTRL group to 156.0, 149.8, and 142.9 s at 25, 50, and 100 mg/kg, respectively (all *p* < 0.0001 vs. the CTRL group), whereas fluoxetine reduced immobility to 55.13 s (*p* < 0.0001).

Overall, the three essential oils showed consistent antidepressant-like effects across both behavioral despair paradigms. The reductions in immobility were dose-related and reproducible in both the tail suspension test and the forced swim test. Numerically, among the EO100 groups, the PDEO group had the lowest immobility value in the tail suspension test, whereas the SBEO group had the lowest immobility value in the forced swim test. However, because each oil was analyzed within its own experimental block, these between-oil differences should be interpreted descriptively rather than as direct statistical comparisons.

### 2.7. Evaluation of Spontaneous Locomotor Activity

**Figure 5:**
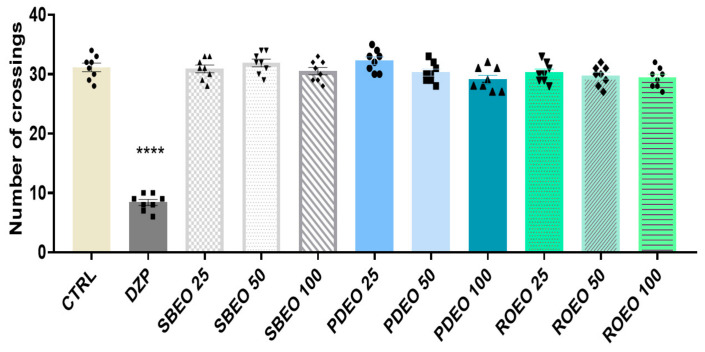
Effects of the three essential oils on spontaneous locomotor activity in the open field test. The number of squares crossed during a 5-min open-field session was used to assess spontaneous locomotor activity and potential sedative-like effects. CTRL = vehicle-treated control; DZP = 3.5 mg/kg diazepam; SBEO = *Satureja brevicalyx* essential oil; PDEO = *Peperomia dolabriformis* essential oil; ROEO = *Rosmarinus officinalis* essential oil. Doses are expressed in mg/kg. The bars represent the mean ± SEM, with individual points representing each animal; different point symbols indicate individual animals within each treatment group; *n* = 8 per group. **** *p* < 0.0001 versus CTRL; ordinary one-way ANOVA followed by Dunnett’s multiple comparisons test.

Spontaneous locomotor activity was evaluated in the open field test to determine whether the behavioral effects of the essential oils could be explained by nonspecific motor impairment or sedative-like activity (Figure 5). One-way ANOVA revealed a significant overall treatment effect on the number of crossings [F(10,77) = 117.4, *p* < 0.0001]. As expected, diazepam at the sedative motor-control dose of 3.5 mg/kg markedly reduced the locomotor activity from 31.13 crossings in the CTRL group to 8.38 crossings (*p* < 0.0001 vs. CTRL). In contrast, none of the essential oil-treated groups differed significantly from the control group. The number of crossings was 30.88, 31.88, and 30.50 for SBEO at 25, 50, and 100 mg/kg, respectively (*p* = 0.9996, *p* = 0.9699, and *p* = 0.9909 vs. CTRL); 32.25, 30.25, and 29.13 for PDEO at 25, 50, and 100 mg/kg, respectively (*p* = 0.7665, *p* = 0.9262, and *p* = 0.1641 vs. CTRL); and 30.25, 29.75, and 29.38 for ROEO at 25, 50, and 100 mg/kg, respectively (*p* = 0.9262, *p* = 0.5576, and *p* = 0.2852 vs. CTRL). These findings indicate that the anxiolytic-like and antidepressant-like effects of the essential oils were not attributable to locomotor suppression or sedative motor impairment.

### 2.8. Antagonist-Sensitive Patterns of Anxiolytic-like Effects

**Figure 6:**
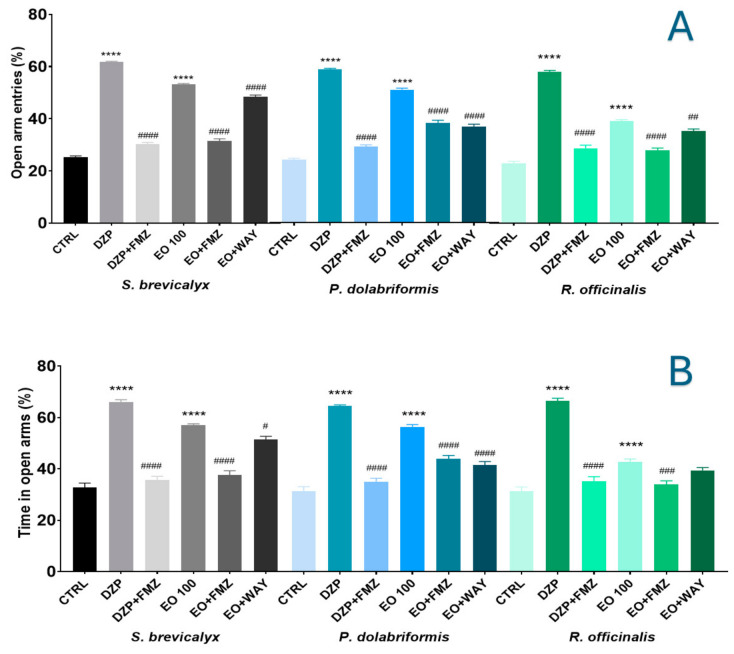
Antagonist-sensitive patterns of the anxiolytic-like effects of the three essential oils in the elevated plus maze. Flumazenil and WAY-100635 were used to assess whether the anxiolytic-like behavioral responses to *Satureja brevicalyx*, *Peperomia dolabriformis*, and *Rosmarinus officinalis essential* oils were sensitive to GABA-A/benzodiazepine- and 5-HT1A-related pharmacological modulation, respectively. (**A**) Percentage of open arm entries and (**B**) percentage of time spent in the open arms in the elevated plus maze. Each essential oil was tested at 100 mg/kg alone and in combination with the corresponding antagonist. CTRL = vehicle-treated control; DZP = 1 mg/kg diazepam; FMZ = flumazenil; WAY = WAY-100635; EO100 = 100 mg/kg essential oil. The bars represent the mean ± SEM; *n* = 8 animals per group. **** *p* < 0.0001 versus CTRL; # *p* < 0.05, ## *p* < 0.01, ### *p* < 0.001, and #### *p* < 0.0001 versus DZP or EO100, as appropriate; ordinary one-way ANOVA followed by Sidak’s selected multiple comparisons test. Antagonist-induced attenuation should be interpreted as pharmacological evidence compatible with pathway participation, not as proof of direct receptor mediation.

Antagonist coadministration assays in the elevated plus maze revealed distinct antagonist-sensitive behavioral patterns for the three essential oils (Figure 6). In this phase, EO100 denotes the corresponding essential oil administered at 100 mg/kg, and WAY denotes WAY-100635. Diazepam (DZP) significantly increased both the percentage of open arm entries (OAE) and the percentage of time spent in the open arms (OAT), and coadministration with flumazenil (FMZ) markedly attenuated the DZP effect across all experimental blocks, validating the sensitivity of the assay to gamma-aminobutyric acid type A (GABA-A)/benzodiazepine-site modulation. Because these assays were based on the antagonist-induced attenuation of behavioral responses, the findings should be interpreted as evidence compatible with possible pathway participation rather than as proof of direct receptor binding, receptor activation, or exclusive receptor mediation.

With respect to SBEO, one-way ANOVA revealed significant treatment effects for OAE [F(5,42) = 641.0, *p* < 0.0001] and OAT [F(5,42) = 105.1, *p* < 0.0001]. At the OAE endpoint, SBEO at 100 mg/kg increased the number of open arm entries from 25.30% in the CTRL group to 53.31% (*p* < 0.0001). Flumazenil strongly attenuated this effect, reducing OAEs to 31.40% (*p* < 0.0001 vs. EO100), whereas WAY-100635 produced a smaller but still significant reduction to 48.43% (*p* < 0.0001 vs. EO100). At the OAT endpoint, SBEO increased the open arm time from 32.70% in the CTRL group to 57.10% (*p* < 0.0001). Compared with the control, flumazenil reduced the OAT to 37.60% (*p* < 0.0001 vs. EO100), and this value no longer differed significantly from that of the CTRL (*p* = 0.0794). WAY-100635 resulted in only modest attenuation, reducing the OAT to 51.40% (*p* = 0.0263 vs. EO100), while the response remained significantly greater than that of the CTRL (*p* < 0.0001). This attenuation pattern is compatible with a predominantly flumazenil-sensitive anxiolytic-like response for SBEO, with a weaker WAY-100635-sensitive component; however, it does not prove direct GABA-A/benzodiazepine-site mediation or exclude the participation of additional pathways. For PDEO, significant treatment effects were detected for OAEs [F(5,42) = 317.9, *p* < 0.0001] and OAT [F(5,42) = 105.8, *p* < 0.0001]. PDEO at 100 mg/kg increased OAEs from 24.20% in the CTRL group to 51.00% (*p* < 0.0001). Coadministration with flumazenil reduced OAEs to 38.30% (*p* < 0.0001 vs. EO100), whereas that with WAY-100635 decreased the OAEs to 36.80% (*p* < 0.0001 vs. EO100). Compared with the control group, both the antagonist-combination groups remained significantly better (both *p* < 0.0001), indicating partial rather than complete attenuation. A similar pattern was observed for OAT. PDEO increased OAT from 31.40% in the CTRL to 56.40% (*p* < 0.0001), whereas flumazenil and WAY-100635 reduced this response to 43.80% and 41.50%, respectively (both *p* < 0.0001 vs. EO100). Both values remained significantly higher than those of the CTRL (both *p* < 0.0001). These findings suggest that the anxiolytic-like response to PDEO was sensitive to both flumazenil and WAY-100635, a pattern compatible with mixed GABA-A/benzodiazepine- and 5-HT1A-related pathway participation, without establishing exclusive receptor mediation. Finally, for ROEO, ANOVA revealed significant treatment effects for OAE [F(5,42) = 248.4, *p* < 0.0001] and OAT [F(5,42) = 88.27, *p* < 0.0001]. ROEO at 100 mg/kg increased the OAEs from 22.80% in the CTRL group to 39.18% (*p* < 0.0001). Flumazenil reduced the OAEs to 27.90% (*p* < 0.0001 vs. EO100), whereas WAY-100635 reduced the OAEs to 35.30% (*p* = 0.0091 vs. EO100). At the OAT endpoint, ROEO increased the open-arm time from 31.40% in the CTRL group to 42.90% (*p* < 0.0001). Flumazenil reduced this value to 34.00% (*p* = 0.0003 vs. EO100), which did not differ from that of the CTRL (*p* = 0.7664). In contrast, WAY-100635 did not significantly attenuate the ROEO-induced increase in OAT (39.50%; *p* = 0.4717 vs. EO100), and the activity in the EO + WAY group remained significantly greater than that in the CTRL group (*p* = 0.0010). Thus, ROEO showed a more moderate anxiolytic-like profile, with a clearer flumazenil-sensitive attenuation pattern and limited WAY-100635 sensitivity, particularly for open arm time.

Overall, the antagonist-sensitive profile indicated that the anxiolytic-like effects of the three essential oils were not pharmacologically identical. SBEO showed the clearest flumazenil-sensitive attenuation pattern, PDEO showed attenuation by both flumazenil and WAY-100635, and ROEO showed a predominantly flumazenil-sensitive pattern with weaker WAY-100635 sensitivity. These results support the possible participation of the GABA-A/benzodiazepine- and 5-HT1A-related pathways, but they should not be interpreted as evidence of direct receptor binding, functional receptor modulation, or exclusive pathway mediation.

### 2.9. Antagonist-Sensitive Patterns of Antidepressant-Like Effects

**Figure 7:**
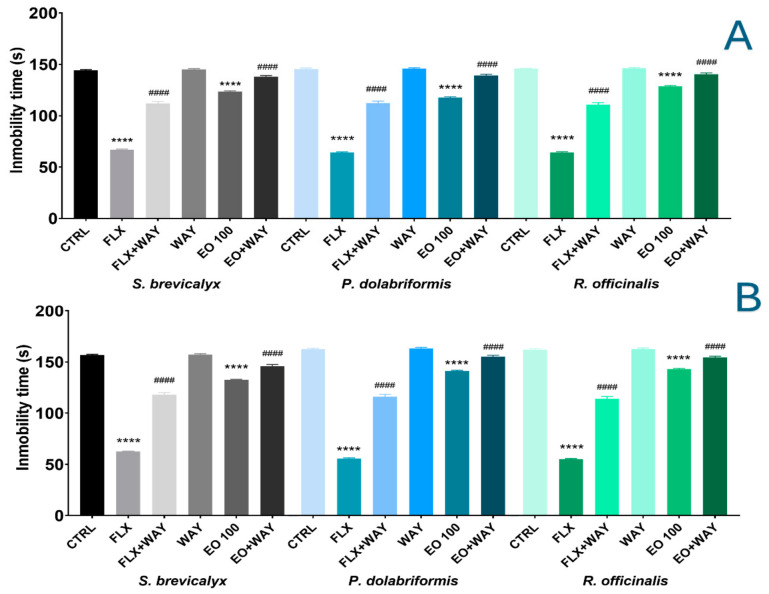
Antagonist-sensitive patterns of the antidepressant-like effects of the three essential oils in the tail suspension test and forced swim test. WAY-100635 was used to assess whether the antidepressant-like behavioral responses to the essential oils of *Satureja brevicalyx*, *Peperomia dolabriformis*, and *Rosmarinus officinalis* were sensitive to 5-HT1A-related pharmacological modulation. (**A**) Immobility time in the tail suspension test. (**B**) Immobility time in the forced swim test. Each essential oil was tested at 100 mg/kg alone and in combination with WAY-100635. CTRL = vehicle-treated control; FLX = fluoxetine 10 mg/kg, i.p.; WAY = WAY-100635; EO100 = essential oil at 100 mg/kg. The bars represent the mean ± SEM; *n* = 8 animals per group. A lower immobility time indicates an antidepressant-like effect. **** *p* < 0.0001 versus CTRL; #### *p* < 0.0001 versus FLX or EO100, as appropriate; ordinary one-way ANOVA followed by Sidak’s selected multiple comparisons test. Antagonist-induced attenuation should be interpreted as evidence compatible with possible pathway participation, not as proof of direct or exclusive 5-HT1A receptor mediation.

Antagonist coadministration assays for antidepressant-like outcomes showed that WAY-100635 attenuated the reduction in immobility induced by both fluoxetine and the essential oils (Figure 7). This pattern indicates that the behavioral responses were sensitive to 5-HT1A-related pharmacological modulation. In all experimental blocks, compared with the control treatment, the fluoxetine treatment significantly reduced the immobility time, whereas the effects of WAY-100635 alone did not differ from those of the control treatment. Compared with fluoxetine alone, coadministration of WAY-100635 with fluoxetine significantly increased immobility, confirming the pharmacological sensitivity of both behavioral paradigms. However, these findings should be interpreted as antagonist-sensitive behavioral evidence compatible with possible 5-HT1A-related pathway participation rather than as proof of direct receptor mediation.

With respect to SBEO, one-way ANOVA revealed significant treatment effects in the tail suspension test (TST) [F(5,42) = 818.4, *p* < 0.0001] and forced swim test (FST) [F(5,42) = 821.0, *p* < 0.0001]. In the TST, fluoxetine reduced immobility from 144.3 s in the CTRL group to 67.10 s (*p* < 0.0001), whereas WAY-100635 alone did not significantly change immobility (145.0 s; *p* = 0.9977 vs. CTRL). WAY-100635 attenuated the fluoxetine effect, increasing immobility to 112.0 s (*p* < 0.0001 vs. FLX). SBEO at 100 mg/kg reduced immobility to 123.6 s (*p* < 0.0001 vs. CTRL), and coadministration with WAY-100635 increased immobility to 138.0 s (*p* < 0.0001 vs. EO100). However, the activity in the EO + WAY group remained significantly lower than that in the CTRL group (*p* = 0.0006), indicating partial rather than complete attenuation. In the FST, SBEO showed the same pattern. Fluoxetine reduced immobility from 156.5 s in the CTRL group to 62.50 s (*p* < 0.0001), whereas WAY-100635 alone had no significant effect (157.0 s; *p* > 0.9999 vs. the CTRL group). WAY-100635 increased immobility in the fluoxetine-treated mice to 118.0 s (*p* < 0.0001 vs. FLX). SBEO reduced immobility to 132.5 s (*p* < 0.0001 vs. CTRL), whereas SBEO + WAY increased immobility to 146.0 s (*p* < 0.0001 vs. EO100). The EO + WAY group remained significantly different from the CTRL group (*p* < 0.0001), again supporting partial attenuation.

For PDEO, significant treatment effects were observed for both TST [F(5,42) = 766.9, *p* < 0.0001] and FST [F(5,42) = 1025, *p* < 0.0001]. In the TST, fluoxetine decreased immobility from 145.8 s in the CTRL group to 64.30 s (*p* < 0.0001), whereas WAY-100635 alone had no significant effect (146.0 s; *p* > 0.9999 vs. the CTRL group). WAY-100635 significantly attenuated fluoxetine activity, increasing immobility to 112.5 s (*p* < 0.0001 vs. FLX). PDEO at 100 mg/kg reduced immobility to 118.0 s (*p* < 0.0001 vs. CTRL), whereas PDEO + WAY increased immobility to 139.5 s (*p* < 0.0001 vs. EO100). The EO + WAY group remained slightly but significantly lower than the CTRL group (*p* = 0.0021), indicating partial attenuation. In the FST, fluoxetine reduced immobility from 162.3 s in the CTRL group to 55.60 s (*p* < 0.0001), and WAY-100635 alone did not alter immobility (163.0 s; *p* = 0.9998 vs. the CTRL group). Fluoxetine + WAY increased immobility to 116.0 s (*p* < 0.0001 vs. FLX). PDEO reduced immobility to 141.1 s (*p* < 0.0001 vs. CTRL), whereas PDEO + WAY increased immobility to 155.0 s (*p* < 0.0001 vs. EO100). Compared with the control group, the EO + WAY group remained significantly different (*p* = 0.0018), which is consistent with the partial attenuation of the PDEO effect.

For ROEO, one-way ANOVA also revealed significant treatment effects in the TST [F(5,42) = 921.2, *p* < 0.0001] and FST [F(5,42) = 1188, *p* < 0.0001]. In the TST, fluoxetine reduced immobility from 145.9 s in the CTRL group to 64.40 s (*p* < 0.0001), whereas the duration of immobility in the WAY-100635 alone group did not differ from that in the CTRL group (146.4 s; *p* > 0.9999). Coadministration of WAY-100635 attenuated the fluoxetine effect, increasing immobility to 111.0 s (*p* < 0.0001 vs. FLX). ROEO at 100 mg/kg reduced immobility to 129.0 s (*p* < 0.0001 vs. CTRL), and ROEO + WAY increased immobility to 140.5 s (*p* < 0.0001 vs. EO100). The EO + WAY group still differed from the CTRL group (*p* = 0.0048), indicating partial attenuation. In the FST, fluoxetine reduced immobility from 161.9 s in the CTRL group to 55.10 s (*p* < 0.0001), whereas WAY-100635 alone had no significant effect (162.5 s; *p* = 0.9999 vs. the CTRL group). Fluoxetine + WAY increased immobility to 114.0 s (*p* < 0.0001 vs. FLX). ROEO reduced immobility to 142.9 s (*p* < 0.0001 vs. CTRL), whereas ROEO + WAY increased immobility to 154.0 s (*p* < 0.0001 vs. EO100). The EO + WAY group remained significantly different from the CTRL group (*p* = 0.0002), supporting partial attenuation.

Overall, WAY-100635 consistently attenuated the antidepressant-like effects of SBEO, PDEO, and ROEO in both the TST and the FST, whereas WAY-100635 alone did not affect immobility compared with the control treatment. The antagonist did not completely abolish the effects of the essential oils, as the EO + WAY groups remained significantly different from the CTRL group across all comparisons. These findings indicate that the antidepressant-like responses to the three oils were sensitive to 5-HT1A-related pharmacological modulation, but they do not fully account for the behavioral effects and should not be interpreted as proof of exclusive serotonergic mediation. Together with the elevated plus-maze antagonist data, these results support a cautious pathway-level interpretation, as summarized in Supplementary Table S9.

### 2.10. Exploratory Serum Corticosterone and Cytokine Profiles

Exploratory serum biomarker analysis was performed in behavior-naïve, nonstress-challenged animals to assess peripheral neuroendocrine–immune correlations after acute essential oil administration. Because EO100 was the effective nonsedative dose selected for the antagonist-coadministration assays, Table 3 summarizes the EO100-versus-vehicle biomarker profile, whereas the complete dose–response dataset for EO25, EO50, and EO100 is provided in Supplementary Table S7. Dunnett-adjusted *p* values were used for within-block comparisons against the corresponding vehicle group, and Benjamini–Hochberg false discovery rate (BH-FDR) q values were calculated across all 54 dose-versus-vehicle biomarker comparisons reported in Supplementary Table S7.

Among the three essential oils, SBEO showed the clearest peripheral biomarker modulation at EO100. SBEO reduced the serum corticosterone levels from 83.97 ± 1.84 to 75.57 ± 0.86 ng/mL, corresponding to a 10.0% decrease that remained significant after BH-FDR correction (Dunnett-adjusted *p* < 0.0001; q = 0.0021). SBEO also reduced the TNF-α levels from 15.67 ± 1.24 to 11.19 ± 0.36 pg/mL (−28.6%; *p* = 0.0008; q = 0.0227) and increased the IL-4 levels from 19.27 ± 0.89 to 23.45 ± 0.90 pg/mL (+21.7%; *p* = 0.0013; q = 0.0236). The levels of IL-6, IL-1β, and IL-10 showed nominal EO100-associated changes, but these changes did not significantly change after BH-FDR correction: the concentration of IL-6 decreased from 32.35 ± 1.36 to 28.65 ± 0.61 pg/mL (−11.5%; *p* = 0.0211; q = 0.1762), the concentration of IL-1β decreased from 30.43 ± 1.13 to 26.72 ± 0.75 pg/mL (−12.2%; *p* = 0.0228; q = 0.1762), and the concentration of IL-10 increased from 25.30 ± 1.09 to 29.20 ± 0.70 pg/mL (+15.4%; *p* = 0.0137; q = 0.1476). In the complete dose–response dataset, an additional FDR-significant reduction in IL-1β was observed for SBEO at the EO50, but this effect was not maintained at the EO100; therefore, this finding was interpreted as a nonmonotonic exploratory finding rather than as part of the main EO100 biomarker signature.

In contrast, PDEO did not produce robust FDR-induced changes in the levels of corticosterone or cytokines at EO100. Corticosterone showed only a small numerical reduction from 83.23 ± 1.61 to 79.53 ± 2.64 ng/mL (−4.4%; *p* = 0.4399; q > 0.9999). Similarly, the levels of IL-6 (−1.6%; *p* = 0.9869; q > 0.9999), TNF-α (−9.1%; *p* = 0.2809; q = 0.9708), IL-1β (−6.0%; *p* = 0.6311; q > 0.9999), IL-10 (+2.9%; *p* = 0.9693; q > 0.9999), and IL-4 (+3.9%; *p* = 0.9700; q > 0.9999) did not exhibit robust EO100-associated modulation.

ROEO also showed no significant changes in serum biomarkers at EO100. Corticosterone decreased numerically from 81.98 ± 1.67 to 76.81 ± 2.62 ng/mL (−6.3%; *p* = 0.1954; q = 0.8118), but this change did not remain statistically significant after multiplicity correction. Similarly, the levels of IL-6 (−2.1%; *p* = 0.9511; q > 0.9999), TNF-α (−14.2%; *p* = 0.2876; q = 0.9708), IL-1β (−8.5%; *p* = 0.3152; q > 0.9999), IL-10 (+4.3%; *p* = 0.9378; q > 0.9999), and IL-4 (+5.3%; *p* = 0.9357; q > 0.9999) did not significantly change in response to EO100.

Overall, the exploratory biomarker profile suggests that SBEO, but not PDEO or ROEO, produced a detectable acute peripheral neuroendocrine–immune signature at the effective nonsedative dose. This profile was characterized by reduced levels of corticosterone and TNF-α, together with increased levels of IL-4, after correction for multiple biomarker comparisons. The absence of robust serum cytokine modulation for PDEO and ROEO indicates that their behavioral effects were not paralleled by strong acute peripheral cytokine changes under the present nonstress-challenged conditions. Therefore, these biomarkers should be interpreted as exploratory peripheral correlates rather than as definitive evidence of systemic anti-inflammatory activity.

## 3. Discussion

The present study provides an integrated chemical, in silico, functional neurobehavioral, antagonist-coadministration, and exploratory biomarker evaluation of the essential oils *of Satureja brevicalyx*, *Peperomia dolabriformis*, and *Rosmarinus officinalis* in acute mouse models related to anxiety- and depression-like behaviors. The study was designed to move beyond isolated behavioral screening by combining GC-MS-based semi-quantitative chemical fingerprinting, chemical-class distribution analysis, molecular docking, preliminary acute oral toxicity assessment, behavioral pharmacology, antagonist-sensitive attenuation assays, and exploratory serum corticosterone and cytokine profiling. This integrated design is relevant because essential oils are multicomponent mixtures whose biological effects may reflect additive, synergistic, or pathway-convergent interactions among volatile constituents rather than a single-target pharmacological action [13]. Nevertheless, the mechanistic interpretation of these findings should remain cautious, as docking, antagonist attenuation, and peripheral biomarkers provide complementary but indirect evidence of possible pathway participation.

The GC-MS profiles indicated that the three essential oils differed markedly in their semi-quantitative chemical fingerprints and annotated chemical class distributions. These differences are important for interpreting their distinct neurobehavioral profiles, although the compound-level assignments should be considered putative GC-MS annotations, and selected constituents, particularly in PDEO, remain tentative because authentic standards and experimentally calculated retention indices were not available. SBEO was characterized by a linalool-rich profile, accompanied by pulegone, menthone, limonene, bicyclogermacrene, β-caryophyllene, γ-terpinene, and isomenthone. This profile is consistent with those of two previous reports describing *S. brevicalyx* essential oil as rich in linalool, menthone, pulegone, and related monoterpenes [15,25]. The predominance of oxygenated monoterpenes and monoterpene ketones is pharmacologically relevant because several compounds in this chemical space have been associated with central nervous system-related behavioral effects, including anxiolytic-like and sedative responses. Linalool, in particular, has shown anxiolytic-like activity in mice without motor impairment, and experimental work suggests that its effects may involve olfactory and GABAergic-related mechanisms [26,27].

In contrast, ROEO displayed a classical rosemary-like monoterpenoid profile dominated by 1,8-cineole/eucalyptol, α-terpineol, limonene, α-pinene, linalool, camphor, β-pinene, bornyl acetate, and camphene. This composition is in agreement with the known chemical variability of rosemary essential oil, in which 1,8-cineole, α-pinene, camphor, verbenone, borneol, and related oxygenated monoterpenes may dominate depending on geography, chemotype, plant material, and extraction conditions [28]. The presence of α-terpineol, linalool, and limonene provides a plausible chemical basis for part of the observed neurobehavioral activity, whereas the high relative abundance of 1,8-cineole may contribute to a profile that was more moderate and less strongly antagonist-sensitive than that observed for SBEO. Previous experimental studies have reported antidepressant- and anxiolytic-like effects of rosemary-derived preparations, including the possible involvement of oxytocinergic and stress-related pathways [18,29].

The PDEO profile was the most chemically distinct. Unlike the two Lamiaceae oils, this oil showed a high relative TIC-area contribution of aromatic/phenylpropanoid-type and sesquiterpenoid constituents, including the tentatively annotated 5-tert-butyl-1,3-benzodioxole, limonene, myristicin, ishwarane, ishwarol B, and elemicin. This is important because the neurobehavioral profile of PDEO cannot be interpreted only through the linalool/oxygenated-monoterpene framework that applies more clearly to SBEO and, to a lesser extent, ROEO. Myristicin and elemicin are phenylpropanoid-type constituents known from other aromatic plants, such as nutmeg, where they contribute to distinctive neuroactive and safety-related profiles [30]. Therefore, the behavioral effects observed for PDEO may reflect a different chemical background and potentially broader pathway compatibility; however, this interpretation remains hypothesis-generating because selected PDEO annotations require confirmation with authentic standards and experimentally calculated retention indices.

The molecular docking results provided an exploratory in silico layer to this comparative interpretation by suggesting differentiated target-compatibility profiles among the major GC-MS-annotated constituents of the three oils. The most favorable docking scores were concentrated mainly around monoaminergic and GABAergic targets, particularly MAO-A, MAO-B, GABA-A-related targets, and 5-HT1A-related targets. This pattern is biologically plausible because GABAergic inhibition, serotonergic regulation, and monoaminergic tone are central to anxiety- and depression-related neuropharmacology [31,32,33,34]. However, docking must be interpreted cautiously because binding-energy estimates do not establish receptor activation, antagonism, allosteric modulation, or enzyme inhibition. Thus, the docking results should be understood as a hypothesis-generating bridge between chemical fingerprints and antagonist coadministration experiments, rather than as mechanistic proof. This distinction is especially important because in silico affinity alone cannot substitute for receptor binding assays, cellular functional assays, enzyme-inhibition assays, electrophysiology, or molecular dynamics/free-energy validation [35].

For SBEO, the docking profile suggested that β-caryophyllene and bicyclogermacrene may contribute to monoamine-related target compatibility, whereas linalool, pulegone, menthone, and related monoterpenes showed predicted compatibility with GABA-A- and monoamine-related targets. This pattern is consistent with, but does not prove, the later observation that SBEO showed the clearest flumazenil-sensitive anxiolytic-like attenuation pattern. β-Caryophyllene is relevant because it has been reported to produce anxiolytic- and antidepressant-like effects in mice and is known as a CB2 receptor agonist, although cannabinoid-related docking was not the dominant profile in the present dataset [36]. For PDEO, the broad predicted docking pattern of ishwarane, ishwarol B, myristicin, and elemicin is compatible with its mixed flumazenil- and WAY-100635-sensitive attenuation pattern; however, direct functional neuropharmacological evidence for ishwarane and ishwarol B remains limited, and these docking-based associations require validation in receptor binding, enzyme inhibition, or cellular signaling assays [37,38,39]. For ROEO, α-terpineol and linalool showed the most favorable predicted profiles among the major annotated constituents, whereas 1,8-cineole, despite being the largest TIC-area constituent, showed a more moderate predicted docking pattern. This may partly explain why ROEO produced significant but comparatively more moderate behavioral effects, although this interpretation remains exploratory.

The preliminary acute oral toxicity results provide a limited safety context for interpreting the acute behavioral experiments. No mortality was observed at doses up to 5000 mg/kg for any of the three essential oils within the 24-h observation window, and only mild-to-moderate transient clinical signs were observed at high doses. These findings support the feasibility of the acute behavioral dose range used in this study, but they should not be interpreted as evidence of complete toxicological safety or as a full OECD-compliant acute toxicity assessment. Acute lethality screening is fundamentally different from subacute, chronic, reproductive, hepatic, renal, or genotoxicity testing [40]. This distinction is particularly relevant for essential oils, because some constituents may exhibit dose-dependent or organ-specific toxicity even when no acute mortality is observed; for example, pulegone-containing oils require caution at high exposure levels because pulegone has been associated with hepatotoxicity in toxicological contexts [41]. Therefore, the preliminary acute toxicity data support the selected doses for the acute behavioral phase, but they do not establish long-term safety, dietary suitability, or translational applicability.

From an application perspective, the oral doses used in the present study should be considered pharmacological preclinical doses rather than dietary exposure levels. Using standard body-surface-area scaling, the mouse doses of 25, 50, and 100 mg/kg correspond approximately to human-equivalent doses of 2.0, 4.1, and 8.1 mg/kg, respectively, equivalent to approximately 120, 240, and 490 mg/day for a 60-kg adult [42]. These values refer to whole essential oil exposure and are likely higher than amounts typically achievable or sensorially acceptable through dietary use as spices, flavoring materials, or food ingredients. Therefore, the current findings should not be interpreted as evidence supporting direct use as functional food ingredients, food additives, or dietary supplements. Any such application would require repeated-dose toxicity studies, constituent-specific risk assessment, regulatory evaluation, exposure estimation, sensory acceptability assessment, and batch-to-batch chemical standardization [43].

The behavioral results demonstrate that all three essential oils produced anxiolytic-like effects, but with different magnitudes and consistencies across the paradigms. SBEO showed the most robust and coherent profile, with increased open-arm exploration in the elevated plus maze, increased time in the illuminated compartment in the light–dark box, and reduced marble burying. These convergent effects across complementary anxiety-related paradigms reduce the probability that the findings reflect a test-specific artifact. These results are also consistent with the broader essential-oil literature, which reports anxiolytic and neurobehavioral effects for several *Lamiaceae* species, including the genus *Satureja* [8,16,44,45]. The chemical profile of *S. brevicalyx* provides a plausible basis for this behavior. The oil was rich in linalool, pulegone, menthone, limonene, β-caryophyllene, and bicyclogermacrene, suggesting a mixed monoterpenoid–sesquiterpenoid profile. Linalool is among the best-studied anxiolytic volatile compounds and has been shown to produce anxiolytic-like effects in mice, including in elevated plus maze and light–dark box paradigms. Experimental evidence also suggests that linalool and its metabolites can interact with GABAergic mechanisms, including the modulation of GABA-A receptor-related signaling [26,46]. Additionally, β-caryophyllene may also contribute to the neurobehavioral profile, given its reported anxiolytic- and antidepressant-like effects and its pharmacological relevance as a CB2 receptor agonist [36].

The anxiolytic-like effects of PDEO were especially strong in the elevated plus-maze test and became more evident at 50 and 100 mg/kg in the light–dark box and marble burying tests. This pattern suggests that PDEO may exhibit a threshold-dependent effect in more ethologically defensive paradigms, whereas it produces a clearer response in open-arm exploration. Given the limited neurobehavioral literature on PDEO, these results are novel and support this oil as an emerging candidate for deeper neuropharmacological investigation. ROEO also produced significant anxiolytic-like effects, but these effects were generally more moderate and more evident at 50 and 100 mg/kg. This finding is consistent with the findings of previous studies showing the anxiolytic-like activity of rosemary preparations, but also suggests that the magnitude of the effects of rosemary oil may depend strongly on the chemotype, route of administration, dosing, and experimental model [19,20].

The antidepressant-like results were also consistent across both the tail suspension test and forced swim test. All three oils reduced immobility time in a dose-dependent manner, supporting antidepressant-like activity. These findings are strengthened by the results of the open field test, in which none of the essential oil-treated groups exhibited locomotor suppression. This is important because reduced or increased immobility in the TST and FST can be confounded by nonspecific changes in motor activity. In this study, the positive sedating control, diazepam at 3.5 mg/kg, markedly reduced crossing, whereas the oils did not. Therefore, the reductions in immobility are unlikely to be explained by sedation, motor impairment, or general behavioral suppression. These effects may be partially explained by monoaminergic mechanisms suggested by the docking profile. Linalool and β-pinene have been reported to exert antidepressant-like effects through monoaminergic pathways, whereas β-caryophyllene has been linked to behavioral changes relevant to anxiety and depression in preclinical models [36,47]. The strong predicted interactions with MAO-A and MAO-B observed for several dominant constituents are therefore consistent with the reductions in immobility observed in the TST and FST. Nevertheless, because MAO docking does not demonstrate enzymatic inhibition, the present results should be framed as consistent with monoaminergic involvement rather than as proof of MAO inhibition.

The antagonist-coadministration experiments provided pharmacological evidence of antagonist-sensitive behavioral attenuation patterns, but these results should not be interpreted as definitive proof of receptor mediation. During the anxiolytic-like phase, flumazenil markedly attenuated the effect of diazepam, confirming that the elevated plus maze protocol was sensitive to benzodiazepine-site pharmacological modulation. SBEO showed the clearest flumazenil-sensitive attenuation pattern, especially for open-arm time, where the EO100 + flumazenil group no longer differed significantly from the vehicle group. This pattern is compatible with the possible participation of GABA-A/benzodiazepine-related pathways in the anxiolytic-like response to SBEO, but it does not establish direct receptor binding, functional GABA-A modulation, or exclusive benzodiazepine-site mediation. This cautious interpretation is consistent with previous studies showing that selected essential oils and volatile constituents, such as *Cymbopogon citratus* essential oil and citral, can produce anxiolytic-like effects that are sensitive to GABA-A/benzodiazepine-related or broader GABAergic modulation, as well as with reviews identifying GABAergic signaling as a relevant target for neuroactive essential oil constituents [48,49].

In contrast, the anxiolytic-like response to PDEO was attenuated by both flumazenil and WAY-100635. This finding suggests a mixed, antagonist-sensitive pattern compatible with the possible participation of the GABA-A/benzodiazepine- and 5-HT1A-related pathways. This interpretation is supported by, but not proven by, the broad predicted docking profile of PDEO, especially the favorable predicted interactions of ishwarane and ishwarol B with GABA-A, GABA-A α1, MAO-A/MAO-B, SERT, and 5-HT1A-related targets. Because direct functional neuropharmacological evidence for ishwarane and ishwarol B remains limited, these docking-based associations should be interpreted as pathway-level hypotheses that require validation in receptor binding, enzyme inhibition, cellular signaling, or electrophysiological assays. The WAY-100635-sensitive component is also consistent with previous reports that essential oils such as *Citrus aurantium*, lavender, *Piper nigrum*, and *Pelargonium roseum* may exert anxiety- or depression-related effects through 5-HT1A or broader serotonergic transmission [50,51,52,53].

The ROEO profile was more moderate and appeared more clearly sensitive to flumazenil than to WAY-100635 in the anxiolytic-like phase. At the open-arm time endpoint, WAY-100635 did not significantly attenuate the EO100 response, whereas flumazenil did. This pattern suggests that under the present acute experimental conditions, the anxiolytic-like response to ROEO was more consistently flumazenil-sensitive than WAY-100635-sensitive. However, because WAY-100635 partially affected open-arm entries, a contribution of the 5-HT1A-related pathways cannot be fully excluded. This asymmetric antagonist-sensitive profile is consistent with the multicomponent nature of rosemary oil, and with the possibility that different elevated plus maze endpoints may differ in their sensitivity to pharmacological modulation.

The antagonist-coadministration assays for antidepressant-like outcomes revealed a more convergent pattern. WAY-100635 attenuated the EO100-induced reduction in immobility for all three essential oils in both the TST and FST, whereas the effect of WAY-100635 alone did not differ from that of the vehicle. These results indicate that the antidepressant-like responses to SBEO, PDEO, and ROEO were sensitive to 5-HT1A-related pharmacological modulation, consistent with previous reports linking serotonergic pathways to the behavioral effects of neuroactive essential oils [52,53]. Importantly, the attenuation was partial rather than complete, as the EO100 + WAY groups generally remained significantly different from the vehicle group. This is biologically plausible because essential oils are multicomponent mixtures that may exert biological effects through several interacting constituents and pathway-convergent mechanisms [13]. Therefore, the antagonist data support possible pathway participation but do not prove direct 5-HT1A receptor mediation or exclude the contribution of additional monoaminergic, GABAergic, inflammatory, endocrine, or other neurobehavioral pathways.

The exploratory biomarker results add a peripheral neuroendocrine–immune dimension to the study, but they should be interpreted with appropriate caution. SBEO was the only oil that produced FDR-significant EO100-associated changes, characterized by reduced corticosterone and TNF-α levels and increased IL-4 levels. This profile is compatible with a modest acute peripheral endocrine–immune shift after SBEO administration. However, because biomarkers were measured in behavior-naïve, nonstress-challenged animals after a single acute exposure, these findings should not be interpreted as definitive mechanistic mediators of the behavioral effects. Regulation of the HPA axis is central to stress adaptation, and dysregulated glucocorticoid signaling is strongly implicated in stress-related affective disturbances [54]. In addition, inflammatory mediators such as IL-6, TNF-α, and IL-1β are frequently discussed in relation to depression and stress-related psychopathology, although the strength and consistency of these associations vary across biomarkers, populations, and clinical subtypes [55,56].

The absence of robust FDR-significant modulation of biomarkers for PDEO and ROEO does not contradict their behavioral or antagonist-sensitive effects. Central behavioral modulation can occur without detectable acute changes in peripheral serum cytokines, especially after a single dose in animals not exposed to chronic stress or an inflammatory challenge. Moreover, peripheral cytokines and corticosterone are highly time sensitive and may depend on the circadian phase, sampling time, strain, sex, prior stress exposure, and the presence or absence of a pathological challenge. Previous work in mice has shown that serum corticosterone dynamics vary according to circadian rhythm and stressor exposure, whereas peripheral cytokine findings in depression-related research are heterogeneous across biomarkers, populations, and experimental contexts [57,58]. Therefore, the biomarker findings should be interpreted as exploratory peripheral correlates rather than definitive mechanistic mediators. This interpretation is also consistent with the study design, which intentionally avoided behavioral test exposure before blood sampling to prevent forced swim- or stress-induced endocrine confounding.

Taken together, the findings support differentiated but convergent functional neurobehavioral profiles among the three essential oils, rather than a single shared pathway profile. SBEO combined a linalool/monoterpene-rich semi-quantitative chemical fingerprint, a consistent flumazenil-sensitive anxiolytic-like attenuation pattern, WAY-100635-sensitive antidepressant-like attenuation, and the clearest exploratory peripheral endocrine–immune modulation. PDEO showed a chemically distinct phenylpropanoid/aromatic compound-rich profile, the broadest predicted multitarget docking pattern, and mixed flumazenil- and WAY-100635-sensitive anxiolytic-like attenuation, but no robust acute peripheral biomarker modulation. ROEO showed a rosemary-like monoterpenoid profile, significant but more moderate behavioral effects, a mainly flumazenil-sensitive anxiolytic-like attenuation pattern, and WAY-100635-sensitive antidepressant-like attenuation. These differences support the value of a comparative design because the three oils produced broadly similar behavioral outcomes but did not share identical chemical fingerprints, docking profiles, antagonist-sensitive patterns, or biomarker signatures.

Several limitations should be acknowledged. First, the study used acute administration, whereas anxiety and depression are chronic or recurrent clinical conditions; therefore, sustained efficacy after repeated treatment cannot be inferred from the present data. Second, the TST and FST are pharmacological screening paradigms related to stress coping and antidepressant sensitivity, but they should not be interpreted as complete models of human depression. Contemporary discussions emphasize that immobility in the FST is complex and should be interpreted cautiously rather than as a direct depressive-like state [59]. Third, each essential oil was evaluated within its own independent experimental block with its corresponding control and reference drug; therefore, between-oil comparisons should be interpreted descriptively rather than as direct statistical rankings. Fourth, the GC-MS data were based on semi-quantitative TIC-area profiles and putative annotations without authentic standards, experimentally calculated retention indices, GC-FID response-factor correction, or internal-standard-based quantification. This limitation is especially relevant for selected PDEO constituents, including 5-tert-butyl-1,3-benzodioxole, ishwarane, and ishwarol B, which require confirmation in future targeted chemical analyses. Fifth, the study used a single batch of each essential oil; therefore, the influence of plant origin, season, phenological stage, environmental conditions, and extraction variability on chemical composition and biological activity could not be assessed. Sixth, the docking results were not validated by receptor binding, electrophysiological, enzyme inhibition, cellular functional assays, molecular dynamics simulations, or free-energy refinement. Seventh, antagonist-coadministration assays indicated antagonist-sensitive attenuation patterns but not direct or exclusive receptor mediation, especially because attenuation was generally partial. In addition, antagonist-alone groups were not included in the elevated plus maze antagonist-coadministration phase; therefore, the anxiolytic-like antagonist findings should be interpreted specifically as attenuation of EO100-induced responses rather than as a complete pharmacological characterization of antagonist effects per se. Eighth, only male BALB/c mice were used, limiting generalization across sex, strain, and hormonal status. Using males reduced biological variability in this initial comparative screening, but future studies should include both sexes and consider estrous-cycle-related factors where appropriate. Ninth, serum biomarkers were measured in behavior-naïve animals without chronic stress or inflammatory challenge; therefore, direct mediation between behavioral effects and changes in the HPA axis or cytokines could not be established. Finally, the preliminary toxicity assessment was limited to a 24-h acute observation window, and whole essential oils were tested rather than isolated constituents, standardized fractions, or reconstructed mixtures; therefore, the study does not establish subacute or chronic safety, organ-specific safety, dietary suitability, or the contribution of individual compounds to the observed effects.

Future studies should evaluate these oils in repeated-treatment and disease-relevant models, including chronic unpredictable mild stress, repeated restraint stress, social defeat stress, or inflammation-associated affective models. Such designs would clarify whether the acute effects observed here translate into sustained behavioral improvement under pathological stress conditions. Functional validation should include GABA-A receptor modulation assays, 5-HT1A receptor binding or signaling assays, MAO-A/MAO-B enzyme inhibition, SERT-related assays, and electrophysiological approaches where feasible. Additional in silico validation using molecular dynamics simulations, free-energy refinement, and benchmarking against reference ligands would also strengthen the interpretation of target compatibility. Chemically, future work should include experimentally calculated retention indices using n-alkane series, authentic standards for key constituents, GC-FID or internal-standard-based quantification, and batch-to-batch analyses to evaluate chemical reproducibility. Fractionation, isolated-compound testing, and recombination experiments are also necessary to determine whether the effects depend on dominant constituents, minor compounds, or synergistic interactions. Pharmacokinetic and brain distribution studies will be essential for determining whether key constituents reach central targets after oral administration. Finally, studies on subacute and chronic toxicity, including studies on hepatic, renal, hematological, and histopathological endpoints, are needed before any translational or application-oriented inference can be made.

## 4. Materials and Methods

### 4.1. Plant Material Collection and Essential Oil Isolation

Plant materials were collected in Peru between January and February 2025 from their respective habitats. Leaves of *Satureja brevicalyx* and *Peperomia dolabriformis* were obtained from wild populations; *S. brevicalyx* was collected in Huamanguilla, Acocro district, Ayacucho (3255 m above sea level; coordinates: 13.208102° S, 74.025510° W), while *P. dolabriformis* was obtained from Cerro Campana in Trujillo Province, La Libertad (284 m above sea level; coordinates: 7.9812457° S, 79.1059127° W). Concurrently, leaves of *Rosmarinus officinalis* were collected from individuals cultivated in Otuzco, La Libertad (2640 m above sea level; coordinates: 7.909035° S, 78.516802° W). All species were taxonomically identified, and voucher specimens were prepared and deposited at the Herbarium Truxillense (HUT) of the National University of Trujillo under the accession codes HUT 58165, HUT 59458, and HUT 59876.

The essential oils were isolated from the leaves by steam distillation for 1 h using a Figmay laboratory-scale extraction unit (Figmay S.R.L., Córdoba, Argentina). The recovered oils were dried over anhydrous sodium sulfate (Na_2_SO_4_) and stored in amber glass vials at 4 °C until chemical and biological analyses.

Essential oil yields were calculated relative to the fresh leaf material used for distillation and expressed as a *v*/*w* percentage. The yields were 0.91% for *Satureja brevicalyx* essential oil, 0.29% for *Peperomia dolabriformis* essential oil, and 0.54% for *Rosmarinus officinalis* essential oil.

For each species, a single essential-oil batch obtained from one plant collection and extraction was used for GC-MS profiling and for all subsequent in silico, toxicity, behavioral, and biomarker experiments. This strategy ensured that all biological assays were linked to the same chemically profiled oil batch. However, independent batch-to-batch replication was not performed; therefore, the GC-MS results and biological findings should be interpreted as representative of the analyzed batches rather than as evidence of chemotype-wide or batch-independent reproducibility.

### 4.2. Essential Oil Chemical Characterization by GC-MS

The essential oils from *Satureja brevicalyx*, *Peperomia dolabriformis*, and *Rosmarinus officinalis* were chemically profiled by gas chromatography-mass spectrometry (GC-MS) using a Shimadzu GC-2010 Plus gas chromatograph coupled to a QP2010 Ultra mass spectrometer (Shimadzu Corporation, Kyoto, Japan). Before analysis, each essential oil was dried over anhydrous sodium sulfate (J.T. Baker, Phillipsburg, NJ, USA), filtered through a 0.45 μm syringe filter, and diluted by transferring 0.20 mL of the filtered oil into a 10 mL volumetric flask and bringing it to volume with n-hexane (J.T. Baker, Phillipsburg, NJ, USA).

Chromatographic separation was performed on a Restek Rtx-5MS capillary column (30 m × 0.25 mm i.d., 0.25 μm film thickness; Restek Corporation, Bellefonte, PA, USA). Helium (grade 5.0) was used as the carrier gas under linear velocity control (32.4 cm·s^−1^), corresponding to a column flow rate of 0.80 mL·min^−1^. The injector was operated in split mode (20:1) at 220 °C, and 1.0 μL of each diluted sample was manually injected. The oven temperature program was set as follows: 50 °C for 10 min, increased at 3 °C·min^−1^ to 150 °C and held for 20 min, then increased at 10 °C·min^−1^ to 300 °C and held for 5 min, for a total run time of 83.33 min. The mass spectrometer was operated in electron ionization (EI) mode at 70 eV, with the ion source maintained at 250 °C and the interface at 290 °C. A solvent cutoff time of 2 min was applied, and data acquisition started at 3 min in full-scan mode over an *m*/*z* range of 20–500.

Constituents were annotated by automated spectral matching against the NIST 2014 library implemented in the Shimadzu GCMSsolution software 2.53 (Shimadzu Corporation, Kyoto, Japan), followed by manual inspection of mass spectra when needed. Compound assignments were further evaluated by comparison with reference retention index (RI) values reported for DB-5/HP-5/Rtx-5MS-type columns in the NIST Chemistry WebBook, Babushok et al., Adams’ database, and other literature sources. Because a homologous n-alkane series was not acquired under the same chromatographic conditions, experimentally calculated retention indices were not available. Therefore, the RI values reported in the main and Supplementary Tables are presented as indicative literature/reference RI values and should not be interpreted as definitive experimental RI confirmation.

Major constituents with high EI-MS spectral similarity and RI-compatible elution behavior were retained as putative GC-MS annotations. Compounds with RI/RT uncertainty, broader library assignments, or insufficient orthogonal confirmation were explicitly marked as tentative in the composition tables. This approach is particularly relevant for selected *Peperomia dolabriformis* constituents, including the largest TIC-area peak annotated as 5-tert-butyl-1,3-benzodioxole, which should be considered tentative until confirmed with an authentic standard and experimentally calculated retention index.

Relative abundances were calculated by normalization of total ion current (TIC) peak areas without response-factor correction. No authentic standards, GC-FID profile, or internal-standard-based quantification were used. Accordingly, the reported percentages should be interpreted as semi-quantitative relative TIC-area abundances rather than absolute concentrations or response-corrected quantitative values. Representative GC-MS total ion chromatograms and expanded chromatographic regions for the three essential oils are provided in Supplementary Figures S1–S9, and the complete composition tables are provided in Supplementary Tables S1–S3.

### 4.3. Molecular Docking Analysis

All annotated GC-MS constituents from each essential oil, including putatively annotated and tentative compounds, were subjected to in silico molecular docking analysis against a panel of molecular targets associated with anxiety-, depression-, sleep-, and stress-related pathways. For clarity and biological interpretability, the main heatmap displays only the major GC-MS-annotated constituents of each essential oil, defined as compounds with relative TIC-area abundances ≥5% in the corresponding GC-MS profile. The complete ligand–target docking matrix, including all GC-MS-annotated constituents, is provided in Supplementary Tables S4–S6, whereas the GC-MS annotation tables for each essential oil are provided in Supplementary Tables S1–S3.

The selected molecular targets included GABA-A, 5-HT1A, CB1, CB2, SERT, MAO-A, MAO-B, GABA-A α1, MT1, MT2, OX2R, CRHR1, GR, and MR. These targets were grouped according to their relevance to the anxiety-, depression-, sleep-, and stress-related neuropharmacological pathways. The selection of GABAergic and serotonergic targets was also supported by previous pharmacological studies showing involvement of the GABA-A/benzodiazepine and 5-HT1A receptor-mediated pathways in the behavioral effects of essential oils and related volatile compounds [50,51,52,60].

Three-dimensional structures of the ligands were retrieved from PubChem and energy-minimized using the MMFF94 force field in Open Babel [61]. Protein structures were obtained from the Protein Data Bank or curated structural databases when experimentally resolved structures were unavailable [62]. Receptor structures were prepared by removing crystallographic water molecules, adding polar hydrogens, assigning Gasteiger charges, and defining the docking grid around the orthosteric or allosteric binding site according to the cocrystallized ligand or reported active-site residues. Ligand and receptor preparation was performed using AutoDockTools 1.5.7 (The Scripps Research Institute, La Jolla, CA, USA) [63]. Docking simulations were performed using AutoDock Vina 1.2.5 (The Scripps Research Institute, La Jolla, CA, USA) [64].

For each ligand–target pair, docking output was expressed as the predicted binding energy score (ΔG, kcal/mol), with more negative values indicating more favorable predicted scores within the docking model. These values were used to compare exploratory ligand–target compatibility patterns and should not be interpreted as experimentally measured binding affinities, receptor activation or inhibition, enzyme inhibition, or pharmacological efficacy. Reference ligands were included, when available, as contextual comparators for each target class, including diazepam for the GABA-A/benzodiazepine site, 8-OH-DPAT or serotonin-related ligands for 5-HT1A, selective monoaminergic ligands for SERT and MAO targets, cannabinoid receptor ligands for CB1/CB2, melatonin for MT1/MT2, orexin-related ligands for OX2R, and endogenous or pharmacological ligands for CRHR1, GR, and MR. These reference ligands were used for contextual interpretation and were not intended to constitute a complete docking benchmarking or enrichment-validation procedure.

The docking matrix was organized according to functional target categories: anxiety, depression, sleep, and stress. Predicted docking scores were visualized as a heatmap to compare the target-compatibility profiles of the major annotated constituents across the three essential oils. Molecular interactions, including hydrogen bonding, hydrophobic interactions, π–π interactions, and other relevant ligand–receptor contacts, were visualized using BIOVIA Discovery Studio Visualizer v21 (Dassault Systèmes, San Diego, CA, USA).

Docking results were interpreted as hypothesis-generating in silico evidence rather than as confirmatory proof of pharmacological activity. Based on the docking profile and the biological relevance of the targets, the in vivo antagonist phase focused on the GABA-A/benzodiazepine and 5-HT1A-related pathways using flumazenil and WAY-100635, respectively. Docking results for MAO-A, MAO-B, SERT, CB1, CB2, MT1, MT2, OX2R, CRHR1, GR, and MR were considered exploratory information to support the generation of chemical–neurobehavioral hypotheses for future experimental validation.

### 4.4. Experimental Animals and Ethical Statement

Male BALB/c mice weighing 25–30 g and aged 8–10 weeks were obtained from the Bioterium of Universidad Peruana Cayetano Heredia (Lima, Peru). The animals were housed in groups of four per polycarbonate cage under controlled environmental conditions: temperature 22–25 °C, relative humidity 55–65%, and a 12 h light/dark cycle, with lights on at 07:00 h. Standard rodent chow and filtered water were provided ad libitum. The mice were allowed to acclimate for at least 7 days before any experimental procedures were performed. Behavioral assays were conducted between 09:00 and 15:00 h in a dedicated behavioral testing room under standardized low illumination and minimal ambient noise. All behavioral scoring was performed by a trained observer who was blinded to the treatment allocation. The inclusion criteria were healthy male BALB/c mice within the predefined age range and body weight range, with a normal gross appearance and no signs of illness or abnormal behavior during acclimatization. The exclusion criteria, established a priori, included illness, injury, abnormal baseline behavior, technical failure during drug administration, or inability to complete a behavioral test for reasons unrelated to treatment. No animals or data points were excluded after randomization unless they met these predefined criteria.

The study was conducted in accordance with the Guide for the Care and Use of Laboratory Animals, ARRIVE 2.0 guidelines, and institutional regulations for the ethical use of laboratory animals [65,66]. The protocol was reviewed and approved by the Ethics Committee of the Facultad de Farmacia y Bioquímica, Universidad Nacional de Trujillo (Approval No. 010-2024/CE-FFBB-UNT).

### 4.5. Experimental Design and Treatment Allocation

The in vivo study was organized into four complementary experimental phases: (i) preliminary acute oral toxicity screening; (ii) dose–response behavioral screening; (iii) spontaneous locomotor activity assessment; and (iv) antagonist-coadministration assays to evaluate antagonist-sensitive behavioral attenuation patterns. In the dose–response phase, three oral doses of each essential oil (25, 50, and 100 mg/kg, p.o.) were compared with the corresponding vehicle-treated control and positive control. Diazepam was used as the positive control for the anxiety-related paradigms, whereas fluoxetine was used as the positive control for the depression-related paradigms. Independent cohorts were used for anxiety-related and depression-related behavioral domains to minimize cross-domain carryover effects. The dose range was selected on the basis of doses commonly employed in preclinical studies with essential oils and natural products in anxiety- and depression-related behavioral paradigms [52,53,67].

The anxiety-related behavioral battery included the elevated plus maze test, light–dark box test, and marble burying test. The depression-related behavioral battery included the tail suspension test and forced swim test. Spontaneous locomotor activity was evaluated in an independent open field experiment to determine whether the behavioral effects of the essential oils were associated with nonspecific motor impairment or sedative-like activity. Diazepam at 3.5 mg/kg was included only in this open field experiment as a sedative motor-control condition.

For the antagonist coadministration assays, the highest effective, nonsedative dose, EO100, was selected because it produced significant behavioral effects without reducing spontaneous locomotor activity. Flumazenil was used to assess whether anxiolytic-like responses were sensitive to GABA-A/benzodiazepine-related pharmacological modulation, whereas WAY-100635 was used to assess whether anxiolytic-like and antidepressant-like responses were sensitive to 5-HT1A-related pharmacological modulation [50,51,52]. These assays were designed to evaluate antagonist-sensitive attenuation patterns and were not intended to prove direct receptor binding, receptor activation, or inhibition, or exclusive pathway mediation.

Independent groups of eight mice per treatment condition were used for the behavioral and antagonist-coadministration experiments. The sample size was determined a priori using G*Power 3.1.9.7 (Heinrich Heine University Düsseldorf, Düsseldorf, Germany), with α = 0.05, power = 0.80, and effect size f = 0.40, which is consistent with the group sizes used in analogous neurobehavioral pharmacology studies [52,53,68]. The mice were randomly assigned to treatment groups using a computer-generated randomization sequence. Behavioral scoring was performed by an observer who was blinded to the treatment allocation.

A separate behavior-naïve subcohort was used for exploratory serum corticosterone and cytokine profiling. These animals were not subjected to behavioral testing to avoid acute behavioral test-induced alterations in endocrine and immune markers [69].

### 4.6. Drug Administration and Treatment Timing

Each essential oil was freshly suspended immediately before administration in a vehicle consisting of propylene glycol (Merck Millipore, Darmstadt, Germany): Tween 80 (Merck Millipore, Darmstadt, Germany): 0.9% saline (4:1:5, *v*:*v*:*v*) and administered by oral gavage at 10 mL/kg body weight. Diazepam (Sigma-Aldrich, St. Louis, MO, USA) (1 mg/kg, i.p.) was used as the positive anxiolytic control in the anxiety-related behavioral battery and in the anxiolytic-like antagonist-coadministration assays. Fluoxetine hydrochloride (Sigma-Aldrich, St. Louis, MO, USA) (10 mg/kg, i.p.) was used as the positive antidepressant control in the depression-related behavioral battery and in the antidepressant-like antagonist-coadministration assays. Diazepam at 3.5 mg/kg, i.p., was used only as the sedative motor-control condition in the open field test.

Flumazenil (Sigma-Aldrich, St. Louis, MO, USA) (2.5 mg/kg, i.p.), a competitive antagonist of the benzodiazepine binding site on the GABA-A receptor complex, was used to assess whether anxiolytic-like responses were sensitive to GABA-A/benzodiazepine-related pharmacological modulation. WAY-100635 maleate (Tocris Bioscience, Bristol, UK) (0.1 mg/kg, i.p.), a selective 5-HT1A receptor antagonist, was used to assess whether anxiolytic-like and antidepressant-like responses were sensitive to 5-HT1A-related pharmacological modulation. These antagonist-coadministration assays were interpreted as pathway-level pharmacological attenuation experiments rather than as direct receptor-binding or receptor-function assays.

In the dose–response phase, essential oils or vehicle were administered orally 60 min before behavioral testing, whereas diazepam and fluoxetine were administered intraperitoneally 30 min before testing. In the antagonist-coadministration phase, flumazenil or WAY-100635 was administered 15 min before the corresponding essential oil or reference drug. Behavioral testing was performed 60 min after essential oil administration or 30 min after diazepam or fluoxetine administration, as appropriate. All solutions were freshly prepared on the day of testing.

### 4.7. Preliminary Acute Oral Toxicity Assessment

A preliminary acute oral toxicity assessment was conducted independently for each essential oil using a modified Lorke approach [70], in accordance with the upper-dose-limit principles of OECD Test Guideline No. 423 [71]. In phase 1, three groups of three mice each received 10, 100, or 1000 mg/kg of the corresponding essential oil by oral gavage. In phase 2, three additional groups of three mice each received 1600, 2900, or 5000 mg/kg orally. Thus, 18 mice were used per essential oil, for a total of 54 mice across the three assays. The animals were observed continuously during the first 4 h and again at 24 h for mortality and clinical signs of acute toxicity, including piloerection, tremors, diarrhea, hypoactivity, reduced grooming, ataxia, respiratory distress, convulsions, abnormal posture, and changes in spontaneous behavior. Because no mortality was observed at the highest tested dose, the oral LD50 was estimated to be greater than 5000 mg/kg under the conditions of this preliminary 24-h acute toxicity assessment [70,72].

### 4.8. Anxiety-Related Behavioral Assessment

Anxiety-related behavior was evaluated using the elevated plus maze and light–dark box as complementary paradigms of anxiety-like exploratory behavior, whereas the marble burying test was included as an additional measure related to defensive digging and anxiety-/repetitive-like behavior [73,74,75]. Tests were conducted in the following order: elevated plus maze, light–dark box, and marble burying tests, with a minimum interval of 24 h between tests and redosing before each session.

#### 4.8.1. Elevated Plus Maze

The elevated plus maze test was performed according to the methods of Lister [74], with minor adaptations. The apparatus consisted of two open arms (30 × 10 cm), two enclosed arms (30 × 10 × 25 cm), and a central platform (10 × 10 cm) elevated 40 cm above the floor. The experimental setup was arranged in a room with low illumination and minimal ambient noise. Each mouse was placed individually on the central platform facing one of the open arms and allowed to explore freely for 5 min. Test sessions were video-recorded for subsequent behavioral scoring by a blinded observer. The primary outcomes were the percentage of open-arm entries, calculated as open-arm entries/total arm entries × 100, and the percentage of time spent in the open arms, calculated as open-arm time/total arm time × 100. Increased open-arm exploration was interpreted as an anxiolytic-like response. The apparatus was cleaned with 10% ethanol between the animals to minimize olfactory cues.

#### 4.8.2. Light–Dark Box Test

The light–dark box test was conducted according to Crawley and Goodwin [73]. The apparatus consisted of a rectangular box (45 × 27 × 27 cm) divided into two compartments: an illuminated compartment (27 × 27 × 27 cm) and a dark compartment (18 × 27 × 27 cm), connected by a 7.5 × 7.5 cm opening. Each mouse was placed in the center of the illuminated compartment and observed for 5 min. The primary outcome was the time spent in the illuminated compartment, expressed as a percentage of the total session duration. Increased time in the illuminated compartment was interpreted as an anxiolytic-like effect. The apparatus was cleaned between the animals to minimize olfactory cues.

#### 4.8.3. Marble Burying Test

The marble burying test was performed as a complementary anxiety/repetitive behavior paradigm [75]. Each mouse was placed individually in a polycarbonate cage measuring 42 × 27 × 15 cm, which contained a 5-cm-deep layer of sawdust. Twenty clean glass marbles, each 1.5 cm in diameter, were evenly distributed on the bedding surface in four rows. After 30 min, the number of buried marbles was counted by a blinded observer. A marble was considered buried when at least two-thirds of its surface was covered by bedding. A reduction in the number of buried marbles was interpreted as a decrease in defensive digging behavior.

#### 4.8.4. Antagonist-Coadministration Assessment of Anxiolytic-like Activity

For the antagonist-coadministration assessment of anxiolytic-like activity, the elevated plus maze was selected because it yielded robust, comparable anxiolytic-like responses across the three essential oils during the dose–response phase. Each essential oil was tested at 100 mg/kg alone and in combination with flumazenil or WAY-100635 within the same experimental block. Diazepam and diazepam plus flumazenil were included as pharmacological validation controls for benzodiazepine-site sensitivity of the assay. The primary outcomes were the percentage of open-arm entries and the percentage of time spent in open arms. Attenuation of the EO100-induced response by flumazenil was interpreted as evidence compatible with possible GABA-A/benzodiazepine-related pathway participation, whereas attenuation by WAY-100635 was interpreted as evidence compatible with possible 5-HT1A-related pathway participation [50,51,52]. These interpretations were considered pathway-level pharmacological inferences and not proof of direct receptor mediation or exclusive pathway dependence.

### 4.9. Depression-Related Behavioral Assessment

Depression-related behavior was assessed using the tail suspension test and forced swim test in an independent cohort. The tail suspension test was performed first. The mice were then allowed at least 24 h before the forced swim preswim session, and the forced swim test session was conducted 24 h after the preswim session. Finally, the forced swim test was conducted because it is the most physically stressful procedure and may influence subsequent behavioral outcomes. The animals were redosed before each behavioral test session according to the treatment timing described above [76,77].

#### 4.9.1. Tail Suspension Test

The tail suspension test was performed according to Steru et al. [78], with minor adaptations. The mice were suspended 58 cm above the floor by adhesive tape placed approximately 1 cm from the distal end of the tail. Each session lasted 6 min, and immobility time was recorded during the last 5 min. Immobility was defined as the absence of escape-oriented movements. A reduction in immobility time was interpreted as an antidepressant-like effect.

#### 4.9.2. Forced Swim Test

The forced swim test was conducted according to Porsolt et al. [79], with minor modifications. Twenty-four hours before the test session, the mice underwent a 15-min preswim session to standardize prior to swim exposure. This preswim session was scheduled after the tail suspension test to avoid influencing earlier depression-related outcomes. On the test day, the mice were placed individually in transparent cylindrical containers measuring 20 cm in height and 14 cm in diameter. The containers were filled with water to a depth of 10 cm, and the water temperature was maintained at 25 ± 2 °C. Each test session lasted 6 min, and immobility time was quantified during the last 5 min. Immobility was defined as floating behavior with only the minimal movements required to keep the head above the water. A decrease in immobility time was interpreted as an antidepressant-like response [80].

#### 4.9.3. Antagonist-Coadministration Assessment of Antidepressant-like Activity

For the antagonist-coadministration assessment of antidepressant-like activity, each essential oil was tested at 100 mg/kg alone and in combination with WAY-100635 to determine whether the reduction in immobility was sensitive to 5-HT1A-related pharmacological modulation. Fluoxetine and fluoxetine plus WAY-100635 were included as pharmacological validation controls. Both the tail suspension test and the forced swim test were used as complementary antidepressant-sensitive behavioral paradigms to assess whether WAY-100635 consistently attenuated the EO100-induced reduction in immobility. Attenuation of the essential oil-induced reduction in immobility by WAY-100635 was interpreted as evidence compatible with possible participation of a 5-HT1A-related pathway [50,52,53]. This interpretation does not establish direct receptor mediation or exclude the contribution of additional neurotransmitter-related or pathway-convergent mechanisms.

### 4.10. Locomotor Activity Assessment

#### Open Field Test

The open field test was performed as an independent locomotor activity assessment to determine whether the behavioral effects of the essential oils could be explained by nonspecific motor stimulation, motor impairment, or sedative-like activity. Each essential oil was evaluated at 25, 50, and 100 mg/kg, and diazepam at 3.5 mg/kg was included as a sedative motor-control condition. The test was performed in a transparent acrylic open field arena (18 × 28 cm) divided into 12 squares of equal area. Each mouse was gently placed in the center of the apparatus and allowed to explore freely for 5 min. The number of squares crossed with all four paws was recorded by a blinded observer. The apparatus was cleaned between animals to minimize olfactory cues [81,82].

### 4.11. Exploratory Serum Corticosterone and Cytokine Profiling

Serum corticosterone and selected cytokines were quantified as exploratory peripheral neuroendocrine–immune correlates of acute essential oil exposure. To avoid the confounding influence of behavioral test-induced stress, biomarkers were assessed in an independent behavior-naïve subcohort not exposed to any behavioral paradigm. For each essential oil, a separate biomarker dose–response subcohort was used, including vehicle, EO25, EO50, and EO100, with eight mice per group. The animals received a single acute oral dose of vehicle or the corresponding essential oil. Ninety minutes after administration, blood was collected within the same light-phase sampling window for all biomarker groups to minimize circadian variability in the corticosterone and cytokine measurements. The mice were deeply anesthetized with 10% chloral hydrate (0.35 mL/100 g, i.p.) for terminal blood collection; cardiac puncture was performed only after loss of the pedal withdrawal reflex was confirmed, and euthanasia was completed immediately after blood collection according to institutional animal care procedures. The 90-min time point was selected to capture an acute posttreatment endocrine–immune window while avoiding direct contamination by behavioral test-induced stress responses [57,69].

Blood samples were allowed to clot at room temperature for 30 min and centrifuged at 3000 rpm for 10 min at 4 °C. Serum was aliquoted and stored at −80 °C until analysis. Corticosterone was quantified as an exploratory marker of HPA axis activation. IL-1β, IL-6, and TNF-α were quantified as proinflammatory cytokines, whereas IL-4 and IL-10 were quantified as anti-inflammatory cytokines. Analyte concentrations were determined using the following commercially available colorimetric ELISA kits according to the manufacturer’s instructions (Abcam, Cambridge, MA, USA): those of corticosterone (ab108821), IL-1β (ab197742), IL-4 (ab100710), IL-6 (ab222503), IL-10 (ab255729), and TNF-α (ab208348). The absorbance was measured at 450 nm using a BioTek Cytation 1 multimode microplate reader (Agilent Technologies, Santa Clara, CA, USA). Samples were assayed in duplicate, and concentrations were interpolated from standard curves using a four-parameter logistic model. Assay performance was monitored using the duplicate coefficient of variation and the standard curve fit. Because some cytokine concentrations were close to the lower analytical range of the assays, the serum cytokine findings were interpreted as exploratory peripheral correlates rather than definitive mechanistic mediators [69,83].

### 4.12. Statistical Analysis

The data are expressed as the mean ± SEM unless otherwise stated. Normality was assessed using the Shapiro–Wilk test, and homogeneity of variance was evaluated using Brown–Forsythe and Bartlett’s tests. Assumptions for parametric analysis were checked before ANOVA was applied.

To improve the interpretability of the behavioral statistical results, complementary effect-size estimates and confidence intervals are reported in Supplementary Table S10 for the behavioral dose–response, antagonist coadministration, and locomotor control outcomes. For one-way ANOVA models, eta-squared (η^2^) and omega-squared (ω^2^) were calculated as global effect-size indices from the ANOVA sums of squares and residual mean-square terms. For prespecified pairwise comparisons, mean differences, 95% confidence intervals, adjusted p values, and directional Hedges’ g values coded as Mean 2 minus Mean 1 were reported. When Brown–Forsythe or Bartlett tests suggested heterogeneity of variance, the corresponding behavioral findings were interpreted cautiously, with emphasis placed on prespecified contrasts, confidence intervals, and effect-size direction rather than on formal between-oil ranking.

Dose–response behavioral data were analyzed within each essential oil block using ordinary one-way ANOVA followed by Dunnett’s multiple comparisons test versus the corresponding vehicle-treated control. Spontaneous locomotor activity in the open field test was analyzed as an independent experiment using ordinary one-way ANOVA followed by Dunnett’s multiple comparisons test versus the vehicle-treated control.

Antagonist-coadministration assays were analyzed using ordinary one-way ANOVA followed by Sidak’s selected multiple comparisons test. For the anxiolytic-like antagonist-coadministration assays, the preselected comparisons were CTRL versus DZP, DZP versus DZP + FMZ, CTRL versus EO100, EO100 versus EO + FMZ, EO100 versus EO + WAY, CTRL versus EO + FMZ, and CTRL versus EO + WAY. For the antidepressant-like antagonist-coadministration assays, the preselected comparisons were CTRL versus FLX, FLX versus FLX + WAY, CTRL versus WAY, CTRL versus EO100, EO100 versus EO + WAY, and CTRL versus EO + WAY. Antagonist-induced attenuation of the EO100 response was interpreted as evidence compatible with possible participation of the corresponding neurotransmitter-related pathway, but not as proof of direct receptor mediation, functional receptor modulation, or exclusive pathway dependence.

Serum corticosterone and cytokine data were analyzed separately from the behavioral outcomes. For each essential oil, biomarker dose–response data were analyzed using ordinary one-way ANOVA followed by Dunnett’s multiple comparisons test versus the corresponding vehicle group. Because multiple exploratory biomarker comparisons were performed, Benjamini–Hochberg false discovery rate correction was applied across all 54 dose-versus-vehicle biomarker comparisons reported in Supplementary Table S7. For biomarker analyses, Dunnett-adjusted *p* values were interpreted together with BH-FDR q values, and q < 0.05 was considered evidence of robust FDR-significant biomarker modulation. For the behavioral and antagonist coadministration analyses, *p* < 0.05 was considered statistically significant. Analyses were performed using GraphPad Prism 8.0.1 (GraphPad Software, San Diego, CA, USA) and IBM SPSS Statistics 31.0 (IBM Corp., Armonk, NY, USA).

## 5. Conclusions

In conclusion, this study provides an integrated chemical, in silico, behavioral, pharmacological, and exploratory biomarker evaluation of the essential oils of *Satureja brevicalyx*, *Peperomia dolabriformis*, and *Rosmarinus officinalis* in acute mouse models related to anxiety- and depression-like behaviors. The three oils produced anxiolytic-like and antidepressant-like effects in male BALB/c mice without suppressing spontaneous locomotor activity. However, these findings should be interpreted within the limits of acute experimental conditions and independent treatment blocks. The GC-MS data define differentiated semi-quantitative chemical fingerprints, whereas the docking analyses provide predictive and hypothesis-generating ligand–target information. The antagonist assays showed flumazenil- and/or WAY-100635-sensitive behavioral attenuation patterns, supporting possible participation of the GABA-A/benzodiazepine- and 5-HT1A-related pathways, but not proving exclusive receptor mediation. SBEO showed a consistent flumazenil-sensitive anxiolytic-like profile and the clearest acute peripheral endocrine–immune modulation; PDEO showed a broad predicted multitarget profile with mixed antagonist-sensitive attenuation; and ROEO showed significant but more moderate effects. Overall, the findings justify further mechanism-oriented investigation, but translational interpretation requires confirmation using experimentally calculated retention indices and/or authentic standards for key constituents, functional receptor assays, chronic stress-based models, pharmacokinetic studies, both-sex designs, and expanded subacute/chronic toxicological assessments before any application-oriented inference is made.

## Figures and Tables

**Table 1:** Major GC-MS-annotated constituents of the essential oils of *Satureja brevicalyx*, *Rosmarinus officinalis*, and *Peperomia dolabriformis*.

No.	Compound	RI	*S. brevicalyx*	*P. dolabriformis*	*R. officinalis*
			Area (%)	Area (%)	Area (%)
1	Nonane	900	ND	1.02	ND
2	α-Pinene	932–939	0.25	1.35	7.32
3	Camphene	954	ND	ND	4.02
4	β-Pinene	974–979	0.23	1.64	4.93
5	β-Myrcene	988–991	0.21	0.44	1.39
6	o-Cymene	1026–1030	1.52	ND	0.07
7	Limonene	1024–1031	10.01	13.60	10.81
8	1,8-Cineole (eucalyptol)	1033	0.95	ND	27.40
9	γ-Terpinene	1060–1062	6.10	ND	0.57
10	n-Butylbenzene	1068	ND	4.25	ND
11	Linalool	1098	21.37	ND	6.75
12	Menthone	1145	10.41	ND	ND
13	Isomenthone	1155	5.95	ND	ND
14	trans-Dihydrocarvone	1169 *	2.82	ND	ND
15	Camphor	1145	ND	ND	5.05
16	α-Terpineol	1186–1189	3.40	0.08	16.38
17	Pulegone	1237	11.24	ND	ND
18	Bornyl acetate	1285	ND	ND	4.58
19	Orcinol dimethyl ether	1274	ND	4.05	ND
20	5-tert-Butyl-1,3-benzodioxole	1400 *	ND	23.19	ND
21	β-Caryophyllene	1417–1419	7.99	0.89	0.73
22	Ishwarane	1468	ND	8.83	ND
23	γ-Amorphene	1495	ND	1.00	ND
24	Eremophilene	1498 *	ND	1.24	ND
25	Bicyclogermacrene	1494–1500	8.11	0.78	0.38
26	Myristicin	1517–1518	ND	10.83	0.28
27	Elemicin	1555	ND	5.49	ND
28	τ-Cadinol	1640–1642	0.06	3.84	ND
29	Ishwarol B	1674	ND	6.11	ND

Note. Only constituents with a relative TIC-area abundance ≥1.0% in at least one essential oil are shown; values below 1.0% were retained when the same constituent was also detected in another oil to facilitate cross-oil comparison. Values are expressed as normalized GC-MS total ion current (TIC) peak-area percentages and should be interpreted as semi-quantitative relative abundances rather than absolute concentrations. RI = reference retention index or reference RI range reported for nonpolar or slightly polar 5%–phenyl-methylpolysiloxane columns; ND = not detected or not reported; EI-MS = electron ionization mass spectrometry; GC-FID = gas chromatography–flame ionization detection; RT = retention time. Because a homologous n-alkane series was not acquired under the same chromatographic conditions, the RI values should be interpreted as indicative literature/reference RI values rather than definitive experimentally calculated retention indices. Compound annotations were based on EI-MS spectral-library matching, supported, when possible, by RI compatibility with the literature values. No authentic standards, GC-FID response-factor correction, or internal-standard-based quantification were used; therefore, the assignments should be considered putative GC-MS annotations. Asterisks indicate tentative assignments or RI/RT uncertainty, including the annotation of 5-tert-butyl-1,3-benzodioxole. Full GC-MS composition tables, including retention times and annotation details for each oil, are provided in Supplementary Tables S1–S3.

**Table 2:** Preliminary acute oral toxicity profile of the essential oils using a modified Lorke approach.

Essential Oil	Dose Range Tested (mg/kg, p.o.)	Mortality at 5000 mg/kg	Main Clinical Observations at High Doses	Estimated Oral LD50
*Satureja brevicalyx* essential oil	10–5000	0/3	Mild transient piloerection, reduced grooming, hypoactivity, and mild ataxia at 1600–5000 mg/kg; recovery by 24 h	>5000 mg/kg
*Peperomia dolabriformis* essential oil	10–5000	0/3	Mild-to-moderate transient hypoactivity, mild ataxia, and reduced grooming at 1600–5000 mg/kg; recovery by 24 h	>5000 mg/kg
*Rosmarinus officinalis* essential oil	10–5000	0/3	Mild-to-moderate transient hypoactivity, reduced grooming, and piloerection at 1600–5000 mg/kg; recovery by 24 h	>5000 mg/kg

Note. Preliminary acute oral toxicity was evaluated using a modified Lorke approach, with reference to the upper-dose-limit principles of OECD Test Guideline N°. 423. A complete dose-by-dose clinical observation table is provided in Supplementary Table S8. The oral LD50 was estimated to be greater than 5000 mg/kg because no mortality was observed at the highest tested dose within the 24-h observation window. p.o. = oral administration.

**Table 3:** Exploratory serum corticosterone levels and cytokine profiles after acute administration of the effective nonsedative dose of essential oils.

Essential Oil	Biomarker	Vehicle (Mean ± SEM)	EO100 (Mean ± SEM)	Change (%)	Dunnett-Adjusted *p* Value	BH-FDR q Value	Interpretation
*Satureja brevicalyx*	Corticosterone (ng/mL)	83.97 ± 1.84	75.57 ± 0.86	−10.0	<0.0001	0.0021	FDR-significant modest decrease
*Satureja brevicalyx*	IL-6 (pg/mL)	32.35 ± 1.36	28.65 ± 0.61	−11.5	0.0211	0.1762	Nominal decrease; not FDR-significant
*Satureja brevicalyx*	TNF-α (pg/mL)	15.67 ± 1.24	11.19 ± 0.36	−28.6	0.0008	0.0227	FDR-significant marked decrease
*Satureja brevicalyx*	IL-1β (pg/mL)	30.43 ± 1.13	26.72 ± 0.75	−12.2	0.0228	0.1762	Nominal decrease; not FDR-significant
*Satureja brevicalyx*	IL-10 (pg/mL)	25.30 ± 1.09	29.20 ± 0.70	+15.4	0.0137	0.1476	Nominal increase; not FDR-significant
*Satureja brevicalyx*	IL-4 (pg/mL)	19.27 ± 0.89	23.45 ± 0.90	+21.7	0.0013	0.0236	FDR-significant marked increase
*Peperomia dolabriformis*	Corticosterone (ng/mL)	83.23 ± 1.61	79.53 ± 2.64	−4.4	0.4399	>0.9999	No robust change
*Peperomia dolabriformis*	IL-6 (pg/mL)	34.21 ± 1.24	33.67 ± 1.78	−1.6	0.9869	>0.9999	No robust change
*Peperomia dolabriformis*	TNF-α (pg/mL)	18.52 ± 0.70	16.85 ± 0.73	−9.1	0.2809	0.9708	No robust change
*Peperomia dolabriformis*	IL-1β (pg/mL)	31.22 ± 1.34	29.36 ± 1.34	−6.0	0.6311	>0.9999	No robust change
*Peperomia dolabriformis*	IL-10 (pg/mL)	25.14 ± 1.18	25.86 ± 1.74	+2.9	0.9693	>0.9999	No robust change
*Peperomia dolabriformis*	IL-4 (pg/mL)	17.62 ± 1.52	18.31 ± 1.58	+3.9	0.9700	>0.9999	No robust change
*Rosmarinus officinalis*	Corticosterone (ng/mL)	81.98 ± 1.67	76.81 ± 2.62	−6.3	0.1954	0.8118	No robust change
*Rosmarinus officinalis*	IL-6 (pg/mL)	35.35 ± 0.82	34.59 ± 1.95	−2.1	0.9511	>0.9999	No robust change
*Rosmarinus officinalis*	TNF-α (pg/mL)	16.56 ± 1.11	14.22 ± 1.20	−14.2	0.2876	0.9708	No robust change
*Rosmarinus officinalis*	IL-1β (pg/mL)	30.45 ± 1.32	27.85 ± 1.21	−8.5	0.3152	>0.9999	No robust change
*Rosmarinus officinalis*	IL-10 (pg/mL)	23.53 ± 1.24	24.53 ± 1.77	+4.3	0.9378	>0.9999	No robust change
*Rosmarinus officinalis*	IL-4 (pg/mL)	18.39 ± 1.28	19.35 ± 1.92	+5.3	0.9357	>0.9999	No robust change

Note. The values are the means ± SEMs; n = 8 per group. Dunnett-adjusted *p* values compare EO100 versus the corresponding vehicle. BH-FDR q values were calculated across all 54 dose-versus-vehicle biomarker comparisons reported in Supplementary Table S7. Biomarkers were assessed in behavior-naïve, nonstress-challenged animals and are interpreted as exploratory peripheral neuroendocrine–immune correlations.

## Data Availability

The data presented in this study are available in the article and Supplementary Materials. Raw datasets generated and analyzed during the current study, including behavioral, GC-MS, ELISA, toxicity, and molecular docking data, are available from the corresponding author upon reasonable request.

## Supplementary Materials

The following supporting information can be downloaded at: https://www.mdpi.com/article/10.3390/molecules31132378/s1, Supplementary Figure S1. GC–MS TIC chromatogram of *Satureja brevicalyx* essential oil; Supplementary Figure S2. Expanded region (3–21 min) of the GC–MS TIC chromatogram in Supplementary Figure S1; Supplementary Figure S3. Expanded region (21–50 min) of the GC–MS TIC chromatogram in Supplementary Figure S1; Supplementary Figure S4. GC–MS TIC chromatogram of *Peperomia dolabriformis* essential oil; Supplementary Figure S5. Expanded region (3–30 min) of the GC–MS TIC chromatogram in Supplementary Figure S4; Supplementary Figure S6. Expanded region (30–50 min) of the GC–MS TIC chromatogram in Supplementary Figure S4; Supplementary Figure S7. GC–MS TIC chromatogram of *Rosmarinus officinalis* essential oil; Supplementary Figure S8. Expanded region (3–25 min) of the GC–MS TIC chromatogram in Supplementary Figure S7; Supplementary Figure S9. Expanded region (25–50 min) of the GC–MS TIC chromatogram in Supplementary Figure S7; Supplementary Table S1. Chemical composition of *Satureja brevicalyx* essential oil; Supplementary Table S2. Chemical composition of the essential oil of *Peperomia dolabriformis*; Supplementary Table S3. Chemical composition of *Rosmarinus officinalis* essential oil; Supplementary Table S4. Full docking matrix of the *Satureja brevicalyx* essential oil; Supplementary Table S5. Full docking matrix of the *Peperomia dolabriformis* essential oil; Supplementary Table S6. Full docking matrix of the *Rosmarinus officinalis* essential oil; Supplementary Table S7. Complete exploratory serum corticosterone and cytokine profile across all tested doses of the essential oils; Supplementary Table S8. Complete dose-by-dose preliminary acute oral toxicity observations for the three essential oils; Supplementary Table S9. Integrated interpretation of antagonist-sensitive anxiolytic- and antidepressant-like behavioral patterns of the essential oils; Supplementary Table S10. Effect sizes and confidence intervals for behavioral, antagonist-coadministration, and locomotor-control outcomes [84,85,86].

## Abbreviations

The following abbreviations are used in this manuscript:

Abbreviation	Definition
BH-FDR	Benjamini–Hochberg false discovery rate
CB1	Cannabinoid receptor type 1
CB2	Cannabinoid receptor type 2
CRHR1	Corticotropin-releasing hormone receptor 1
CTRL	Vehicle-treated control group
DZP	Diazepam
EI	Electron ionization
ELISA	Enzyme-linked immunosorbent assay
EO	Essential oil
EO25	Essential oil at 25 mg/kg
EO50	Essential oil at 50 mg/kg
EO100	Essential oil at 100 mg/kg
EPM	Elevated plus maze
FLX	Fluoxetine
FMZ	Flumazenil
FST	Forced swim test
GABA-A	Gamma-aminobutyric acid type A receptor
GC–MS	Gas chromatography-mass spectrometry
GR	Glucocorticoid receptor
HPA	Hypothalamic–pituitary–adrenal
IL	Interleukin
LDB	Light–dark box
MAO-A	Monoamine oxidase A
MAO-B	Monoamine oxidase B
MBT	Marble burying test
MR	Mineralocorticoid receptor
MT1	Melatonin receptor type 1
MT2	Melatonin receptor type 2
ND	Not detected or not reported
OAE	Percentage of open arm entries
OAT	Percentage of time spent in the open arms
OX2R	Orexin receptor type 2
PDEO	*Peperomia dolabriformis* essential oil
RI	Retention index
ROEO	*Rosmarinus officinalis* essential oil
SBEO	*Satureja brevicalyx* essential oil
SEM	Standard error of the mean
SERT	Serotonin transporter
TIC	Total ion chromatogram
TNF-α	Tumor necrosis factor alpha
TST	Tail suspension test
WAY	WAY-100635
5-HT1A	5-HT1A
