# Supplementary material for: Integrated Chemical, In Silico, and Functional Neurobehavioral Evaluation of Three Essential Oils in Acute Anxiety- and Depression-Related Mouse Models
Source: Molecules. 2026 Jul 6;31(13):2378. doi: 10.3390/molecules31132378 (PMC13362989; doi:10.3390/molecules31132378)
Supplement: Supplementary file 1 [file molecules-31-02378-s001.zip › Supplementary Figures S1-S9.pdf]

Supplementary Figures

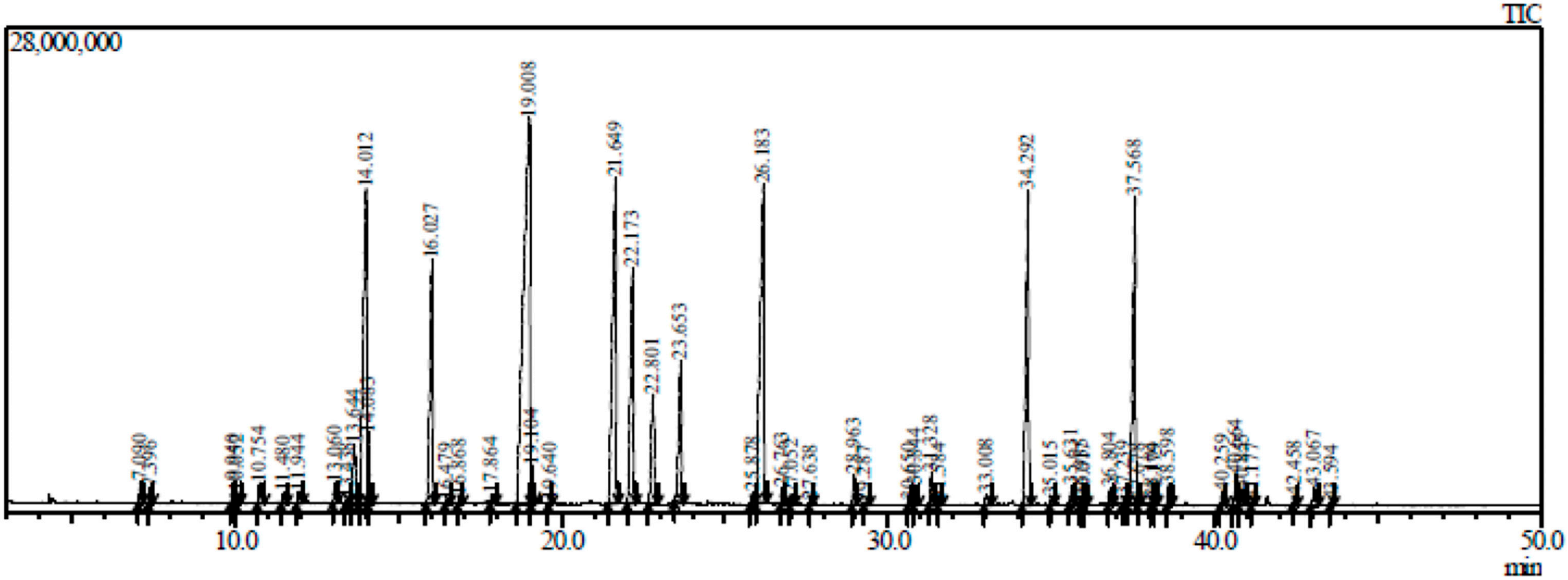

Supplementary Figure S1. GC‑MS TIC chromatogram of *Satureja brevicalyx* essential oil (Shimadzu GC-2010 Plus/GCMS-QP2010 Ultra, EI 70 eV; Rtx-5MS 30 m × 0.25 mm, 0.25 µm; He, linear-velocity control; oven 50→300 ° C program; see Methods).

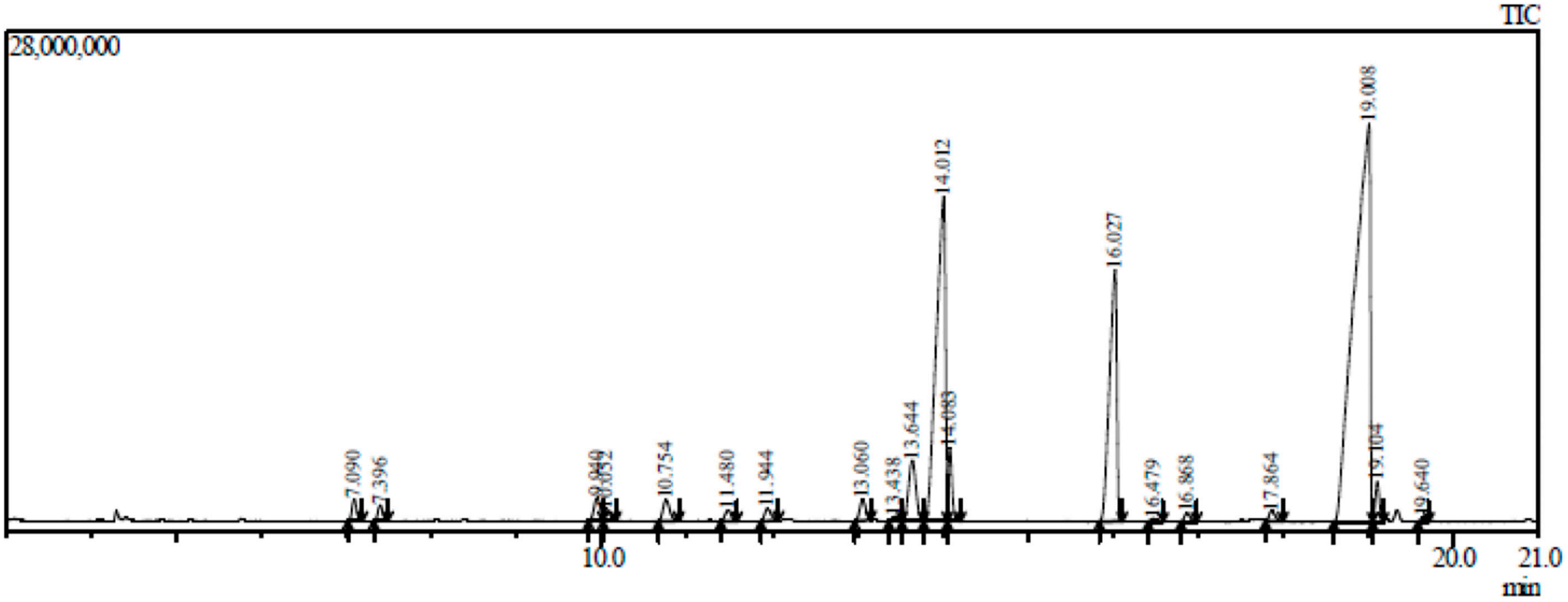

Supplementary Figure S2. Expanded region (3–21 min) of the GC–MS TIC chromatogram in Supplementary Figure S1.

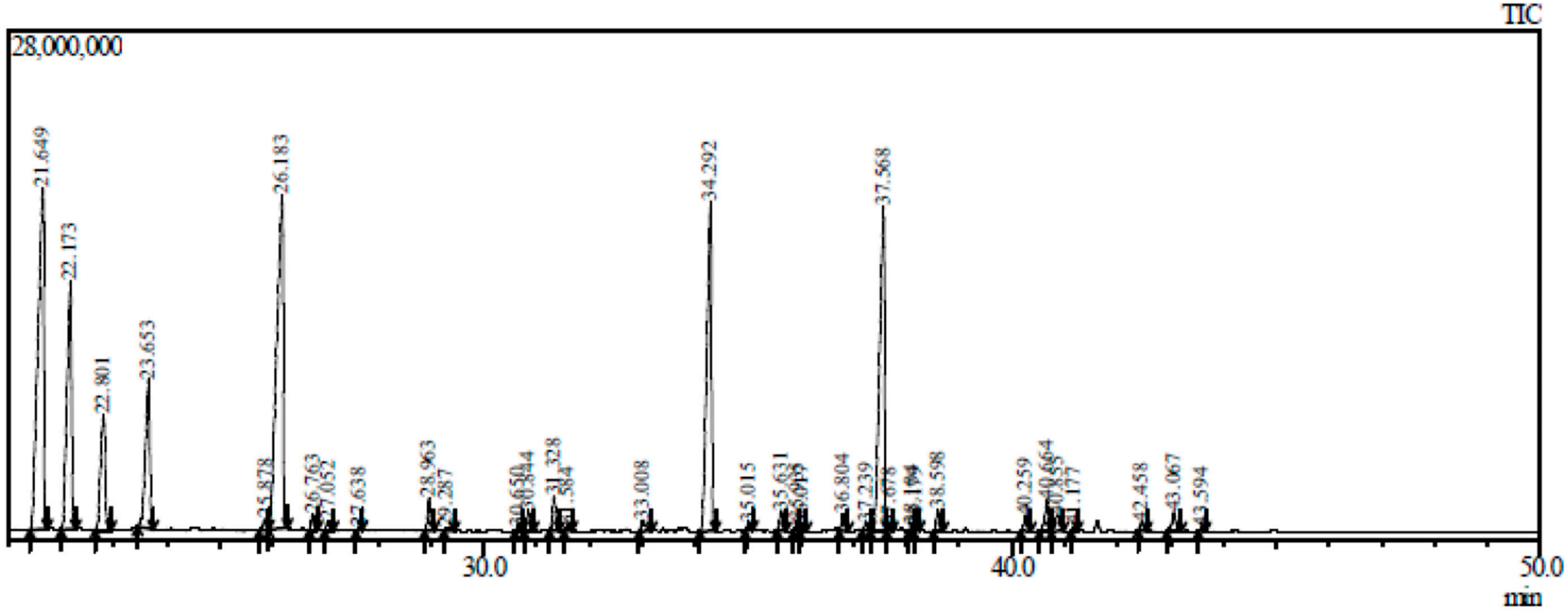

Supplementary Figure S3. Expanded region (21–50 min) of the GC–MS TIC chromatogram in Supplementary Figure S1.

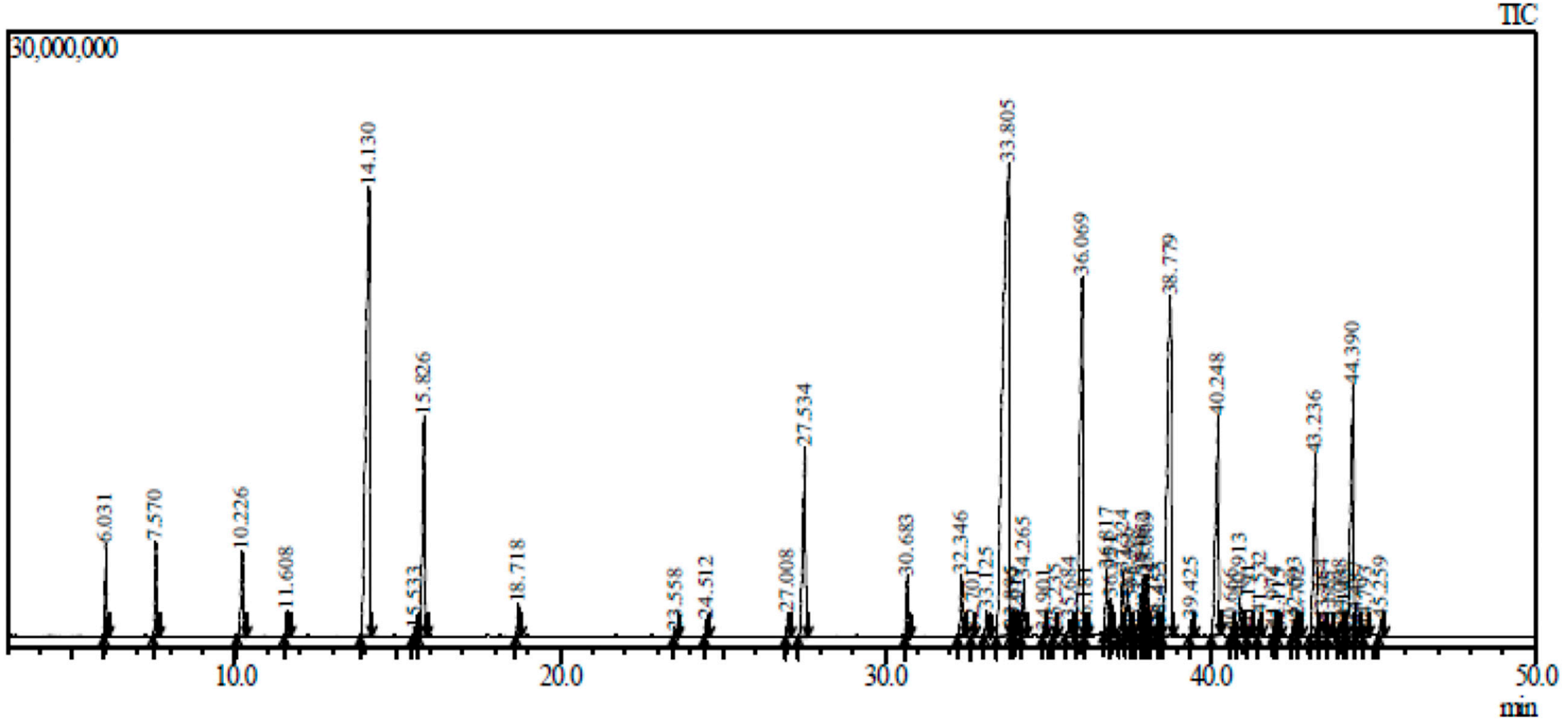

Supplementary Figure S4. GC-MS TIC chromatogram of *Peperomia dolabriformis* essential oil (Shimadzu GC-2010 Plus/GCMS-QP2010 Ultra, EI 70 eV; Rtx-5MS 30 m × 0.25 mm, 0.25 µm; He, linear-velocity control; oven 50→300 ° C program; see Methods).

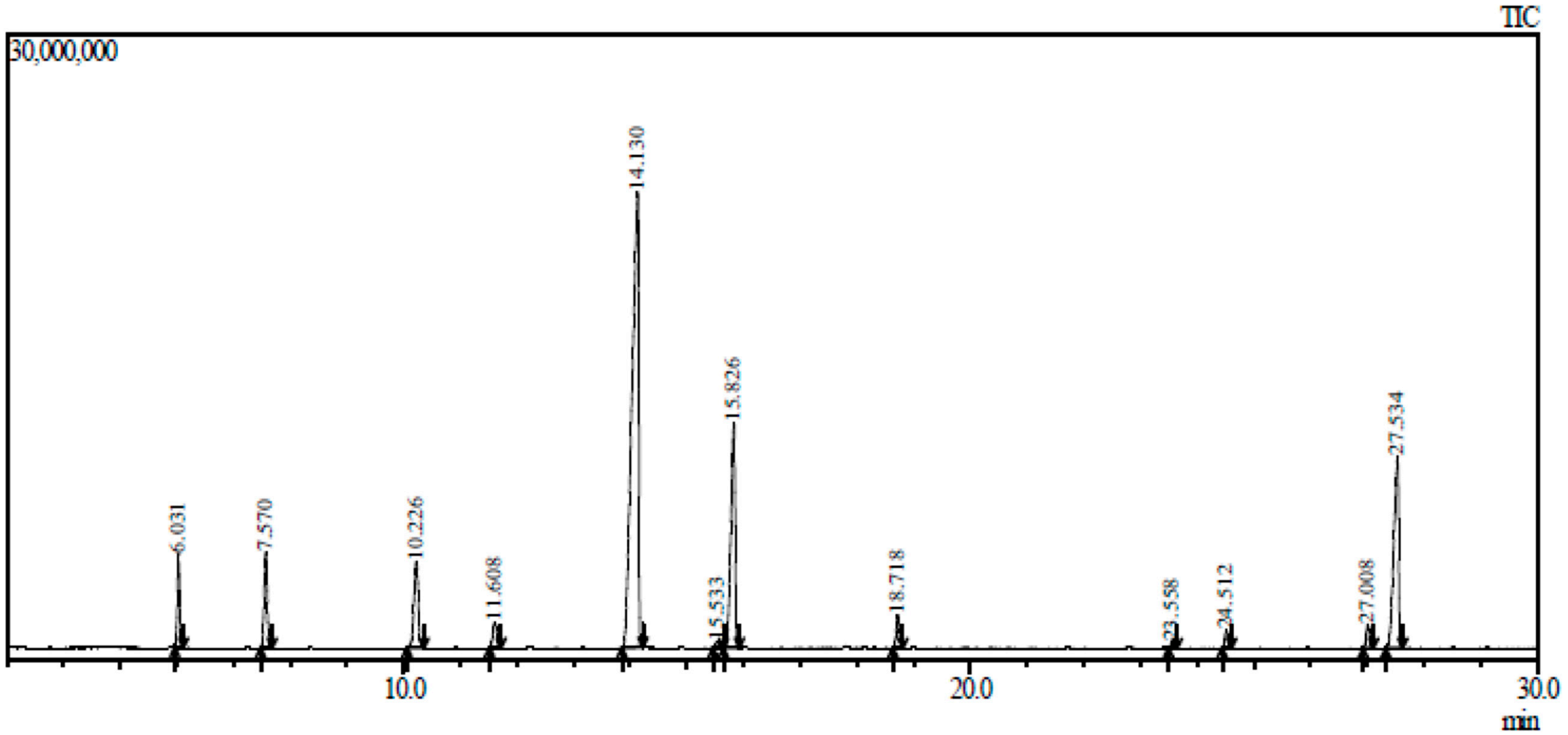

Supplementary Figure S5. Expanded region (3–30 min) of the GC–MS TIC chromatogram in Supplementary Figure S4.

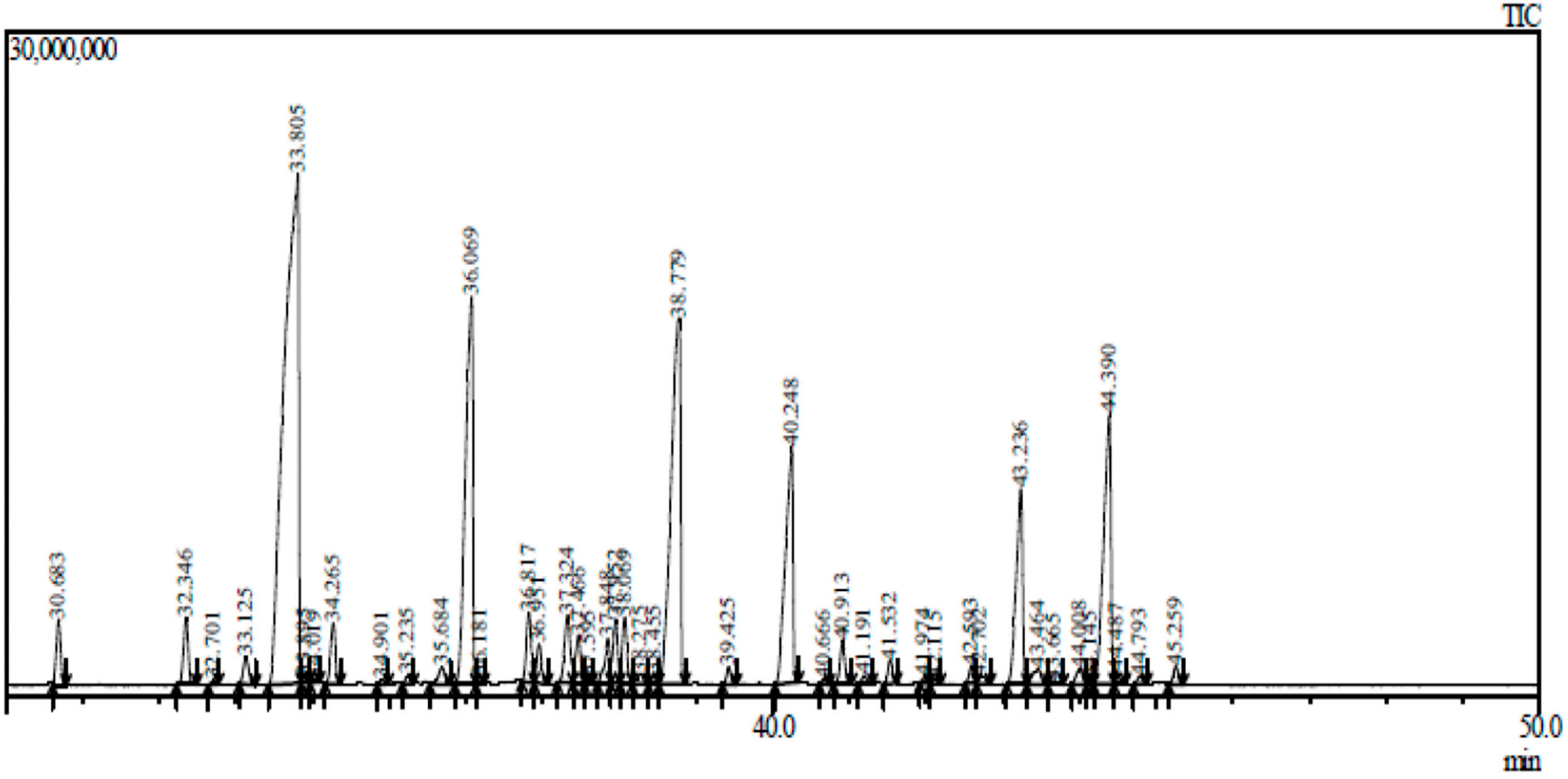

Supplementary Figure S6. Expanded region (30–50 min) of the GC–MS TIC chromatogram in Supplementary Figure S4.

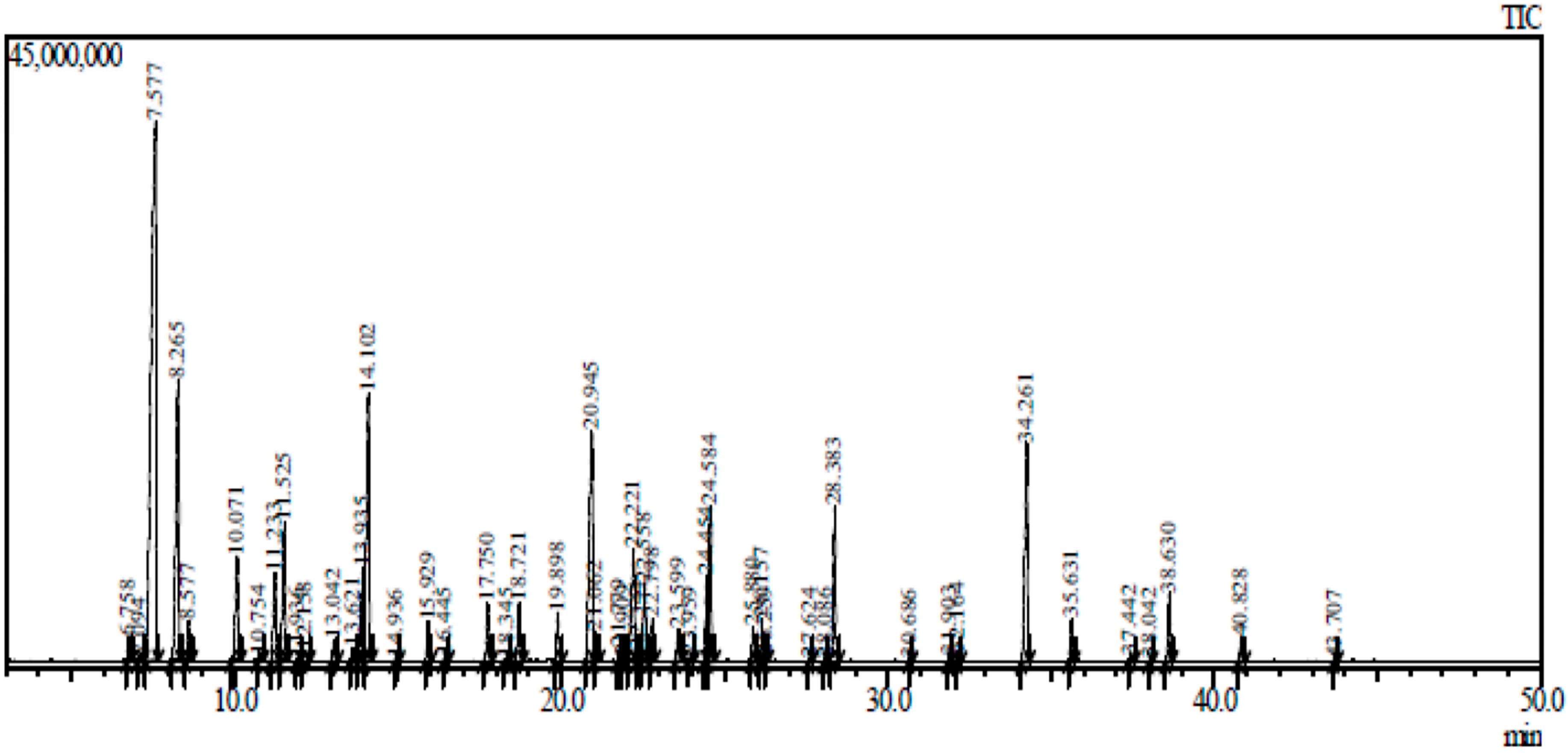

Supplementary Figure S7. GC‑MS TIC chromatogram of *Rosmarinus officinalis* essential oil (Shimadzu GC-2010 Plus/GCMS-QP2010 Ultra, EI 70 eV; Rtx-5MS 30 m × 0.25 mm, 0.25 µm; He, linear-velocity control; oven 50→300 ° C program; see Methods).

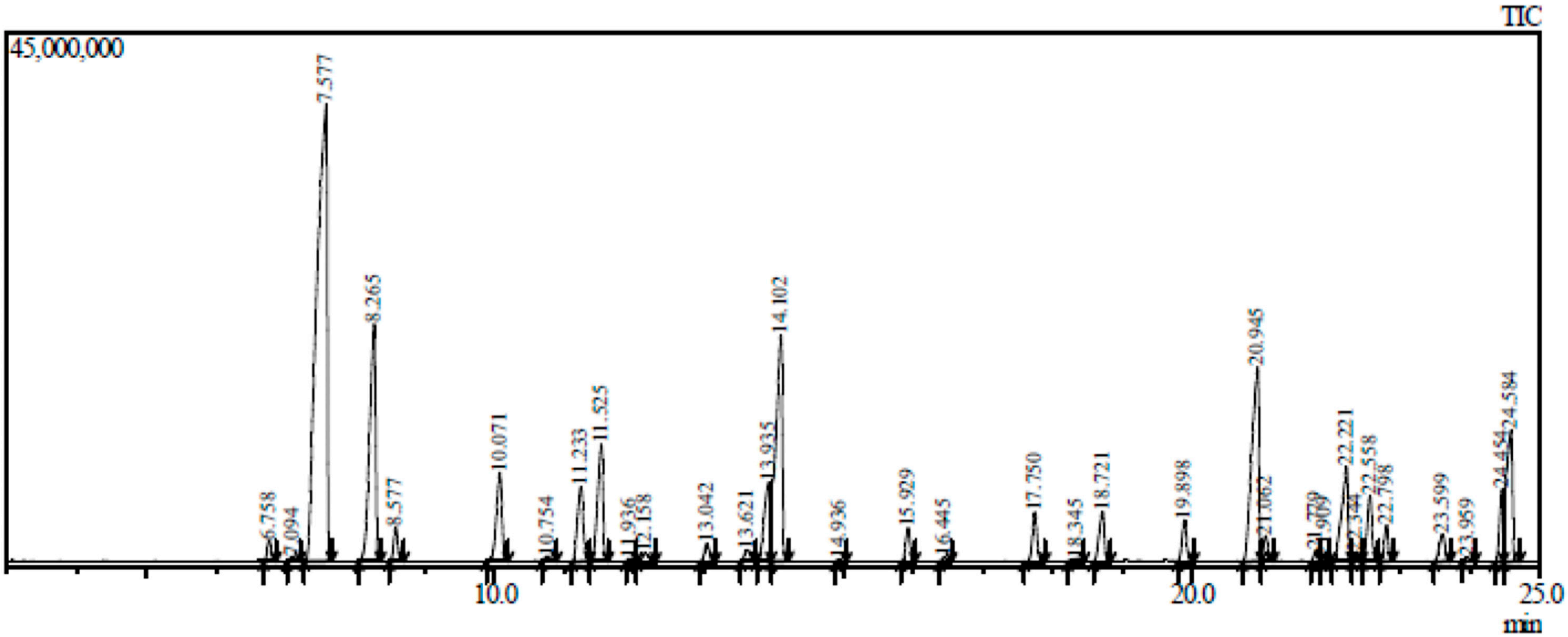

Supplementary Figure S8. Expanded region (3–25 min) of the GC–MS TIC chromatogram in Supplementary Figure S7.

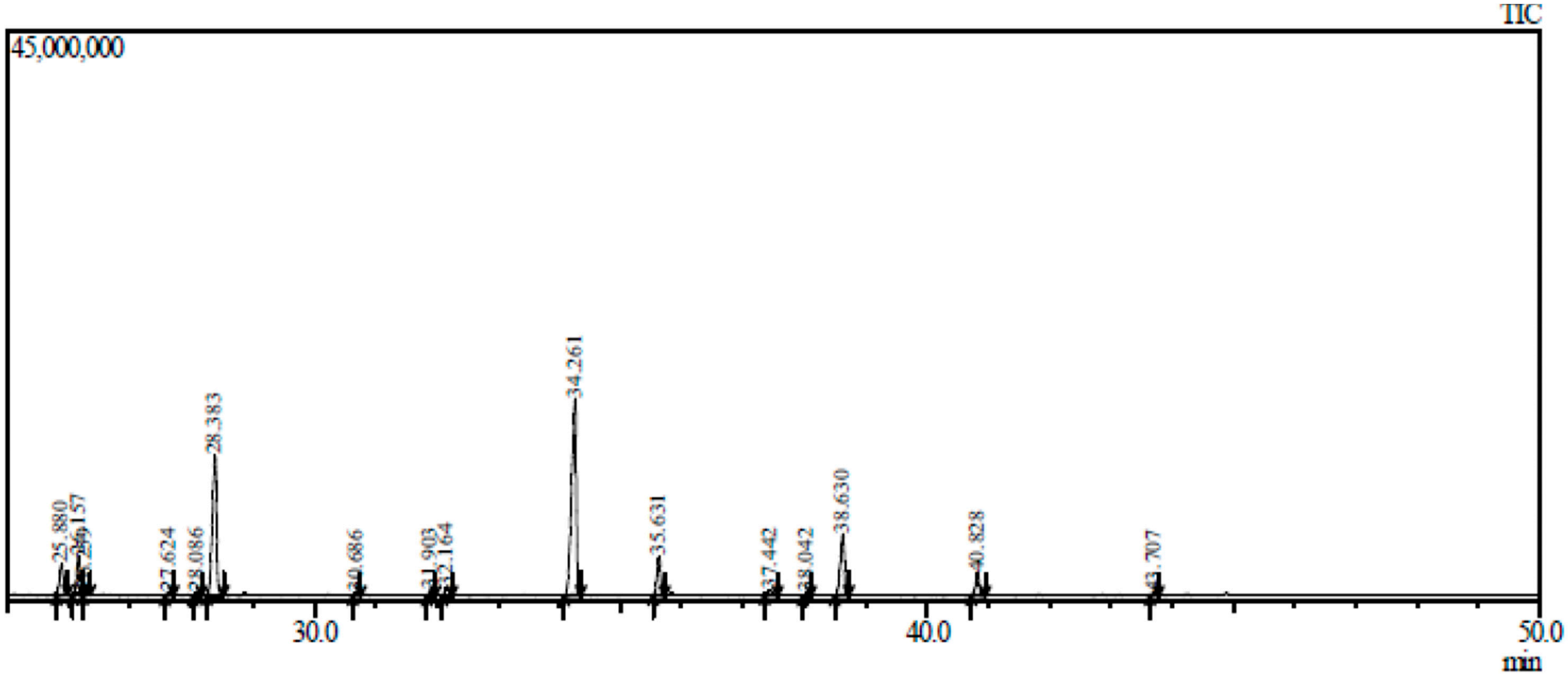

Supplementary Figure S9. Expanded region (25–50 min) of the GC–MS TIC chromatogram in Supplementary Figure S7.
