# Supplementary material for: Integrated Chemical, In Silico, and Functional Neurobehavioral Evaluation of Three Essential Oils in Acute Anxiety- and Depression-Related Mouse Models
Source: Molecules. 2026 Jul 6;31(13):2378. doi: 10.3390/molecules31132378 (PMC13362989; doi:10.3390/molecules31132378)
Supplement: Supplementary file 1 [file molecules-31-02378-s001.zip › Supplementary Table S1 Satureja_brevicalyx esential oil composition.pdf]

Supplementary Table S1. Chemical composition of *Satureja brevicalyx* essential oil

N°	Compound	RT (min)	RI	Area (%)
1	α-Thujene	7.090	924	0.33
2	α-Pinene	7.396	939	0.25
3	Sabinene	9.940	975	0.50
4	β-Pinene	10.052	979	0.23
5	1-Octen-3-ol	10.754	981	0.48
6	β-Myrcene	11.480	991	0.21
7	3-Octanol	11.944	999	0.26
8	α-Terpinene	13.060	1017	0.41
9	2-Methylbutyl isobutyrate	13.438	1025*	0.10
10	o-Cymene	13.644	1030	1.52
11	Limonene	14.012	1031	10.01
12	Eucalyptol (1,8-cineole)	14.083	1033	0.95
13	γ-Terpinene	16.027	1062	6.10
14	cis-Sabinene hydrate	16.479	1066	0.06
15	cis-Linalool oxide (furanoid)	16.868	1085	0.16
16	trans-Linalool oxide (furanoid)	17.864	1092	0.20
17	Linalool	19.008	1098	21.37
18	Isovalerate ester	19.104	1100*	0.56
19	1-Octen-3-yl acetate	19.640	1109	0.06
20	Menthone	21.649	1145	10.41
21	Isomenthone	22.173	1155	5.95
22	trans-Dihydrocarvone	22.801	1169*	2.82
23	α-Terpineol	23.653	1189	3.40
24	Citronellol	25.878	1227	0.27
25	Pulegone	26.183	1237	11.24
26	Pinocarvone	26.763	1165†	0.26
27	Monoterpene ketone (p-menthenone derivative)	27.052	1253*	0.19
28	Isopinocarvone	27.638	1175†	0.05
29	Thymol	28.963	1289	0.51
30	Cadinane derivative	29.287	1316*	0.07
31	γ-Elemene	30.650	1432†	0.08
32	Piperitenone	30.844	1340†	0.33
33	α-Terpinyl acetate	31.328	1349	0.54
34	Thymol methyl ether	31.584	1235†	0.08
35	Geranyl acetate	33.008	1386	0.26
36	β-Caryophyllene	34.292	1418	7.99
37	Aromadendrene	35.015	1436	0.12
38	α-Humulene	35.631	1452	0.32
39	Alloaromadendrene	35.935	1458	0.17

N°	Compound	RT (min)	RI	Area (%)
40	Germacrene D derivative	36.017	1480†	0.10
41	β-Copaene	36.804	1432†	0.29
42	Phenethyl butyrate	37.239	1447*	0.12
43	Bicyclogermacrene	37.568	1500	8.11
44	α-Muurolene	37.678	1501	0.07
45	α-Farnesene	38.104	1507	0.10
46	Cadinene isomer	38.179	1513*	0.07
47	Cadinene/calamene-type sesquiterpene	38.598	1516*	0.35
48	(E)-β-Farnesene	40.259	1458†	0.24
49	Spathulenol	40.664	1577	0.61
50	Unidentified compound (reported as cembrene; RI mismatch)	40.855	1577†	0.42
51	Azulene isomer	41.177	1578*	0.07
52	trans-Longipinocarveol	42.458	1581*	0.13
53	Caryophyllene oxide	43.067	1583	0.43
54	τ-Cadinol	43.594	1642	0.06

Notes. RI values are indicative literature/reference retention indices for non-polar or slightly polar 5%-phenyl-methylpolysiloxane columns and should not be interpreted as experimentally calculated RI values because a homologous n-alkane series was not acquired under the same chromatographic conditions. Compounds marked with an asterisk (*) are tentative or broad assignments. Compounds marked with a dagger (†) showed RI/RT uncertainty and were retained as tentative annotations. Compound assignments were based on mass spectral library matching and comparison with reference RI values from NIST Chemistry WebBook, Adams' database, Babushok et al. (2011), and other literature sources. Reference sources used for RI verification: NIST Chemistry WebBook GC retention data; Babushok, Linstrom and Zenkevich (2011), Journal of Physical and Chemical Reference Data, 40, 043101; Adams, R. P. (2007), Identification of Essential Oil Components by Gas Chromatography/Mass Spectrometry, 4th ed.[84–86].
