# Supplementary material for: Integrated Chemical, In Silico, and Functional Neurobehavioral Evaluation of Three Essential Oils in Acute Anxiety- and Depression-Related Mouse Models
Source: Molecules. 2026 Jul 6;31(13):2378. doi: 10.3390/molecules31132378 (PMC13362989; doi:10.3390/molecules31132378)
Supplement: Supplementary file 1 [file molecules-31-02378-s001.zip › Supplementary Table S10 Effect sizes and CIs behavioral.pdf]

Supplementary Table S10. Effect sizes and confidence intervals for behavioral, antagonist-coadministration, and locomotor-control outcomes

This supplementary table reports global one-way ANOVA statistics and prespecified pairwise contrasts for the behavioral dose-response assays, open-field locomotor-control assessment, and antagonist-coadministration assays. The table is intended to improve transparency regarding the magnitude and precision of the observed effects, without introducing formal between-oil rankings.

Table S10A. Global one-way ANOVA statistics and effect-size estimates

ID	Phase	Oil	Test	Outcome	k/N	F(df1,df2)	p	η²	ω²	Variance
A019	Dose-response anxiety-like	PDEO	EPM	% open-arm entries	5/40	F(4,35) = 745.2	<0.0001	0.988	0.987	No flag
A020	Dose-response anxiety-like	PDEO	EPM	Open-arm time (%)	5/40	F(4,35) = 185.5	<0.0001	0.955	0.949	Variance flag
A021	Dose-response anxiety-like	PDEO	LDB	Light-compartment time (%)	5/40	F(4,35) = 167.5	<0.0001	0.950	0.943	No flag
A022	Dose-response anxiety-like	PDEO	MBT	Buried marbles (n)	5/40	F(4,35) = 44.77	<0.0001	0.837	0.814	No flag
A023	Dose-response anxiety-like	ROEO	EPM	% open-arm entries	5/40	F(4,35) = 550.4	<0.0001	0.984	0.982	No flag
A024	Dose-response anxiety-like	ROEO	EPM	Open-arm time (%)	5/40	F(4,35) = 141.7	<0.0001	0.942	0.934	No flag
A025	Dose-response anxiety-like	ROEO	LDB	Light-compartment time (%)	5/40	F(4,35) = 183.8	<0.0001	0.955	0.948	Variance flag
A026	Dose-response anxiety-like	ROEO	MBT	Buried marbles (n)	5/40	F(4,35) = 68.27	<0.0001	0.886	0.871	No flag
A027	Dose-response anxiety-like	SBEO	EPM	% open-arm entries	5/40	F(4,35) = 1381	<0.0001	0.994	0.993	No flag
A028	Dose-response anxiety-like	SBEO	EPM	Open-arm time (%)	5/40	F(4,35) = 214.4	<0.0001	0.961	0.955	Variance flag
A029	Dose-response anxiety-like	SBEO	LDB	Light-compartment time (%)	5/40	F(4,35) = 285.1	<0.0001	0.970	0.966	No flag
A030	Dose-response anxiety-like	SBEO	MBT	Buried marbles (n)	5/40	F(4,35) = 68.5	<0.0001	0.887	0.871	Variance flag
A013	Dose-response depression-like	PDEO	FST	Immobility (s)	5/40	F(4,35) = 3160	<0.0001	0.997	0.997	No flag
A014	Dose-response depression-like	PDEO	TST	Immobility (s)	5/40	F(4,35) = 1733	<0.0001	0.995	0.994	No flag
A015	Dose-response depression-like	ROEO	FST	Immobility (s)	5/40	F(4,35) = 4074	<0.0001	0.998	0.998	No flag
A016	Dose-response depression-like	ROEO	TST	Immobility (s)	5/40	F(4,35) = 3178	<0.0001	0.997	0.997	No flag
A017	Dose-response depression-like	SBEO	FST	Immobility (s)	5/40	F(4,35) = 3649	<0.0001	0.998	0.997	No flag

ID	Phase	Oil	Test	Outcome	k/N	F(df1,df2)	p	η²	ω²	Variance
A018	Dose-response depression-like	SBEO	TST	Immobility (s)	5/40	F(4,35) = 2826	<0.0001	0.997	0.996	No flag
A031	Locomotor control	All oils	OFT	Line crossings	11/88	F(10,77) = 117.4	<0.0001	0.938	0.930	No flag
A007	Antagonist coadministration anxiety-like	PDEO	EPM	% open-arm entries	6/48	F(5,42) = 317.9	<0.0001	0.974	0.971	No flag
A008	Antagonist coadministration anxiety-like	PDEO	EPM	Open-arm time (%)	6/48	F(5,42) = 105.8	<0.0001	0.926	0.916	Variance flag
A009	Antagonist coadministration anxiety-like	ROEO	EPM	% open-arm entries	6/48	F(5,42) = 248.4	<0.0001	0.967	0.963	No flag
A010	Antagonist coadministration anxiety-like	ROEO	EPM	Open-arm time (%)	6/48	F(5,42) = 88.27	<0.0001	0.913	0.901	No flag
A011	Antagonist coadministration anxiety-like	SBEO	EPM	% open-arm entries	6/48	F(5,42) = 641.0	<0.0001	0.987	0.985	Variance flag
A012	Antagonist coadministration anxiety-like	SBEO	EPM	Open-arm time (%)	6/48	F(5,42) = 105.1	<0.0001	0.926	0.916	Variance flag
A001	Antagonist coadministration depression-like	PDEO	FST	Immobility (s)	6/48	F(5,42) = 1025	<0.0001	0.992	0.991	Variance flag
A002	Antagonist coadministration depression-like	PDEO	TST	Immobility (s)	6/48	F(5,42) = 766.9	<0.0001	0.989	0.988	Variance flag
A003	Antagonist coadministration depression-like	ROEO	FST	Immobility (s)	6/48	F(5,42) = 1188	<0.0001	0.993	0.992	Variance flag
A004	Antagonist coadministration depression-like	ROEO	TST	Immobility (s)	6/48	F(5,42) = 921.2	<0.0001	0.991	0.990	Variance flag
A005	Antagonist coadministration depression-like	SBEO	FST	Immobility (s)	6/48	F(5,42) = 821.0	<0.0001	0.990	0.988	Variance flag
A006	Antagonist coadministration depression-like	SBEO	TST	Immobility (s)	6/48	F(5,42) = 818.4	<0.0001	0.990	0.988	Variance flag

Note. k/N = number of groups/total observations. η^2 was derived from the one-way ANOVA R-squared value. ω^2 was calculated as a conservative global effect-size index using the ANOVA sums of squares and residual mean-square terms. The variance flag indicates that either the Brown-Forsythe or Bartlett test suggested heterogeneity of variance at $p < 0.05$; these rows should therefore be interpreted cautiously, emphasizing prespecified contrasts, confidence intervals, and effect direction rather than formal ranking. Overall, global effects were large across the behavioral dose-response and antagonist-coadministration endpoints. The very large F statistics observed in several endpoints were consistent with high between-group separation and low residual dispersion. The open field analysis supported behavioral specificity because the sedative diazepam control reduced locomotion, whereas SBEO, PDEO, and ROEO did not differ from the vehicle control.

Table S10B. Prespecified pairwise contrasts, confidence intervals, and standardized effect sizes

The contrasts below correspond to the prespecified post hoc comparisons used for dose-response, locomotor-control, and antagonist-coadministration analyses. Dose-response and locomotor-control comparisons use Dunnett-adjusted p values; antagonist-coadministration comparisons use Sidak-adjusted p values.

ID	Phase	Oil	Test/outcome	Contrast	Mean 1	Mean 2	Mean diff (95% CI)	p	Hedges' g (Mean 2 - Mean 1)
A019	Dose-response anxiety-like	PDEO	EPM; % open-arm entries	CTRL vs. 100	24.24	51.02	-26.78 (-28.64 to -24.92)	<0.0001	17.43
A019	Dose-response anxiety-like	PDEO	EPM; % open-arm entries	CTRL vs. 25	24.24	31.62	-7.38 (-9.23 to -5.52)	<0.0001	4.80
A019	Dose-response anxiety-like	PDEO	EPM; % open-arm entries	CTRL vs. 50	24.24	42.25	-18.01 (-19.86 to -16.15)	<0.0001	11.72
A019	Dose-response anxiety-like	PDEO	EPM; % open-arm entries	CTRL vs. DZP	24.24	58.82	-34.58 (-36.44 to -32.72)	<0.0001	22.50
A020	Dose-response anxiety-like	PDEO	EPM; Open-arm time (%)	CTRL vs. 100	31.42	56.37	-24.95 (-28.57 to -21.33)	<0.0001	8.33
A020	Dose-response anxiety-like	PDEO	EPM; Open-arm time (%)	CTRL vs. 25	31.42	37.08	-5.66 (-9.28 to -2.04)	0.0012	1.89
A020	Dose-response anxiety-like	PDEO	EPM; Open-arm time (%)	CTRL vs. 50	31.42	43.59	-12.17 (-15.79 to -8.55)	<0.0001	4.06
A020	Dose-response anxiety-like	PDEO	EPM; Open-arm time (%)	CTRL vs. DZP	31.42	64.45	-33.03 (-36.65 to -29.41)	<0.0001	11.03
A021	Dose-response anxiety-like	PDEO	LDB; Light-compartment time (%)	CTRL vs. 100	29.43	40.21	-10.78 (-13.53 to -8.03)	<0.0001	4.75
A021	Dose-response anxiety-like	PDEO	LDB; Light-compartment time (%)	CTRL vs. 25	29.43	30.85	-1.43 (-4.17 to 1.32)	0.4878	0.63
A021	Dose-response anxiety-like	PDEO	LDB; Light-compartment time (%)	CTRL vs. 50	29.43	35.27	-5.84 (-8.59 to -3.1)	<0.0001	2.57
A021	Dose-response anxiety-like	PDEO	LDB; Light-compartment time (%)	CTRL vs. DZP	29.43	53.79	-24.37 (-27.11 to -21.62)	<0.0001	10.72
A022	Dose-response anxiety-like	PDEO	MBT; Buried marbles (n)	CTRL vs. 100	9.62	6	3.62 (2.19 to 5.05)	<0.0001	-3.07
A022	Dose-response anxiety-like	PDEO	MBT; Buried marbles (n)	CTRL vs. 25	9.62	8.75	0.88 (-0.56 to 2.31)	0.346	-0.74
A022	Dose-response anxiety-like	PDEO	MBT; Buried marbles (n)	CTRL vs. 50	9.62	7.25	2.38 (0.94 to 3.81)	0.0006	-2.01
A022	Dose-response anxiety-like	PDEO	MBT; Buried marbles (n)	CTRL vs. DZP	9.62	2.88	6.75 (5.32 to 8.18)	<0.0001	-5.71
A023	Dose-response anxiety-like	ROEO	EPM; % open-arm entries	CTRL vs. 100	22.81	38.78	-15.97 (-18.13 to -13.8)	<0.0001	8.93

ID	Phase	Oil	Test/outcome	Contrast	Mean 1	Mean 2	Mean diff (95% CI)	p	Hedges' g (Mean 2 - Mean 1)
A023	Dose-response anxiety-like	ROEO	EPM; % open-arm entries	CTRL vs. 25	22.81	25.79	-2.98 (-5.14 to -0.81)	0.0044	1.67
A023	Dose-response anxiety-like	ROEO	EPM; % open-arm entries	CTRL vs. 50	22.81	31.47	-8.66 (-10.82 to -6.5)	<0.0001	4.84
A023	Dose-response anxiety-like	ROEO	EPM; % open-arm entries	CTRL vs. DZP	22.81	57.98	-35.17 (-37.33 to -33.01)	<0.0001	19.66
A024	Dose-response anxiety-like	ROEO	EPM; Open-arm time (%)	CTRL vs. 100	31.42	44.15	-12.73 (-16.94 to -8.52)	<0.0001	3.66
A024	Dose-response anxiety-like	ROEO	EPM; Open-arm time (%)	CTRL vs. 25	31.42	35.85	-4.43 (-8.64 to -0.22)	0.0368	1.27
A024	Dose-response anxiety-like	ROEO	EPM; Open-arm time (%)	CTRL vs. 50	31.42	37.96	-6.54 (-10.74 to -2.33)	0.0013	1.88
A024	Dose-response anxiety-like	ROEO	EPM; Open-arm time (%)	CTRL vs. DZP	31.42	66.58	-35.15 (-39.36 to -30.94)	<0.0001	10.10
A025	Dose-response anxiety-like	ROEO	LDB; Light-compartment time (%)	CTRL vs. 100	29.43	39.49	-10.07 (-12.68 to -7.45)	<0.0001	4.65
A025	Dose-response anxiety-like	ROEO	LDB; Light-compartment time (%)	CTRL vs. 25	29.43	31.08	-1.65 (-4.27 to 0.96)	0.3194	0.76
A025	Dose-response anxiety-like	ROEO	LDB; Light-compartment time (%)	CTRL vs. 50	29.43	34.63	-5.2 (-7.82 to -2.59)	<0.0001	2.41
A025	Dose-response anxiety-like	ROEO	LDB; Light-compartment time (%)	CTRL vs. DZP	29.43	53.79	-24.37 (-26.98 to -21.75)	<0.0001	11.27
A026	Dose-response anxiety-like	ROEO	MBT; Buried marbles (n)	CTRL vs. 100	8.62	4.88	3.75 (2.43 to 5.07)	<0.0001	-3.44
A026	Dose-response anxiety-like	ROEO	MBT; Buried marbles (n)	CTRL vs. 25	8.62	7.25	1.38 (0.06 to 2.69)	0.039	-1.26
A026	Dose-response anxiety-like	ROEO	MBT; Buried marbles (n)	CTRL vs. 50	8.62	6.25	2.38 (1.06 to 3.69)	0.0002	-2.18
A026	Dose-response anxiety-like	ROEO	MBT; Buried marbles (n)	CTRL vs. DZP	8.62	0.75	7.88 (6.56 to 9.19)	<0.0001	-7.22
A027	Dose-response anxiety-like	SBEO	EPM; % open-arm entries	CTRL vs. 100	25.27	53.07	-27.81 (-29.18 to -26.43)	<0.0001	24.49
A027	Dose-response anxiety-like	SBEO	EPM; % open-arm entries	CTRL vs. 25	25.27	37.07	-11.8 (-13.17 to -10.42)	<0.0001	10.39
A027	Dose-response anxiety-like	SBEO	EPM; % open-arm entries	CTRL vs. 50	25.27	45.63	-20.36 (-21.74 to -18.99)	<0.0001	17.93
A027	Dose-response anxiety-like	SBEO	EPM; % open-arm entries	CTRL vs. DZP	25.27	61.7	-36.43 (-37.8 to -35.05)	<0.0001	32.09
A028	Dose-response anxiety-like	SBEO	EPM; Open-arm time (%)	CTRL vs. 100	32.67	57.07	-24.4 (-27.73 to -21.06)	<0.0001	8.86
A028	Dose-response anxiety-like	SBEO	EPM; Open-arm time (%)	CTRL vs. 25	32.67	39.25	-6.58 (-9.91 to -3.24)	<0.0001	2.39
A028	Dose-response anxiety-like	SBEO	EPM; Open-arm time (%)	CTRL vs. 50	32.67	45.38	-12.71 (-16.04 to -9.38)	<0.0001	4.61
A028	Dose-response anxiety-like	SBEO	EPM; Open-arm time (%)	CTRL vs. DZP	32.67	66.09	-33.41 (-36.75 to -30.08)	<0.0001	12.13

ID	Phase	Oil	Test/outcome	Contrast	Mean 1	Mean 2	Mean diff (95% CI)	p	Hedges' g (Mean 2 - Mean 1)
A029	Dose-response anxiety-like	SBEO	LDB; Light-compartment time (%)	CTRL vs. 100	29.93	48.61	-18.68 (-20.98 to -16.39)	<0.0001	9.84
A029	Dose-response anxiety-like	SBEO	LDB; Light-compartment time (%)	CTRL vs. 25	29.93	32.41	-2.48 (-4.78 to -0.19)	0.0307	1.31
A029	Dose-response anxiety-like	SBEO	LDB; Light-compartment time (%)	CTRL vs. 50	29.93	38.99	-9.06 (-11.36 to -6.76)	<0.0001	4.77
A029	Dose-response anxiety-like	SBEO	LDB; Light-compartment time (%)	CTRL vs. DZP	29.93	55.17	-25.24 (-27.54 to -22.94)	<0.0001	13.30
A030	Dose-response anxiety-like	SBEO	MBT; Buried marbles (n)	CTRL vs. 100	8.75	2.62	6.12 (4.79 to 7.46)	<0.0001	-5.56
A030	Dose-response anxiety-like	SBEO	MBT; Buried marbles (n)	CTRL vs. 25	8.75	6.62	2.12 (0.79 to 3.46)	0.0009	-1.93
A030	Dose-response anxiety-like	SBEO	MBT; Buried marbles (n)	CTRL vs. 50	8.75	4.62	4.12 (2.79 to 5.46)	<0.0001	-3.74
A030	Dose-response anxiety-like	SBEO	MBT; Buried marbles (n)	CTRL vs. DZP	8.75	1.12	7.62 (6.29 to 8.96)	<0.0001	-6.92
A013	Dose-response depression-like	PDEO	FST; Immobility (s)	CTRL vs. 100	162.3	141.1	21.13 (18.28 to 23.97)	<0.0001	-9.00
A013	Dose-response depression-like	PDEO	FST; Immobility (s)	CTRL vs. 25	162.3	157.5	4.75 (1.9 to 7.6)	0.0006	-2.04
A013	Dose-response depression-like	PDEO	FST; Immobility (s)	CTRL vs. 50	162.3	150.3	12 (9.15 to 14.85)	<0.0001	-5.09
A013	Dose-response depression-like	PDEO	FST; Immobility (s)	CTRL vs. FLX	162.3	55.38	106.9 (104.0 to 109.7)	<0.0001	-45.37
A014	Dose-response depression-like	PDEO	TST; Immobility (s)	CTRL vs. 100	145.8	117.9	27.88 (25.07 to 30.68)	<0.0001	-12.03
A014	Dose-response depression-like	PDEO	TST; Immobility (s)	CTRL vs. 25	145.8	137.9	7.88 (5.07 to 10.68)	<0.0001	-3.41
A014	Dose-response depression-like	PDEO	TST; Immobility (s)	CTRL vs. 50	145.8	128.8	17 (14.2 to 19.8)	<0.0001	-7.33
A014	Dose-response depression-like	PDEO	TST; Immobility (s)	CTRL vs. FLX	145.8	64.25	81.5 (78.7 to 84.3)	<0.0001	-35.17
A015	Dose-response depression-like	ROEO	FST; Immobility (s)	CTRL vs. 100	161.9	142.9	19 (16.5 to 21.5)	<0.0001	-9.18
A015	Dose-response depression-like	ROEO	FST; Immobility (s)	CTRL vs. 25	161.9	156.0	5.88 (3.37 to 8.38)	<0.0001	-2.85
A015	Dose-response depression-like	ROEO	FST; Immobility (s)	CTRL vs. 50	161.9	149.8	12.13 (9.62 to 14.63)	<0.0001	-5.84
A015	Dose-response depression-like	ROEO	FST; Immobility (s)	CTRL vs. FLX	161.9	55.13	106.8 (104.2 to 109.3)	<0.0001	-51.57
A016	Dose-response depression-like	ROEO	TST; Immobility (s)	CTRL vs. 100	145.9	129.0	16.88 (14.74 to 19.01)	<0.0001	-9.56
A016	Dose-response depression-like	ROEO	TST; Immobility (s)	CTRL vs. 25	145.9	140.8	5.12 (2.99 to 7.26)	<0.0001	-2.89
A016	Dose-response depression-like	ROEO	TST; Immobility (s)	CTRL vs. 50	145.9	134.3	11.63 (9.49 to 13.76)	<0.0001	-6.56

ID	Phase	Oil	Test/outcome	Contrast	Mean 1	Mean 2	Mean diff (95% CI)	p	Hedges' g (Mean 2 - Mean 1)
A016	Dose-response depression-like	ROEO	TST; Immobility (s)	CTRL vs. FLX	145.9	64.38	81.5 (79.36 to 83.64)	<0.0001	-46.12
A017	Dose-response depression-like	SBEO	FST; Immobility (s)	CTRL vs. 100	156.5	132.5	24 (21.73 to 26.27)	<0.0001	-12.81
A017	Dose-response depression-like	SBEO	FST; Immobility (s)	CTRL vs. 25	156.5	148.5	8 (5.73 to 10.27)	<0.0001	-4.27
A017	Dose-response depression-like	SBEO	FST; Immobility (s)	CTRL vs. 50	156.5	141.6	14.88 (12.61 to 17.14)	<0.0001	-7.95
A017	Dose-response depression-like	SBEO	FST; Immobility (s)	CTRL vs. FLX	156.5	62.5	94 (91.73 to 96.27)	<0.0001	-50.16
A018	Dose-response depression-like	SBEO	TST; Immobility (s)	CTRL vs. 100	144.3	123.6	20.63 (18.52 to 22.73)	<0.0001	-11.88
A018	Dose-response depression-like	SBEO	TST; Immobility (s)	CTRL vs. 25	144.3	138.1	6.12 (4.02 to 8.23)	<0.0001	-3.56
A018	Dose-response depression-like	SBEO	TST; Immobility (s)	CTRL vs. 50	144.3	130.6	13.63 (11.52 to 15.73)	<0.0001	-7.86
A018	Dose-response depression-like	SBEO	TST; Immobility (s)	CTRL vs. FLX	144.3	67.13	77.13 (75.02 to 79.23)	<0.0001	-44.29
A031	Locomotor control	All oils	OFT; Line crossings	CTRL vs. DZP	31.13	8.38	22.75 (20.3 to 25.2)	<0.0001	-12.21
A031	Locomotor control	All oils	OFT; Line crossings	CTRL vs. PDEO 100	31.13	29.13	2 (-0.45 to 4.45)	0.1641	-1.07
A031	Locomotor control	All oils	OFT; Line crossings	CTRL vs. PDEO 25	31.13	32.25	-1.12 (-3.58 to 1.33)	0.7665	0.60
A031	Locomotor control	All oils	OFT; Line crossings	CTRL vs. PDEO 50	31.13	30.25	0.88 (-1.58 to 3.33)	0.9262	-0.47
A031	Locomotor control	All oils	OFT; Line crossings	CTRL vs. ROEO 100	31.13	29.38	1.75 (-0.7 to 4.2)	0.2852	-0.94
A031	Locomotor control	All oils	OFT; Line crossings	CTRL vs. ROEO 25	31.13	30.25	0.88 (-1.58 to 3.33)	0.9262	-0.47
A031	Locomotor control	All oils	OFT; Line crossings	CTRL vs. ROEO 50	31.13	29.75	1.38 (-1.08 to 3.83)	0.5576	-0.74
A031	Locomotor control	All oils	OFT; Line crossings	CTRL vs. SBEO 100	31.13	30.5	0.62 (-1.83 to 3.08)	0.9909	-0.34
A031	Locomotor control	All oils	OFT; Line crossings	CTRL vs. SBEO 25	31.13	30.88	0.25 (-2.2 to 2.7)	0.9996	-0.13
A031	Locomotor control	All oils	OFT; Line crossings	CTRL vs. SBEO 50	31.13	31.88	-0.75 (-3.2 to 1.7)	0.9699	0.40
A007	Antagonist coadministration anxiety-like	PDEO	EPM; % open-arm entries	CTRL vs. DZP	24.2	58.8	-34.6 (-37.53 to -31.67)	<0.0001	15.76
A007	Antagonist coadministration anxiety-like	PDEO	EPM; % open-arm entries	CTRL vs. EO100	24.2	51	-26.8 (-29.73 to -23.87)	<0.0001	12.21
A007	Antagonist coadministration anxiety-like	PDEO	EPM; % open-arm entries	CTRL vs. EO+FMZ	24.2	38.3	-14.1 (-17.03 to -11.17)	<0.0001	6.42
A007	Antagonist coadministration anxiety-like	PDEO	EPM; % open-arm entries	CTRL vs. EO+WAY	24.2	36.8	-12.6 (-15.53 to -9.67)	<0.0001	5.74
A007	Antagonist coadministration anxiety-like	PDEO	EPM; % open-arm entries	DZP vs. DZP+FMZ	58.8	29.1	29.7 (26.77 to 32.63)	<0.0001	-13.53
A007	Antagonist coadministration anxiety-like	PDEO	EPM; % open-arm entries	EO100 vs. EO+FMZ	51	38.3	12.7 (9.77 to 15.63)	<0.0001	-5.79

ID	Phase	Oil	Test/outcome	Contrast	Mean 1	Mean 2	Mean diff (95% CI)	p	Hedges' g (Mean 2 - Mean 1)
A007	Antagonist coadministration anxiety-like	PDEO	EPM; % open-arm entries	EO100 vs. EO+WAY	51	36.8	14.2 (11.27 to 17.13)	<0.0001	-6.47
A008	Antagonist coadministration anxiety-like	PDEO	EPM; Open-arm time (%)	CTRL vs. DZP	31.4	64.5	-33.1 (-38.03 to -28.17)	<0.0001	8.95
A008	Antagonist coadministration anxiety-like	PDEO	EPM; Open-arm time (%)	CTRL vs. EO100	31.4	56.4	-25 (-29.93 to -20.07)	<0.0001	6.76
A008	Antagonist coadministration anxiety-like	PDEO	EPM; Open-arm time (%)	CTRL vs. EO+FMZ	31.4	43.8	-12.4 (-17.33 to -7.47)	<0.0001	3.35
A008	Antagonist coadministration anxiety-like	PDEO	EPM; Open-arm time (%)	CTRL vs. EO+WAY	31.4	41.5	-10.1 (-15.03 to -5.17)	<0.0001	2.73
A008	Antagonist coadministration anxiety-like	PDEO	EPM; Open-arm time (%)	DZP vs. DZP+FMZ	64.5	35	29.5 (24.57 to 34.43)	<0.0001	-7.98
A008	Antagonist coadministration anxiety-like	PDEO	EPM; Open-arm time (%)	EO100 vs. EO+FMZ	56.4	43.8	12.6 (7.67 to 17.53)	<0.0001	-3.41
A008	Antagonist coadministration anxiety-like	PDEO	EPM; Open-arm time (%)	EO100 vs. EO+WAY	56.4	41.5	14.9 (9.97 to 19.83)	<0.0001	-4.03
A009	Antagonist coadministration anxiety-like	ROEO	EPM; % open-arm entries	CTRL vs. DZP	22.8	58	-35.2 (-38.37 to -32.03)	<0.0001	14.80
A009	Antagonist coadministration anxiety-like	ROEO	EPM; % open-arm entries	CTRL vs. EO100	22.8	39.18	-16.38 (-19.55 to -13.2)	<0.0001	6.89
A009	Antagonist coadministration anxiety-like	ROEO	EPM; % open-arm entries	CTRL vs. EO+FMZ	22.8	27.9	-5.1 (-8.27 to -1.93)	0.0003	2.14
A009	Antagonist coadministration anxiety-like	ROEO	EPM; % open-arm entries	CTRL vs. EO+WAY	22.8	35.3	-12.5 (-15.67 to -9.33)	<0.0001	5.26
A009	Antagonist coadministration anxiety-like	ROEO	EPM; % open-arm entries	DZP vs. DZP+FMZ	58	28.7	29.3 (26.13 to 32.47)	<0.0001	-12.32
A009	Antagonist coadministration anxiety-like	ROEO	EPM; % open-arm entries	EO100 vs. EO+FMZ	39.18	27.9	11.28 (8.11 to 14.45)	<0.0001	-4.74
A009	Antagonist coadministration anxiety-like	ROEO	EPM; % open-arm entries	EO100 vs. EO+WAY	39.18	35.3	3.88 (0.7 to 7.04)	0.0091	-1.63

ID	Phase	Oil	Test/outcome	Contrast	Mean 1	Mean 2	Mean diff (95% CI)	p	Hedges' g (Mean 2 - Mean 1)
A010	Antagonist coadministration anxiety-like	ROEO	EPM; Open-arm time (%)	CTRL vs. DZP	31.4	66.6	-35.2 (-40.67 to -29.73)	<0.0001	8.57
A010	Antagonist coadministration anxiety-like	ROEO	EPM; Open-arm time (%)	CTRL vs. EO100	31.4	42.9	-11.5 (-16.97 to -6.03)	<0.0001	2.80
A010	Antagonist coadministration anxiety-like	ROEO	EPM; Open-arm time (%)	CTRL vs. EO+FMZ	31.4	34	-2.6 (-8.07 to 2.87)	0.7664	0.63
A010	Antagonist coadministration anxiety-like	ROEO	EPM; Open-arm time (%)	CTRL vs. EO+WAY	31.4	39.5	-8.1 (-13.57 to -2.63)	0.001	1.97
A010	Antagonist coadministration anxiety-like	ROEO	EPM; Open-arm time (%)	DZP vs. DZP+FMZ	66.6	35.4	31.2 (25.73 to 36.67)	<0.0001	-7.60
A010	Antagonist coadministration anxiety-like	ROEO	EPM; Open-arm time (%)	EO100 vs. EO+FMZ	42.9	34	8.9 (3.43 to 14.37)	0.0003	-2.17
A010	Antagonist coadministration anxiety-like	ROEO	EPM; Open-arm time (%)	EO100 vs. EO+WAY	42.9	39.5	3.4 (-2.07 to 8.87)	0.4717	-0.83
A011	Antagonist coadministration anxiety-like	SBEO	EPM; % open-arm entries	CTRL vs. DZP	25.3	61.7	-36.4 (-38.72 to -34.08)	<0.0001	20.89
A011	Antagonist coadministration anxiety-like	SBEO	EPM; % open-arm entries	CTRL vs. EO100	25.3	53.31	-28.01 (-30.34 to -25.69)	<0.0001	16.07
A011	Antagonist coadministration anxiety-like	SBEO	EPM; % open-arm entries	CTRL vs. EO+FMZ	25.3	31.4	-6.1 (-8.42 to -3.78)	<0.0001	3.50
A011	Antagonist coadministration anxiety-like	SBEO	EPM; % open-arm entries	CTRL vs. EO+WAY	25.3	48.43	-23.13 (-25.45 to -20.8)	<0.0001	13.27
A011	Antagonist coadministration anxiety-like	SBEO	EPM; % open-arm entries	DZP vs. DZP+FMZ	61.7	30.2	31.5 (29.18 to 33.82)	<0.0001	-18.08
A011	Antagonist coadministration anxiety-like	SBEO	EPM; % open-arm entries	EO100 vs. EO+FMZ	53.31	31.4	21.91 (19.59 to 24.24)	<0.0001	-12.57
A011	Antagonist coadministration anxiety-like	SBEO	EPM; % open-arm entries	EO100 vs. EO+WAY	53.31	48.43	4.89 (2.56 to 7.21)	<0.0001	-2.80
A012	Antagonist coadministration anxiety-like	SBEO	EPM; Open-arm time (%)	CTRL vs. DZP	32.7	66.1	-33.4 (-38.64 to -28.16)	<0.0001	8.49

ID	Phase	Oil	Test/outcome	Contrast	Mean 1	Mean 2	Mean diff (95% CI)	p	Hedges' g (Mean 2 - Mean 1)
A012	Antagonist coadministration anxiety-like	SBEO	EPM; Open-arm time (%)	CTRL vs. EO100	32.7	57.1	-24.4 (-29.64 to -19.16)	<0.0001	6.20
A012	Antagonist coadministration anxiety-like	SBEO	EPM; Open-arm time (%)	CTRL vs. EO+FMZ	32.7	37.6	-4.9 (-10.14 to 0.34)	0.0794	1.25
A012	Antagonist coadministration anxiety-like	SBEO	EPM; Open-arm time (%)	CTRL vs. EO+WAY	32.7	51.4	-18.7 (-23.94 to -13.46)	<0.0001	4.75
A012	Antagonist coadministration anxiety-like	SBEO	EPM; Open-arm time (%)	DZP vs. DZP+FMZ	66.1	35.6	30.5 (25.26 to 35.74)	<0.0001	-7.75
A012	Antagonist coadministration anxiety-like	SBEO	EPM; Open-arm time (%)	EO100 vs. EO+FMZ	57.1	37.6	19.5 (14.26 to 24.74)	<0.0001	-4.96
A012	Antagonist coadministration anxiety-like	SBEO	EPM; Open-arm time (%)	EO100 vs. EO+WAY	57.1	51.4	5.7 (0.46 to 10.94)	0.0263	-1.45
A001	Antagonist coadministration depression-like	PDEO	FST; Immobility (s)	CTRL vs. EO100	162.3	141.1	21.2 (16.04 to 26.36)	<0.0001	-5.48
A001	Antagonist coadministration depression-like	PDEO	FST; Immobility (s)	CTRL vs. EO+WAY	162.3	155.0	7.3 (2.14 to 12.46)	0.0018	-1.89
A001	Antagonist coadministration depression-like	PDEO	FST; Immobility (s)	CTRL vs. FLX	162.3	55.6	106.7 (101.5 to 111.9)	<0.0001	-27.56
A001	Antagonist coadministration depression-like	PDEO	FST; Immobility (s)	CTRL vs. WAY	162.3	163.0	-0.7 (-5.86 to 4.46)	0.9998	0.18
A001	Antagonist coadministration depression-like	PDEO	FST; Immobility (s)	EO100 vs. EO+WAY	141.1	155.0	-13.9 (-19.06 to -8.74)	<0.0001	3.59
A001	Antagonist coadministration depression-like	PDEO	FST; Immobility (s)	FLX vs. FLX+WAY	55.6	116.0	-60.4 (-65.56 to -55.24)	<0.0001	15.60
A002	Antagonist coadministration depression-like	PDEO	TST; Immobility (s)	CTRL vs. EO100	145.8	118.0	27.8 (23.3 to 32.3)	<0.0001	-8.24
A002	Antagonist coadministration depression-like	PDEO	TST; Immobility (s)	CTRL vs. EO+WAY	145.8	139.5	6.3 (1.8 to 10.8)	0.0021	-1.87
A002	Antagonist coadministration depression-like	PDEO	TST; Immobility (s)	CTRL vs. FLX	145.8	64.3	81.5 (77 to 86)	<0.0001	-24.15

ID	Phase	Oil	Test/outcome	Contrast	Mean 1	Mean 2	Mean diff (95% CI)	p	Hedges' g (Mean 2 - Mean 1)
A002	Antagonist coadministration depression-like	PDEO	TST; Immobility (s)	CTRL vs. WAY	145.8	146.0	-0.2 (-4.7 to 4.3)	>0.9999	0.06
A002	Antagonist coadministration depression-like	PDEO	TST; Immobility (s)	EO100 vs. EO+WAY	118.0	139.5	-21.5 (-26 to -17)	<0.0001	6.37
A002	Antagonist coadministration depression-like	PDEO	TST; Immobility (s)	FLX vs. FLX+WAY	64.3	112.5	-48.2 (-52.7 to -43.7)	<0.0001	14.28
A003	Antagonist coadministration depression-like	ROEO	FST; Immobility (s)	CTRL vs. EO100	161.9	142.9	19 (14.18 to 23.82)	<0.0001	-5.26
A003	Antagonist coadministration depression-like	ROEO	FST; Immobility (s)	CTRL vs. EO+WAY	161.9	154.0	7.9 (3.08 to 12.72)	0.0002	-2.19
A003	Antagonist coadministration depression-like	ROEO	FST; Immobility (s)	CTRL vs. FLX	161.9	55.1	106.8 (102.0 to 111.6)	<0.0001	-29.56
A003	Antagonist coadministration depression-like	ROEO	FST; Immobility (s)	CTRL vs. WAY	161.9	162.5	-0.6 (-5.42 to 4.22)	0.9999	0.17
A003	Antagonist coadministration depression-like	ROEO	FST; Immobility (s)	EO100 vs. EO+WAY	142.9	154.0	-11.1 (-15.92 to -6.28)	<0.0001	3.07
A003	Antagonist coadministration depression-like	ROEO	FST; Immobility (s)	FLX vs. FLX+WAY	55.1	114.0	-58.9 (-63.72 to -54.08)	<0.0001	16.30
A004	Antagonist coadministration depression-like	ROEO	TST; Immobility (s)	CTRL vs. EO100	145.9	129.0	16.9 (12.75 to 21.05)	<0.0001	-5.43
A004	Antagonist coadministration depression-like	ROEO	TST; Immobility (s)	CTRL vs. EO+WAY	145.9	140.5	5.4 (1.25 to 9.55)	0.0048	-1.73
A004	Antagonist coadministration depression-like	ROEO	TST; Immobility (s)	CTRL vs. FLX	145.9	64.4	81.5 (77.35 to 85.65)	<0.0001	-26.17
A004	Antagonist coadministration depression-like	ROEO	TST; Immobility (s)	CTRL vs. WAY	145.9	146.4	-0.5 (-4.65 to 3.65)	>0.9999	0.16
A004	Antagonist coadministration depression-like	ROEO	TST; Immobility (s)	EO100 vs. EO+WAY	129.0	140.5	-11.5 (-15.65 to -7.35)	<0.0001	3.69
A004	Antagonist coadministration depression-like	ROEO	TST; Immobility (s)	FLX vs. FLX+WAY	64.4	111.0	-46.6 (-50.75 to -42.45)	<0.0001	14.96

ID	Phase	Oil	Test/outcome	Contrast	Mean 1	Mean 2	Mean diff (95% CI)	p	Hedges' g (Mean 2 - Mean 1)
A005	Antagonist coadministration depression-like	SBEO	FST; Immobility (s)	CTRL vs. EO100	156.5	132.5	24 (19.03 to 28.97)	<0.0001	-6.43
A005	Antagonist coadministration depression-like	SBEO	FST; Immobility (s)	CTRL vs. EO+WAY	156.5	146.0	10.5 (5.53 to 15.47)	<0.0001	-2.81
A005	Antagonist coadministration depression-like	SBEO	FST; Immobility (s)	CTRL vs. FLX	156.5	62.5	94 (89.03 to 98.97)	<0.0001	-25.20
A005	Antagonist coadministration depression-like	SBEO	FST; Immobility (s)	CTRL vs. WAY	156.5	157.0	-0.5 (-5.47 to 4.47)	>0.9999	0.13
A005	Antagonist coadministration depression-like	SBEO	FST; Immobility (s)	EO100 vs. EO+WAY	132.5	146.0	-13.5 (-18.47 to -8.53)	<0.0001	3.62
A005	Antagonist coadministration depression-like	SBEO	FST; Immobility (s)	FLX vs. FLX+WAY	62.5	118.0	-55.5 (-60.47 to -50.53)	<0.0001	14.88
A006	Antagonist coadministration depression-like	SBEO	TST; Immobility (s)	CTRL vs. EO100	144.3	123.6	20.7 (16.65 to 24.75)	<0.0001	-6.67
A006	Antagonist coadministration depression-like	SBEO	TST; Immobility (s)	CTRL vs. EO+WAY	144.3	138.0	6.3 (2.25 to 10.35)	0.0006	-2.03
A006	Antagonist coadministration depression-like	SBEO	TST; Immobility (s)	CTRL vs. FLX	144.3	67.1	77.2 (73.15 to 81.25)	<0.0001	-24.89
A006	Antagonist coadministration depression-like	SBEO	TST; Immobility (s)	CTRL vs. WAY	144.3	145.0	-0.7 (-4.75 to 3.35)	0.9977	0.23
A006	Antagonist coadministration depression-like	SBEO	TST; Immobility (s)	EO100 vs. EO+WAY	123.6	138.0	-14.4 (-18.45 to -10.35)	<0.0001	4.64
A006	Antagonist coadministration depression-like	SBEO	TST; Immobility (s)	FLX vs. FLX+WAY	67.1	112.0	-44.9 (-48.95 to -40.85)	<0.0001	14.48

Note. Mean diff = Mean 1 minus Mean 2. Hedges' g is reported as a directional, small-sample-corrected standardized mean difference calculated using the residual root mean square error from the corresponding ANOVA model; negative values indicate that Mean 2 was lower than Mean 1, whereas positive values indicate that Mean 2 was higher than Mean 1. Abbreviations: SBEO, Satureja brevicalyx essential oil; PDEO, Peperomia dolabriformis essential oil; ROEO, Rosmarinus officinalis essential oil; EPM, elevated plus maze; LDB, light-dark box; MBT, marble burying test; TST, tail suspension test; FST, forced swim test; OFT, open field test; DZP, diazepam; FLX, fluoxetine; FMZ, flumazenil; WAY, WAY-100635; EO100, essential oil at 100 mg/kg. The prespecified contrasts support dose-related anxiolytic-like and antidepressant-like behavioral changes for the three essential oils within their respective experimental blocks, while avoiding unsupported between-oil ranking. Antagonist-coadministration contrasts support antagonist-sensitive attenuation patterns, but these findings should be interpreted as pathway-level pharmacological sensitivity rather than as evidence of direct receptor mediation.
