# Supplementary material for: Integrated Chemical, In Silico, and Functional Neurobehavioral Evaluation of Three Essential Oils in Acute Anxiety- and Depression-Related Mouse Models
Source: Molecules. 2026 Jul 6;31(13):2378. doi: 10.3390/molecules31132378 (PMC13362989; doi:10.3390/molecules31132378)
Supplement: Supplementary file 1 [file molecules-31-02378-s001.zip › Supplementary Table S2 Peperomia_dolabriformis essential oil composition.pdf]

Supplementary Table S2. Chemical composition of the essential oil of *Peperomia dolabriformis*

N°	Compound	RT (min)	RI	Area (%)
1	Nonane	6.031	900	1.02
2	α-Pinene	7.570	932	1.35
3	β-Pinene	10.226	974	1.64
4	β-Myrcene	11.608	988	0.44
5	Limonene	14.130	1024	13.60
6	(E)-β-Ocimene	15.533	1044	0.11
7	n-Butylbenzene	15.826	1068	4.25
8	2,3,5,8-Tetramethyldecane	18.718	1312	0.47
9	α-Terpineol	23.558	1186	0.08
10	Decanal	24.512	1201	0.25
11	Hexylbenzene	27.008	1255	0.34
12	Orcinol dimethyl ether	27.534	1274	4.05
13	Sesquiterpene hydrocarbon, tentative	30.683	1350*	0.90
14	α-Copaene	32.346	1374	0.98
15	β-Bourbonene	32.701	1387	0.05
16	Sesquiterpene hydrocarbon, tentative	33.125	1392*	0.45
17	5-tert-Butyl-1,3-benzodioxole	33.805	1400*	23.19
18	Cadinane-type sesquiterpene, tentative	33.895	1405*	0.10
19	Dodecanal (lauraldehyde)	34.019	1408	0.05
20	β-Caryophyllene	34.265	1417	0.89
21	Selina-5,11-diene	34.901	1445	0.06
22	cis-Eudesma-6,11-diene	35.235	1490	0.11
23	α-Humulene	35.684	1452	0.35
24	Ishwarane	36.069	1468	8.83
25	Quinoline derivative, tentative	36.181	1470*	0.11
26	γ-Amorphene	36.817	1495	1.00
27	Aristolochene	36.951	1496*	0.59
28	Eremophilene	37.324	1498*	1.24
29	Bicyclogermacrene	37.466	1500	0.78
30	α-Farnesene	37.595	1505	0.06
31	Humulene isomer, tentative	37.848	1508*	0.75
32	Tricyclic sesquiterpene, tentative	37.952	1510*	0.92
33	β-Bisabolene	38.069	1505	0.97
34	α-Panasinsene	38.275	1527	0.20
35	Sesquiterpene hydrocarbon, tentative	38.455	1528*	0.14
36	Myristicin	38.779	1517	10.83
37	Sesquiterpene hydrocarbon, tentative	39.425	1535*	0.23
38	Elemicin	40.248	1555	5.49
39	Sesquiterpene alcohol, tentative	40.666	1577*	0.07
40	Alloaromadendrene	40.913	1458	0.66
41	Cadinane-type sesquiterpene, tentative	41.191	1475*	0.13
42	Aromadendrene-type sesquiterpene, tentative	41.532	1439*	0.31
43	Epiglobulol	41.974	1532	0.07
44	Oxygenated sesquiterpene, tentative	42.115	1580*	0.06
45	Oxygenated sesquiterpene, tentative	42.593	1590*	0.27
46	Eudesmane-type sesquiterpene, tentative	42.702	1490*	0.19
47	τ-Cadinol	43.236	1640	3.84
48	Cadinol isomer I, tentative	43.464	1642*	0.41
49	Cadinol isomer II, tentative	43.665	1640	0.08
50	β-Guaiene	44.008	1492	0.32

N°	Compound	RT (min)	RI	Area (%)
51	Naphthalenone derivative, tentative	44.145	1648*	0.05
52	Ishwarol B	44.390	1674	6.11
53	Sesquiterpene hydrocarbon, tentative	44.487	1502*	0.15
54	Humulene isomer, tentative	44.793	1452*	0.12
55	Aromadendrene-type sesquiterpene, tentative	45.259	1439*	0.27

Notes. Compound names were standardized according to commonly used essential-oil nomenclature when possible. RI values are indicative literature/reference retention indices for non-polar or slightly polar 5%-phenyl-methylpolysiloxane columns and should not be interpreted as experimentally calculated RI values because a homologous n-alkane series was not acquired under identical chromatographic conditions. Peaks marked with an asterisk (*) are tentative or RT/RI-estimated assignments. Multiple cadinol-like peaks were annotated separately as tentative isomers when their retention behavior and mass spectral similarity suggested related but non-identical oxygenated sesquiterpenes. Compound assignments were based on mass spectral library matching and comparison with reference RI values from NIST Chemistry WebBook, Babushok et al. (2011), Adams' database, and other literature sources [84–86].
