# Supplementary material for: Integrated Chemical, In Silico, and Functional Neurobehavioral Evaluation of Three Essential Oils in Acute Anxiety- and Depression-Related Mouse Models
Source: Molecules. 2026 Jul 6;31(13):2378. doi: 10.3390/molecules31132378 (PMC13362989; doi:10.3390/molecules31132378)
Supplement: Supplementary file 1 [file molecules-31-02378-s001.zip › Supplementary Table S3 Rosmarinus_officinalis essential oil composition.pdf]

Supplementary Table S3. Chemical composition of *Rosmarinus officinalis* essential oil

N°	Compound	RT (min)	RI	Area (%)
1	Tricyclene	12.865	921	0.42
2	α-Thujene	13.243	930	0.20
3	α-Pinene	13.707	939	7.32
4	Camphene	14.202	954	4.02
5	Dehydrosabinene / thujadiene (tentative)	14.611	968*	0.12
6	β-Pinene	14.721	979	4.93
7	1-Octen-3-ol	14.980	979	0.13
8	3-Octanone	15.128	986	0.12
9	β-Myrcene	15.250	991	1.39
10	3-Octanol	15.567	995	0.05
11	α-Phellandrene	15.780	1003	0.16
12	α-Terpinene	15.930	1017	0.04
13	o-Cymene	16.189	1026	0.07
14	Limonene	16.307	1029	10.81
15	1,8-Cineole (eucalyptol)	16.365	1033	27.40
16	Phenylacetaldehyde	16.506	1044	0.05
17	γ-Terpinene	16.749	1060	0.57
18	Thujanol / sabinene hydrate (tentative)	17.075	1065*	0.13
19	Terpinolene	17.222	1088	0.11
20	Monoterpene hydrocarbon, 2-carene-like (tentative; RI mismatch)	17.272	1090*	0.10
21	Linalool	17.887	1098	6.75
22	Chrysanthenone	20.638	1125	0.16
23	Camphor	20.900	1145	5.05
24	Carveol-like monoterpenoid (tentative; RI mismatch)	21.193	1150*	0.15
25	Cyclohexylethyl trifluoroacetate (probable artefact, tentative)	21.451	1154*	0.36
26	Pinocarvone	21.909	1162	0.97
27	Borneol	22.942	1169	0.73
28	α-Phellandren-8-ol (tentative)	23.115	1172*	0.27
29	Pinocamphone / 3-pinanone (tentative)	23.464	1175*	0.17
30	Terpinen-4-ol	23.673	1177	0.98
31	α-Terpineol	24.095	1189	16.38
32	Myrtenol	24.133	1195	0.12
33	Borneol/isoborneol isomer (tentative)	24.454	1205*	0.37
34	Verbenone	24.584	1208	0.05
35	Z-Grandlure II (tentative)	24.627	1209*	0.05
36	cis-Myrtanol	24.818	1217	0.33
37	Carvone	25.272	1243	0.44
38	Isopiperitenone	26.402	1269	0.07
39	Methyl geranate/nerolate isomer (tentative)	27.075	1280*	0.32
40	Bornyl acetate	27.747	1285	4.58
41	α-Terpinyl acetate	28.859	1349	0.15
42	Isopropenyl-methylcyclopentylmethyl acetate (tentative)	31.820	1397*	0.07
43	trans-Dihydrocarvyl acetate	32.017	1400*	0.04
44	β-Caryophyllene	33.139	1419	0.73
45	α-Humulene	33.957	1454	0.11
46	Bicyclogermacrene	34.383	1494	0.38
47	β-Bisabolene	34.469	1505	0.09
48	Myristicin	35.410	1518	0.28

N°	Compound	RT (min)	RI	Area (%)
49	Unidentified sesquiterpene hydrocarbon, cembrene-like library match (tentative; RI-inconsistent)	35.538	1523*	0.16
50	Germacren-1-ol	35.651	1574	0.25

Notes. Compound names were standardized according to commonly used essential-oil nomenclature when possible. RI values are indicative literature/reference retention indices for non-polar or slightly polar 5%-phenyl-methylpolysiloxane columns and should not be interpreted as experimentally calculated RI values because a homologous n-alkane series was not acquired under identical chromatographic conditions. Peaks marked with an asterisk (*) are tentative or RT/RI-estimated assignments. Compound assignments were based on mass spectral library matching and comparison with reference RI values from NIST Chemistry WebBook, Babushok et al. (2011), Adams' database, and other literature sources[84–86].
