# Supplementary material for: Integrated Chemical, In Silico, and Functional Neurobehavioral Evaluation of Three Essential Oils in Acute Anxiety- and Depression-Related Mouse Models
Source: Molecules. 2026 Jul 6;31(13):2378. doi: 10.3390/molecules31132378 (PMC13362989; doi:10.3390/molecules31132378)
Supplement: Supplementary file 1 [file molecules-31-02378-s001.zip › Supplementary Table S7. Complete exploratory serum corticosterone and cytokine profile.pdf]

Supplementary Table S7. Complete exploratory serum corticosterone and cytokine profile across all tested doses of the essential oils

Values are mean ± SEM (n = 8). Overall p values correspond to ordinary one-way ANOVA. Dose-specific p values correspond to Dunnett-adjusted comparisons versus vehicle. BH-FDR q values were calculated across all 54 dose-versus-vehicle biomarker comparisons.

Satureja brevicalyx

Biomarker	Vehicle mean ± SEM	EO25 mean ± SEM	Δ25 (%)	p25	q25	EO50 mean ± SEM	Δ50 (%)	p50	q50	EO100 mean ± SEM	Δ100 (%)	p100	q100	Overall ANOVA p
Corticosterone (ng/mL)	83.97 ± 1.84	83.45 ± 0.65	-0.6	0.9775	>0.9999	79.70 ± 0.76	-5.1	0.0333	0.1998	75.57 ± 0.86	-10.0	<0.0001	0.0021	<0.0001
IL-6 (pg/mL)	32.35 ± 1.36	33.47 ± 0.78	+3.5	0.7220	>0.9999	30.72 ± 0.72	-5.0	0.4583	>0.9999	28.65 ± 0.61	-11.5	0.0211	0.1762	0.0053
TNF-α (pg/mL)	15.67 ± 1.24	15.25 ± 0.61	-2.7	0.9623	>0.9999	13.13 ± 0.56	-16.2	0.0684	0.3693	11.19 ± 0.36	-28.6	0.0008	0.0227	0.0009
IL-1β (pg/mL)	30.43 ± 1.13	29.65 ± 1.06	-2.6	0.8801	>0.9999	25.42 ± 0.69	-16.5	0.0019	0.0260	26.72 ± 0.75	-12.2	0.0228	0.1762	0.0017
IL-10 (pg/mL)	25.30 ± 1.09	25.75 ± 0.63	+1.8	0.9708	>0.9999	27.37 ± 1.10	+8.2	0.2694	0.9708	29.20 ± 0.70	+15.4	0.0137	0.1476	0.0208
IL-4 (pg/mL)	19.27 ± 0.89	20.40 ± 0.61	+5.9	0.5767	>0.9999	22.15 ± 0.50	+14.9	0.0287	0.1934	23.45 ± 0.90	+21.7	0.0013	0.0236	0.0024

Peperomia dolabriformis

Biomarker	Vehicle mean ± SEM	EO25 mean ± SEM	Δ25 (%)	p25	q25	EO50 mean ± SEM	Δ50 (%)	p50	q50	EO100 mean ± SEM	Δ100 (%)	p100	q100	Overall ANOVA p
Corticosterone (ng/mL)	83.23 ± 1.61	84.89 ± 0.65	+2.0	0.8886	>0.9999	79.03 ± 2.53	-5.0	0.3404	>0.9999	79.53 ± 2.64	-4.4	0.4399	>0.9999	0.1395
IL-6 (pg/mL)	34.21 ± 1.24	33.18 ± 1.14	-3.0	0.9216	>0.9999	33.83 ± 1.49	-1.1	0.9953	>0.9999	33.67 ± 1.78	-1.6	0.9869	>0.9999	0.9659
TNF-α (pg/mL)	18.52 ± 0.70	17.22 ± 0.84	-7.1	0.4721	>0.9999	17.57 ± 0.71	-5.2	0.6953	>0.9999	16.85 ± 0.73	-9.1	0.2809	0.9708	0.4392
IL-1β (pg/mL)	31.22 ± 1.34	30.63 ± 1.18	-1.9	0.9782	>0.9999	28.71 ± 1.39	-8.0	0.4065	>0.9999	29.36 ± 1.34	-6.0	0.6311	>0.9999	0.5260
IL-10 (pg/mL)	25.14 ± 1.18	25.13 ± 0.92	-0.0	>0.9999	>0.9999	26.27 ± 1.67	+4.5	0.8962	>0.9999	25.86 ± 1.74	+2.9	0.9693	>0.9999	0.9237
IL-4 (pg/mL)	17.62 ± 1.52	19.22 ± 0.88	+9.1	0.7462	>0.9999	18.65 ± 1.39	+5.8	0.9118	>0.9999	18.31 ± 1.58	+3.9	0.9700	>0.9999	0.8684

Rosmarinus officinalis

Biomarker	Vehicle mean ± SEM	EO25 mean ± SEM	Δ25 (%)	p25	q25	EO50 mean ± SEM	Δ50 (%)	p50	q50	EO100 mean ± SEM	Δ100 (%)	p100	q100	Overall ANOVA p
Corticosterone (ng/mL)	81.98 ± 1.67	82.93 ± 0.70	+1.2	0.9755	>0.9999	76.10 ± 2.52	-7.2	0.1235	0.5563	76.81 ± 2.62	-6.3	0.1954	0.8118	0.0490
IL-6 (pg/mL)	35.35 ± 0.82	34.14 ± 0.66	-3.4	0.8377	>0.9999	34.82 ± 1.20	-1.5	0.9818	>0.9999	34.59 ± 1.95	-2.1	0.9511	>0.9999	0.9230
TNF-α (pg/mL)	16.56 ± 1.11	16.64 ± 0.89	+0.5	0.9999	>0.9999	15.23 ± 0.99	-8.1	0.7009	>0.9999	14.22 ± 1.20	-14.2	0.2876	0.9708	0.3228
IL-1β (pg/mL)	30.45 ± 1.32	29.70 ± 0.95	-2.5	0.9462	>0.9999	26.94 ± 1.33	-11.5	0.1236	0.5563	27.85 ± 1.21	-8.5	0.3152	>0.9999	0.1732
IL-10 (pg/mL)	23.53 ± 1.24	23.09 ± 0.93	-1.9	0.9940	>0.9999	25.11 ± 1.97	+6.8	0.8064	>0.9999	24.53 ± 1.77	+4.3	0.9378	>0.9999	0.7797
IL-4 (pg/mL)	18.39 ± 1.28	19.76 ± 0.67	+7.5	0.8433	>0.9999	19.83 ± 1.63	+7.8	0.8247	>0.9999	19.35 ± 1.92	+5.3	0.9357	>0.9999	0.8895

Notes. EO25/EO50/EO100 = essential oil at 25/50/100 mg/kg; SEM = standard error of the mean; BH-FDR = Benjamini-Hochberg false discovery rate; IL = interleukin; TNF-α = tumor necrosis factor alpha. Negative Δ values indicate reductions relative to vehicle; positive values indicate increases.
