# Supplementary material for: Integrated Chemical, In Silico, and Functional Neurobehavioral Evaluation of Three Essential Oils in Acute Anxiety- and Depression-Related Mouse Models
Source: Molecules. 2026 Jul 6;31(13):2378. doi: 10.3390/molecules31132378 (PMC13362989; doi:10.3390/molecules31132378)
Supplement: Supplementary file 1 [file molecules-31-02378-s001.zip › Supplementary Table S8. Complete dose-by-dose preliminary acute oral toxicity observations for the three essential oils.pdf]

Supplementary Table S8, complete dose-by-dose preliminary acute oral toxicity observations for the three essential oils

Essential oil	Phase	Dose (mg/kg, p.o.)	n	Deaths at 24 h	Mortality (%)	Main clinical observations	Inferred LD50
Satureja brevicalyx	1	10	3	0/3	0	No abnormal clinical signs	>5000 mg/kg
Satureja brevicalyx	1	100	3	0/3	0	No abnormal clinical signs	>5000 mg/kg
Satureja brevicalyx	1	1000	3	0/3	0	No abnormal clinical signs	>5000 mg/kg
Satureja brevicalyx	2	1600	3	0/3	0	Mild transient piloerection/reduced grooming in 1/3 mice; recovery within 4 h	>5000 mg/kg
Satureja brevicalyx	2	2900	3	0/3	0	Mild transient hypoactivity or piloerection in 2/3 mice; recovery within 6 h	>5000 mg/kg
Satureja brevicalyx	2	5000	3	0/3	0	Transient hypoactivity, mild ataxia, and piloerection in 3/3 mice; recovery by 24 h	>5000 mg/kg
Peperomia dolabriformis	1	10	3	0/3	0	No abnormal clinical signs	>5000 mg/kg
Peperomia dolabriformis	1	100	3	0/3	0	No abnormal clinical signs	>5000 mg/kg
Peperomia dolabriformis	1	1000	3	0/3	0	No abnormal clinical signs	>5000 mg/kg
Peperomia dolabriformis	2	1600	3	0/3	0	Mild transient hypoactivity in 1/3 mice; recovery within 4 h	>5000 mg/kg
Peperomia dolabriformis	2	2900	3	0/3	0	Mild-to-moderate transient hypoactivity/ataxia in 2/3 mice; recovery within 6 h	>5000 mg/kg
Peperomia dolabriformis	2	5000	3	0/3	0	Transient hypoactivity, mild ataxia, and reduced grooming in 3/3 mice; recovery by 24 h	>5000 mg/kg
Rosmarinus officinalis	1	10	3	0/3	0	No abnormal clinical signs	>5000 mg/kg
Rosmarinus officinalis	1	100	3	0/3	0	No abnormal clinical signs	>5000 mg/kg
Rosmarinus officinalis	1	1000	3	0/3	0	No abnormal clinical signs	>5000 mg/kg
Rosmarinus officinalis	2	1600	3	0/3	0	Mild transient hypoactivity or reduced grooming in 1/3 mice; recovery within 4 h	>5000 mg/kg
Rosmarinus officinalis	2	2900	3	0/3	0	Mild transient hypoactivity in 2/3 mice; recovery within 6 h	>5000 mg/kg
Rosmarinus officinalis	2	5000	3	0/3	0	Mild-to-moderate transient hypoactivity and piloerection in 3/3 mice; recovery by 24 h	>5000 mg/kg

Note. Preliminary acute oral toxicity was evaluated using a modified Lorke approach. The oral LD50 was estimated as greater than 5000 mg/kg because no mortality was observed at the highest tested dose within the 24-h observation window. p.o. = oral administration. Clinical observations were transient and resolved during the observation period. These data should be interpreted as preliminary acute toxicity findings and do not replace subacute, chronic, reproductive, hepatic, renal, hematological, or histopathological safety studies.
