# Supplementary material for: Integrated Chemical, In Silico, and Functional Neurobehavioral Evaluation of Three Essential Oils in Acute Anxiety- and Depression-Related Mouse Models
Source: Molecules. 2026 Jul 6;31(13):2378. doi: 10.3390/molecules31132378 (PMC13362989; doi:10.3390/molecules31132378)
Supplement: Supplementary file 1 [file molecules-31-02378-s001.zip › Supplementary Table S9. Integrated interpretation of antagonist-sensitive anxiolytic- and antidepressant-like behavioral patterns .pdf]

Supplementary Table S9. Integrated interpretation of antagonist-sensitive anxiolytic- and antidepressant-like behavioral patterns of the essential oils

Essential oil	Anxiolytic-like antagonist-sensitive pattern suggested by EPM antagonist assays	Antidepressant-like antagonist-sensitive pattern suggested by TST/FST antagonist assays	Overall pathway-level interpretation
Satureja brevicalyx	Flumazenil markedly attenuated the EO100 effect in open arm entries and time in open arms; WAY-100635 produced only mild attenuation.	WAY-100635 partially attenuated the EO100-induced reduction in immobility in both TST and FST.	Predominantly GABA-A/benzodiazepine-sensitive anxiolytic-like profile, with partial 5-HT1A-related contribution to antidepressant-like activity.
Peperomia dolabriformis	Flumazenil and WAY-100635 both attenuated the EO100-induced anxiolytic-like response, suggesting mixed GABA-A/benzodiazepine- and 5-HT1A-related pathway participation.	WAY-100635 consistently attenuated the EO100-induced reduction in immobility in TST and FST.	Multitarget profile involving GABA-A/benzodiazepine-sensitive and 5-HT1A-related pathways, with clearer dual antagonist-sensitive attenuation than observed for the other oils.
Rosmarinus officinalis	Flumazenil attenuated the EO100 effect more clearly than WAY-100635. In time spent in open arms, WAY-100635 did not significantly modify the EO100 effect.	WAY-100635 partially attenuated the EO100-induced reduction in immobility in TST and FST.	Moderate mixed profile; anxiolytic-like activity appears mainly flumazenil-sensitive, whereas antidepressant-like activity is partially 5-HT1A-related.

Note. EO100 = essential oil at 100 mg/kg; EPM = elevated plus maze; TST = tail suspension test; FST = forced swim test; WAY-100635 = selective 5-HT1A receptor antagonist. Interpretation was based on antagonist-induced attenuation of behavioral effects. Because antagonist effects were generally partial, the proposed pathway-level patterns should be interpreted as compatible with pathway participation rather than as evidence of exclusive receptor mediation.
